# *MhcII* Regulates Transmission of α-Synuclein-Seeded Pathology in Mice

**DOI:** 10.3390/ijms23158175

**Published:** 2022-07-25

**Authors:** Elsa Gonzalez De La Cruz, Quan Vo, Katie Moon, Karen N. McFarland, Mary Weinrich, Tristan Williams, Benoit I. Giasson, Paramita Chakrabarty

**Affiliations:** 1 Center for Translational Research in Neurodegenerative Disease, University of Florida, Gainesville, FL 32610, USA; elsa.gondelacruz@gmail.com (E.G.D.L.C.); vo.quan@ufl.edu (Q.V.); km121999@gmail.com (K.M.); karen.mcfarland@neurology.ufl.edu (K.N.M.); mweinrich6927@gmail.com (M.W.); williamst@ufl.edu (T.W.); bgiasson@ufl.edu (B.I.G.); 2 Department of Neurology, University of Florida, Gainesville, FL 32610, USA; 3 McKnight Brain Institute, University of Florida, Gainesville, FL 32610, USA; 4 Department of Neuroscience, University of Florida, Gainesville, FL 32610, USA

**Keywords:** synuclein, Lewy body, transmission, prion, MhcI, inflammation

## Abstract

MHCII molecules, expressed by professional antigen-presenting cells (APCs) such as T cells and B cells, are hypothesized to play a key role in the response of cellular immunity to α-synuclein (α-syn). However, the role of cellular immunity in the neuroanatomic transmission of α-syn pre-formed fibrillar (PFF) seeds is undetermined. To illuminate whether cellular immunity influences the transmission of α-syn seeds from the periphery into the CNS, we injected preformed α-syn PFFs in the hindlimb of the Line M83 transgenic mouse model of synucleinopathy lacking MhcII. We showed that a complete deficiency in MhcII accelerated the appearance of seeded α-syn pathology and shortened the lifespan of the PFF-seeded M83 mice. To characterize whether B-cell and T-cell inherent MhcII function underlies this accelerated response to PFF seeding, we next injected α-syn PFFs in Rag1−/− mice which completely lacked these mature lymphocytes. There was no alteration in the lifespan or burden of endstage α-syn pathology in the PFF-seeded, Rag1-deficient M83+/− mice. Together, these results suggested that MhcII function on immune cells other than these classical APCs is potentially involved in the propagation of α-syn in this model of experimental synucleinopathy. We focused on microglia next, finding that while microglial burden was significantly upregulated in PFF-seeded, MhcII-deficient mice relative to controls, the microglial activation marker Cd68 was reduced in these mice, suggesting that these microglia were not responsive. Additional analysis of the CNS showed the early appearance of the neurotoxic astrocyte A1 signature and the induction of the Ifnγ-inducible anti-viral response mediated by MhcI in the MhcII-deficient, PFF-seeded mice. Overall, our data suggest that the loss of MhcII function leads to a dysfunctional response in non-classical APCs and that this response could potentially play a role in determining PFF-induced pathology. Collectively, our results identify the critical role of MhcII function in synucleinopathies induced by α-syn prion seeds.

## 1. Introduction

Synucleinopathies are a spectrum of neurodegenerative diseases characterized by the aggregation of the naturally soluble α-synuclein (α-syn) protein and its propagation within the brain [1]. Recent evidence shows that α-syn proteinopathy can be induced in the periphery and spread into the brain via a prion-like transmission process [2,3,4]. How such proteinopathy can be transmitted from the periphery to the brain remains under investigation. Several reports have indicated that inter-neuronal connectivity underlies this transmission process [5,6]. The immune system is also thought to influence the prionoid transmission process of α-syn, though its role remains largely undefined [7,8].

The activation of immunity has long been implicated in synucleinopathies, especially Parkinson’s disease (PD) and multiple system atrophy (MSA) [9,10,11,12]. In PD, which is a disease characterized by the accumulation of aggregated α-syn in the brain and nigrostriatal degeneration, several seminal reports have characterized T-cell subsets in patients that are reactive to α-syn [13,14,15]. Rodent studies have shown somewhat disparate results, with one group observing that T cells increase pathogenicity, whereas others reported adoptive T cells as having a protective function [16,17]. Peripheral metabolic and immune homeostasis, as defined by the gut microbiome and blood monocyte phenotype, has also been reported to be altered in PD patients [18,19,20]. PET imaging in PD patients with dementia has shown activated microgliosis in the brain [21,22]. Collectively, this evidence shows that immune activation, whether peripheral or cerebral, is an integral part of disease pathogenesis in PD.

The identification of common genetic risk factors between PD and autoimmune disorders strongly suggests an etiologic link between the two. Genetic variants in the *HLA*-*DRB5* and *HLA*-*DQB1* loci increase the risk for PD as well as autoimmune diseases such as rheumatoid arthritis, ulcerative colitis and Crohn’s disease [23,24]. The *HLA* (human leukocyte antigen gene complex) loci code for major histocompatibility complex class II (*MHCII*) genes and is normally expressed in professional antigen-presenting cells (APCs), such as B cells and T cells. To understand the relationship between the *MhcII* gene function on these classical APCs and increased risk for PD, we crossed M83+/− transgenic mice that express the human A53T mutant *SNCA* (α-syn) [25] with a mouse model lacking the *MhcII* locus [26] (JAX stock #003584). M83+/− mice with partial or complete deletion in the *MhcII* locus were aged to 2 months and injected bilaterally in the hindlimb muscle with pre-formed α-syn pre-formed fibrils (PFFs). Following the intramuscular (IM) injection of PFFs, these mice were allowed to age for 2 months (‘prodromal stage’ group) or until they developed complete hindlimb paralysis ~4 months post PFF injection (‘endstage’ group) due to the accumulation of α-syn deposits and motoneuron degeneration, as we have shown earlier [27,28]. In the PFF-seeded M83+/− mice, we observed that the complete deletion of the *MhcII* locus (M83+/−, MHCII−/−; hereafter referred to as *MhcII*−/−) resulted in decreased lifespans compared to M83+/− mice with the wild-type *MhcII* allele (referred to as *MhcII*+/+ mice). We observed a higher burden of α-syn pathology and neurotoxic astrocyte signatures in *MhcII*−/− mice prior to the onset of disease phenotype. However, concurrent experiments in *Rag1*−/− mice showed that the functional lack of mature B/T cells does not influence the lifespan of mice injected with α-syn PFFs. Collectively, our data show that the loss of MhcII leads to dysfunctional responses in CNS-resident microglia and astrocytes to α-syn prion seeds.

## 2. Results

### 2.1. Absence of MhcII Reduces Lifespan in α-Syn PFF-Seeded M83+/− Mice

**Figure 1:**
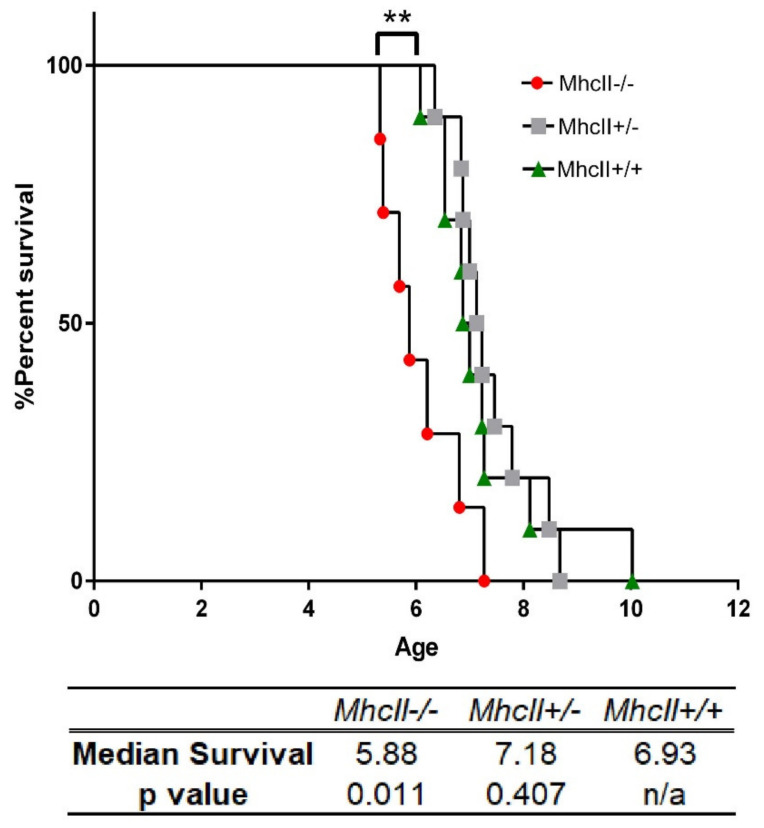
Deficiency in *MhcII* genes accelerates death in PFF-seeded M83 model of synucleinopathy. M83+/− mice with intact *MhcII* alleles (*MhcII*+/+) or lacking one (*MhcII*+/−) or both *MhcII* alleles (*MhcII*−/−) were seeded with α-syn PFFs at 2 months of age. Median survival times are indicated in months. *p*-values indicate comparison of all groups to *MhcII*+/+ mice. Gehan-Breslow-Wilcoxon test and Log rank test; ** *p* < 0.01. N = 10 (*MhcII*+/− and *MhcII*+/+) and 7 (*MhcII*−/−) mice.

In previous studies, we have shown that following the intramuscular (IM) injection of α-syn PFFs, pathological α-syn can progressively spread along the spinal cord into the brains of a human A53T α-syn transgenic (Line M83+/−) mouse model [27,28,29]. The role of cellular immunity in this peripheral to cerebral transmission of α-syn prion seeds remains under much debate. To characterize how the adaptive immune system regulates α-syn pathogenesis, we created M83+/− mice lacking the *MhcII* locus. At 2 months of age, these M83+/− mice were seeded with α-syn PFF and allowed to proceed to endstage, i.e., hindlimb paralysis. We observed that M83+/− mice lacking *MhcII*−/− (referred to henceforth as *MhcII*−/−) showed accelerated time to paralysis compared to M83+/− mice bearing endogenous *MhcII* alleles (referred to henceforth as *MhcII*+/+) (median survival times: MhcII−/−, 5.88 months; MhcII+/+, 6.9 months; *p* < 0.01) (Figure 1). M83+/− mice with the partial deletion of *MhcII*+/− did not show altered lifespans compared to *MhcII*+/+ mice (Figure 1; median survival age: *MhcII*+/−, 7.18 months). We tested whether this was accompanied by concomitant reduction in neurons in the nigrostriatal pathway, which is a cardinal feature in PD. Comparing *MhcII*+/+ mice with *MhcII*−/− mice, we found no change in the biochemical levels of tyrosine hydroxylase (TH) and DARPP32, two commonly used neuronal markers of this pathway (Supplementary Figure S1a–c). This suggests that, at least in this model of α-syn transmission, *MhcII* deficiency does not influence nigrostriatal neuron survival.

### 2.2. Accelerated Seeding of α-Syn Pathology in PFF-Injected MhcII−/− Mice

We wanted to examine whether the reduced lifespan in the α-syn PFF-seeded *MhcII*−/− mice was related to the burden of PFF-seeded α-syn pathology. We have shown earlier that following PFF seeding, α-syn pathology appears in a stereological fashion, spreading along the spinal cord, brainstem, midbrain and thalamus axis in an age-progressive manner [27]. Using an antibody against α-syn phosphorylated at Serine 129 [30], first, we confirmed *MhcII*−/− mice do not accumulate 81A-immunopositive intraneuronal α-syn pathology in the absence of PFF seeding even at 17 months of age (Supplementary Figure S2). This shows that the simple absence of *MhcII* does not trigger α-syn pathologies in M83+/− mice. Combined with our previous observations that PBS-injected *MhcII*+/+ mice did not develop any paralysis phenotype or α-syn pathology during their lifetime [28], this shows that PFF injections directly underlie the α-syn pathology and paralysis in *MhcII*−/− mice. 

**Figure 2:**
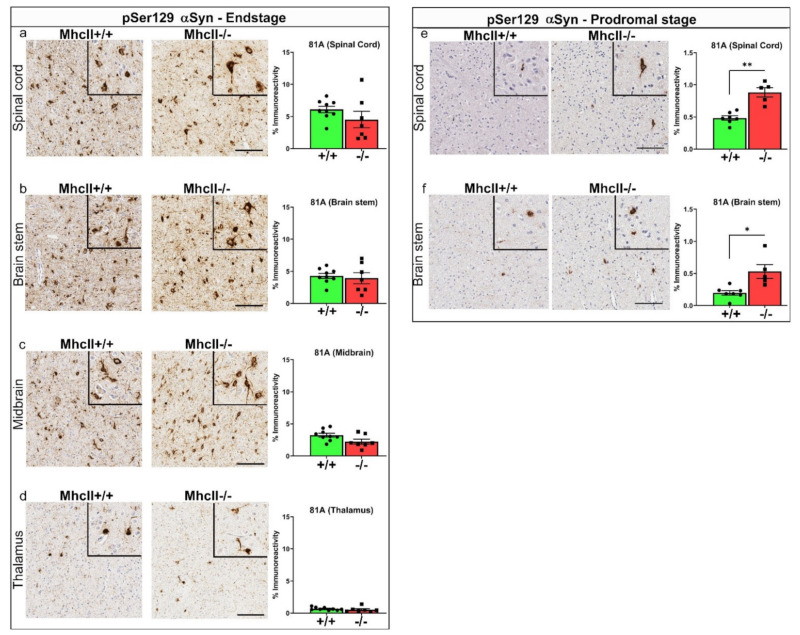
Earlier accumulation of pathologic α-syn in PFF-seeded *MhcII*−/− mice. *MhcII*+/+ or *MhcII*−/− mice were seeded with α-syn PFFs at 2 months of age and were analyzed at endstage (**a**–**d**) or at a prodromal stage 2 months post PFF seeding (**e**,**f**). Representative images and magnified detail (inset) of anti-pSer129-α-syn antibody 81A stained tissue sections from spinal cord, brain stem, midbrain and thalamus shown. Scale bar, 100 µm; inset, 50 µm. n = 7–8 mice/group (**a**–**d**) and n = 5–6 mice/group (**e**,**f**). Unpaired 2-tailed *t* test. * *p* < 0.05; ** *p* < 0.01.

We then investigated pathological α-syn burden in PFF-seeded mice that succumbed to paralysis at endstage. At endstage (analyzed ~4–5 months post PFF seeding), 81A-immunoreactive pSer129 α-syn pathology levels in the spinal cord and brain were similar between *MhcII*+/+ and *MhcII*−/− mice (Figure 2a–d). In prodromal mice (analyzed 2 months post PFF seeding), we found that the *MhcII*−/− mice accumulated higher 81A-reactive α-syn pathology burden in the spinal cord (*p* < 0.01) and brain stem (*p* < 0.05) (Figure 2e,f), suggesting the accelerated induction of seeding. At this time, we did not observe any detectable 81A immunostaining in the midbrain and thalamus areas of PFF-seeded *MhcII*+/+ and *MhcII*−/− mice (data not shown), consistent with the timeline of stereotypical progression shown earlier [27]. 

### 2.3. Reduced p62 Accumulation in PFF-Seeded MhcII−/− Mice

**Figure 3:**
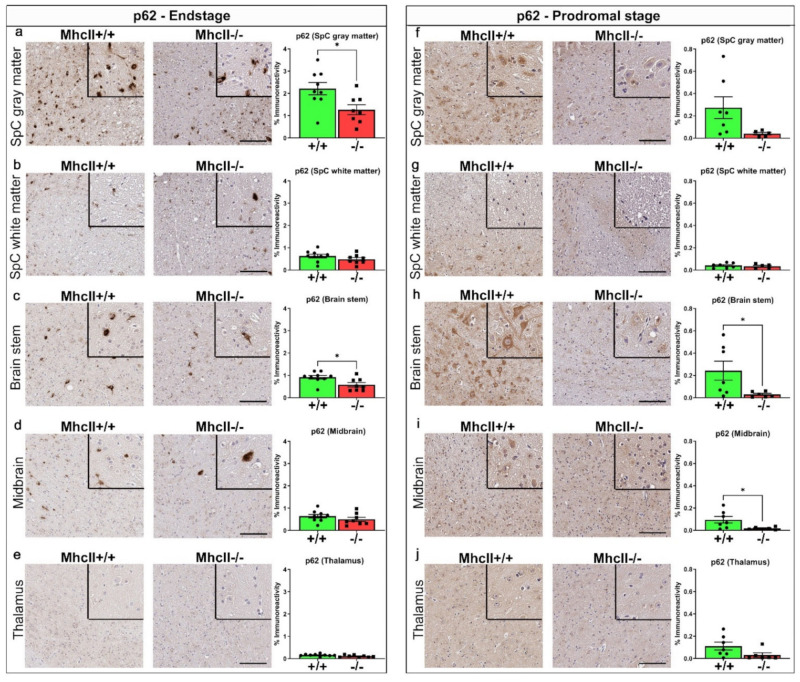
Reduced p62 levels in PFF-seeded *MhcII*−/− mice. *MhcII*+/+ or *MhcII*−/− mice were seeded with α-syn PFFs at 2 months of age and were analyzed at endstage (**a**–**e**) or at a prodromal stage 2 months post PFF seeding (**f**–**j**). Representative images and magnified detail (inset) of anti-p62 antibody-stained tissue sections from spinal cord (gray and white matter), brain stem, midbrain and thalamus shown. SpC, spinal cord. Scale bar, 100 µm; inset, 50 µm. n = 7–8 mice/group (**a**–**e**) and n = 5–6 mice/group (**f**–**j**). Unpaired 2-tailed *t* test. * *p* < 0.05.

Sequestome/p62, an autophagy-related protein, is associated with inclusion pathology [31]. Thus, we expected p62 levels to be consistent with the pathological burden of 81A immunostaining in PFF-seeded cohorts [27,32]. Surprisingly, we observed significantly reduced p62 levels in the spinal cord (*p* < 0.01 in gray matter compared to *MhcII*+/+ mice), and brainstem (*p* < 0.05 compared to *MhcII*+/+ mice) of endstage *MhcII*−/− mice (Figure 3a–e). This trend was also observed in the prodromal cohort of *MhcII*−/− mice (*p* = 0.074 in gray matter of spinal cord and *p* < 0.05 in brainstem and midbrain relative to *MhcII*+/+ mice) (Figure 3f–j). p62 levels were similar in the white matter of the spinal cord, midbrain and thalamus of endstage mice (Figure 3b,d,e) or in the white matter of the spinal cord and thalamus of prodromal cohorts (Figure 3g,j). 

The divergent observations of p62 and α-syn burden prompted us to consider the deregulation of autophagy processes as a factor underlying seeding outcome in *MhcII*−/− mice [33]. To additionally confirm whether the reduction in p62 is related to altered protein clearance via autophagy [34], we investigated LC3B levels and ubiquitin levels. LC3B regulates the cell-to-cell transmission of α-syn [35], a paradigm that we are modeling in our PFF-seeded mice. However, we did not observe any changes in LC3B in endstage mice (Supplementary Figure S3a–d), while LC3B was undetectable in prodromal mice (data not shown). Because ubiquitination is a key intermediate step in p62-mediated autophagy [36], we tested if there were any changes in global ubiquitination patterns (Figure S3e–h). We observed similar ubiquitin levels in PFF-seeded *MhcII*−/− mice compared to *MhcII*+/+ mice at endstage. Together, our data did not indicate autophagy as a major pathway that is altered in *MhcII*−/− mice following PFF seeding. 

### 2.4. Immunohistochemical Characterization of T Cells in PFF-Seeded Mice

**Figure 4:**
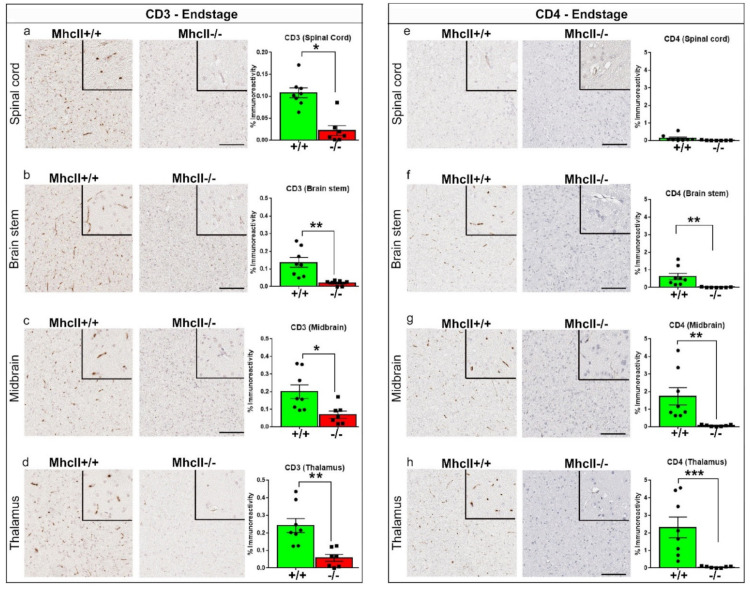
Reduced CD3+ T cell in endstage PFF-seeded *MhcII*−/− mice. *MhcII*+/+ or *MhcII*−/− mice were seeded with α-syn PFFs at 2 months of age and were analyzed at endstage (CD3, (**a**–**d**); CD4, (**e**–**h**)). Representative images and magnified detail of anti-CD3 and anti-CD4 antibody-stained tissue sections from spinal cord, brain stem, midbrain and thalamus shown. Scale bar, 100 µm; inset, 50 µm. n = 6–8 mice/group. Unpaired 2-tailed *t* test. * *p* < 0.05; ** *p* < 0.01; *** *p* < 0.001.

As T cells are potentially related to disease progression in PD [14] and MhcII is required for T cell’s function in antigen presentation, we were curious as to whether T cells are altered in a disease-stage-specific or area-specific manner in the PFF-seeded mice. We first used CD3 antibody to examine total circulating T cells in the spinal cord and brain of our experimental cohorts. CD3+ immunoreactivity was below detection levels in PFF-seeded *MhcII*+/+ and *MhcII*−/− mice in the prodromal stage (Supplementary Figure S4a–d). At endstage, we found robustly detectable CD3 staining in all areas of *MhcII*+/+ mice (Figure 4). Compared to prodromal-stage PFF-seeded *MhcII*+/+ mice, CD3 levels were increased in endstage PFF-seeded *MhcII*+/+ mice (midbrain: *p* < 0.05; thalamus: *p* < 0.01) (Figure 4a–d vs. Supplementary Figure S4a–d), showing T-cell infiltration in the CNS accompanies advancing disease. The CD3 immunoreactivity signal was predominantly associated with vascular morphology. Interestingly, CD3+ T cell burden was substantially lower in the *MhcII*−/− mice relative to *MhcII*+/+ mice (Figure 4a–d; *p* < 0.05 in spinal cord and midbrain, *p* < 0.01 in brainstem and thalamus). 

The *MhcII*−/− mice have been reported to be deficient in CD4+ T cells [26]. Consistent with this, CD4+ immunoreactivity was absent in *MhcII*−/− mice at all stages (Figure 4; Supplementary Figure S4), but detectable in *MhcII*+/+ mice at endstage (Figure 4e–h). Unlike CD3, CD4 staining was detectable in both prodromal and endstages in Mhc+/+ mice and was upregulated in endstage *MhcII*+/+ mice (midbrain: *p* < 0.05; thalamus: *p* < 0.001) relative to prodromal-stage *MhcII*+/+ mice in the midbrain (*p* < 0.05) and thalamus (*p* < 0.001) (Figure 4g,h vs. Supplementary Figure S4g,h). 

### 2.5. PFF Injection in Rag1−/− Mice Does Not Alter Survival or Endstage α-Syn Inclusion Pathology

**Figure 5:**
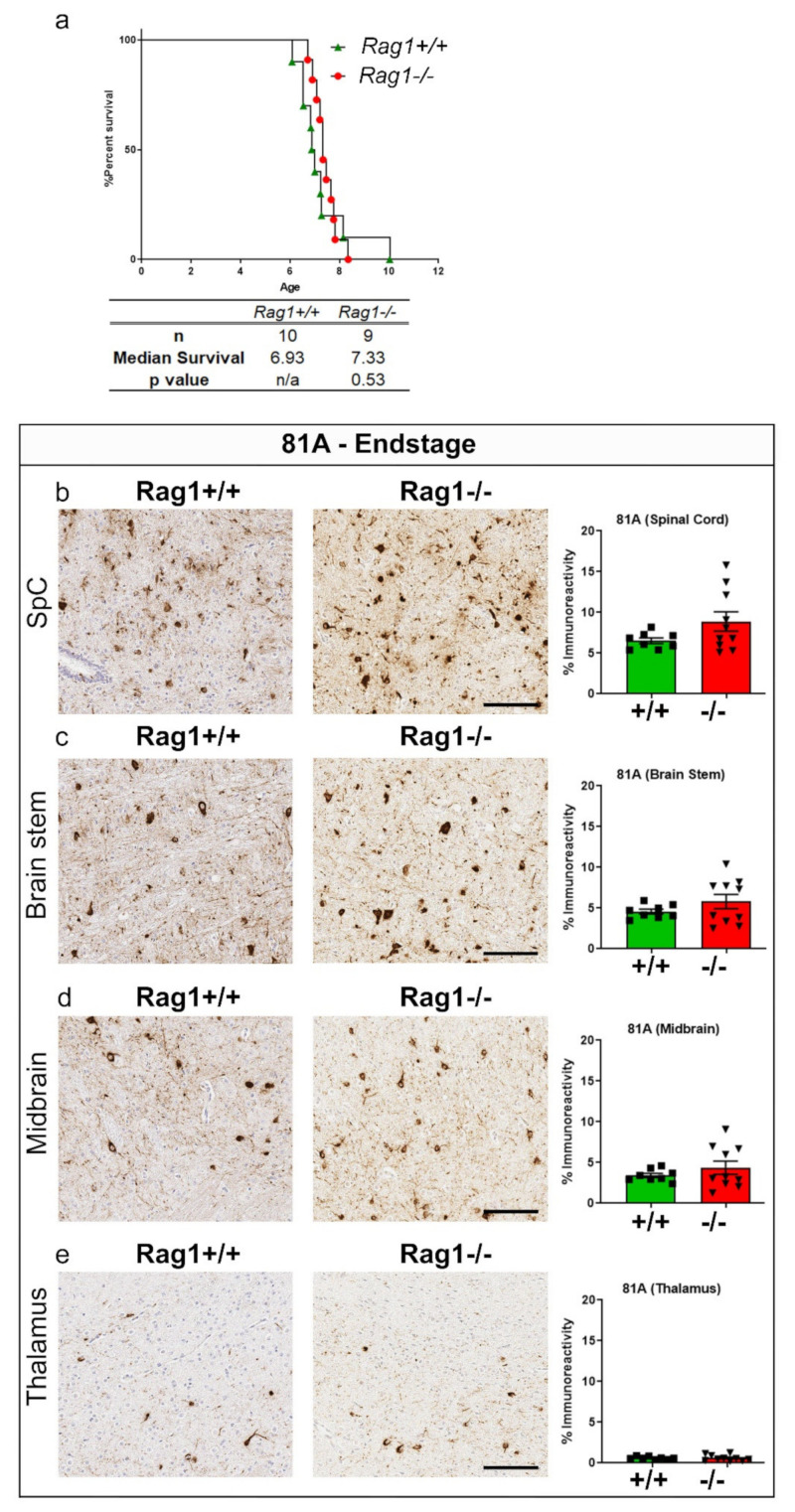
*Rag1* deficiency does not influence lifespan in PFF-seeded M83 model of synucleinopathy. M83+/− mice with intact *Rag1* (*Rag1*+/+) or lacking both Rag1 alleles (*Rag1*−/−) were seeded with α-syn PFFs at 2 months of age. Survival curve and median survival times are indicated in months (**a**). *p*-values indicate comparison of all groups to *Rag1*+/+ mice. Gehan-Breslow-Wilcoxon test and Log rank test N = 10 (*Rag1*+/+) and 9 (*Rag1*−/−) mice. Representative images and of anti-pSer129-α-syn antibody 81A stained tissue sections from spinal cord, brain stem, midbrain and thalamus of PFF-seeded mice at endstage (**b**–**e**). Scale bar, 100 µm; inset, 50 µm. n = 8–10 mice/genotype. Unpaired 2-tailed *t* test.

Several recent reports have shown that T-cell and B-cell homeostasis is influenced by synuclein [37]. Both B cells and T cells employ MhcII to conduct their basic function as individual and collaborative professional APCs [38]. To understand whether MhcII-mediated function on B and T cells leads to increased synuclein seeding and earlier death as we have observed in our PFF seeding model, we used the *Rag1*−/− mice. These mice in the homozygous condition lack all mature lymphocytes because Rag1 deficiency leads to the early developmental stage arrest of B cells and T cells [39]. We observed that the complete absence of Rag1 does not alter time to paralysis in PFF-seeded mice (Figure 5a). The immunohistochemical analysis of pSer129 α-syn in these mice at endstage did not reveal any differential burden of α-syn inclusion pathology (Figure 5b–e). This prompted us to investigate whether other immune cells (macrophages, microglia and astrocytes) are affected in PFF-seeded *MhcII*−/− mice. 

### 2.6. Modest Changes in Macrophages in PFF-Seeded MhcII−/− Mice

We used the macrophage marker, CD169, to deduce whether macrophages are involved in the immune response in PFF-seeded mice. We used the CD169 antibody as this molecule is specific to macrophages and CD169+ macrophages are considered as gatekeepers or the first line of defense in pathogen-initiated immunity [40]. CD169+ immunostaining was only observed in the midbrain and thalamus areas of endstage mice. CD169 immunoreactivity was reduced in the thalamus of PFF-seeded *MhcII*−/− mice relative to *MhcII*+/+ mice (Supplementary Figure S5a,b, *p* < 0.05). CD169 was not detectable in the spinal cord or brainstem of these mice (data not shown).

### 2.7. Increased Microgliosis in PFF-Seeded Endstage MhcII−/− Mice

The upregulation of microgliosis is a neuropathologic feature of α-syn prionoid transmission, as α-syn has been shown to be released from neurons and interact with microglial receptors [41]. We used three distinct microglial markers in our study: Iba-1, a generalized marker of microglia and macrophages; Tmem119, a marker of homeostatic microglia; and cd68, an activation marker of microglia and macrophages.

**Figure 6:**
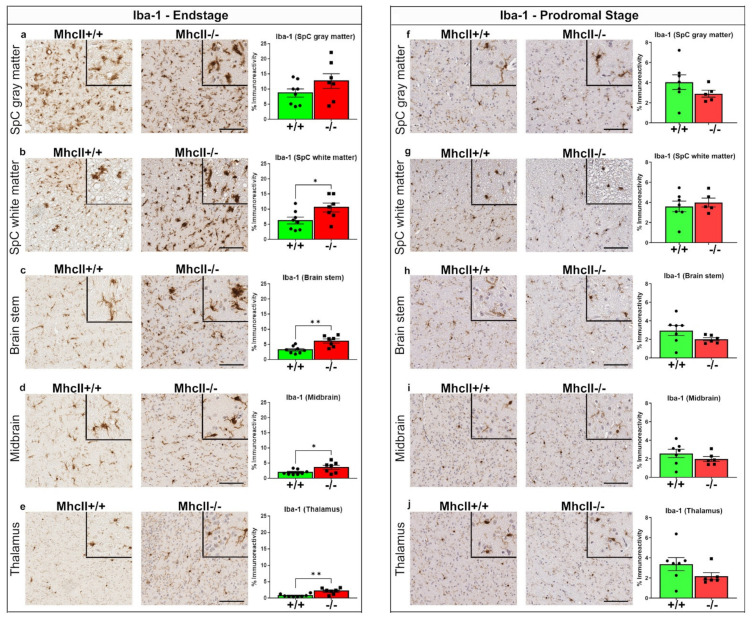
Increased microgliosis in PFF-seeded *MhcII*−/− mice at endstage. *MhcII*+/+ or *MhcII*−/− mice were seeded with α-syn PFFs at 2 months of age and were analyzed at endstage (**a**–**e**) or at a prodromal stage 2 months post PFF seeding (**f**–**j**). Representative images and magnified detail (inset) of anti-Iba-1 antibody-stained tissue sections from spinal cord (gray and white matter), brain stem, midbrain and thalamus shown. SpC, spinal cord. Scale bar, 100 µm; inset, 50 µm. n = 7–8 mice/group (**a**–**e**) and n = 5–6 mice/group (**f**–**j**). Unpaired 2-tailed *t* test. * *p* < 0.05; ** *p* < 0.01.

In the PFF-seeded model used in our study, robust Iba-1-reactive microgliosis initiates around 3 months post PFF seeding and continues to dominate the entire neuraxis until paralysis [27]. In the current study, we observed significantly increased Iba-1 reactivity in the white matter of the spinal cord, brainstem, midbrain and thalamus of PFF-seeded *MhcII*−/− mice at paralysis (Figure 6a–e; *p* < 0.05 in spinal cord white matter and midbrain, *p* < 0.01 in brainstem and thalamus). We did not observe any Iba-1 upregulation in the gray matter of the spinal cord in these *MhcII*−/− mice relative to *MhcII*+/+ mice (Figure 6a). In the prodromal stage, Iba-1 levels remained unchanged in PFF-seeded *MhcII*−/− mice relative to *MhcII*+/+ mice (Figure 6f–j). Additionally, we did not observe any change in the levels of homeostatic microglia in PFF-seeded *MhcII*−/− mice relative to *MhcII*+/+ mice at either the endstage or the prodromal stage (Supplementary Figure S6a–j).

**Figure 7:**
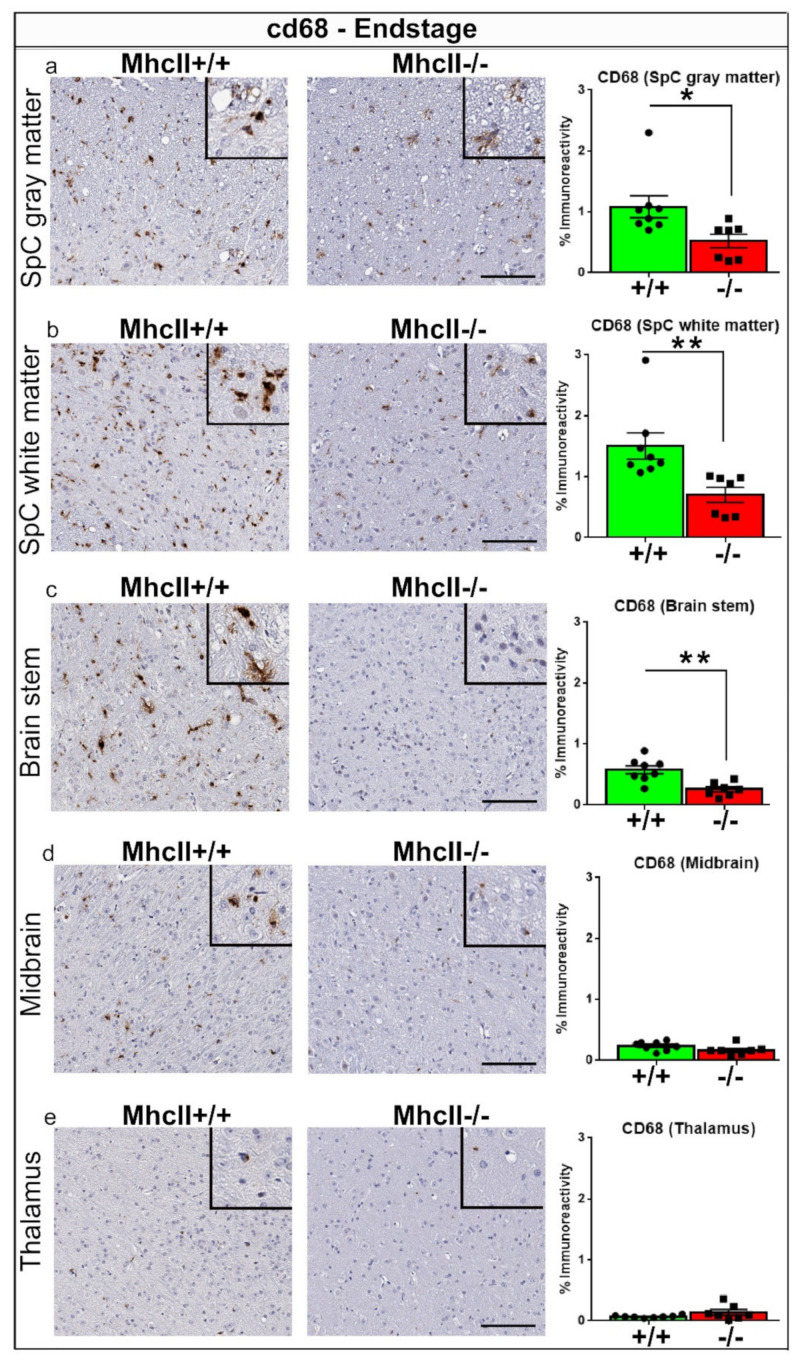
Reduced CD68 levels in PFF-seeded *MhcII*−/− mice at endstage. *MhcII*+/+ or *MhcII*−/− mice were seeded with α-syn PFFs at 2 months of age and were analyzed at endstage (**a**–**e**). Representative images and magnified detail (inset) of anti-CD68 antibody-stained tissue sections from spinal cord, brain stem, midbrain and thalamus shown. SpC, spinal cord. Scale bar, 100 µm; inset, 50 µm. n = 7–8 mice/group. Unpaired 2-tailed *t* test. * *p* < 0.05; ** *p* < 0.01.

Next, we used CD68, a lysosomal marker used for identifying activated microglia and macrophages. In the prodromal stage, there was no detectable CD68 immunoreactivity in PFF-seeded *MhcII*−/− or *MhcII*+/+ mice *I* (Supplementary Figure S5c). At endstage, we observed robust cd68 immunoreactivity in the spinal cord and brainstem and modest amounts in the midbrain and thalamus (Figure 7). This confirms that microglial CD68 is preferentially induced in the presence of α-syn inclusion pathology. Interestingly, the *MhcII*−/− mice displayed lower CD68 immunoreactivity (*p* < 0.05 in spinal cord white matter and brain stem, *p* < 0.05 in spinal cord gray matter) compared to *MhcII*+/+ mice, suggesting that *MhcII* deficiency reduces the activation level of microglia (Figure 7a–c). cd68 immunoreactivity was similar in the midbrain and thalamus of *MhcII*−/− and *MhcII*+/+ mice.

### 2.8. Astrocyte Burden Is Lower in PFF-Seeded MhcII−/− Mice 

**Figure 8:**
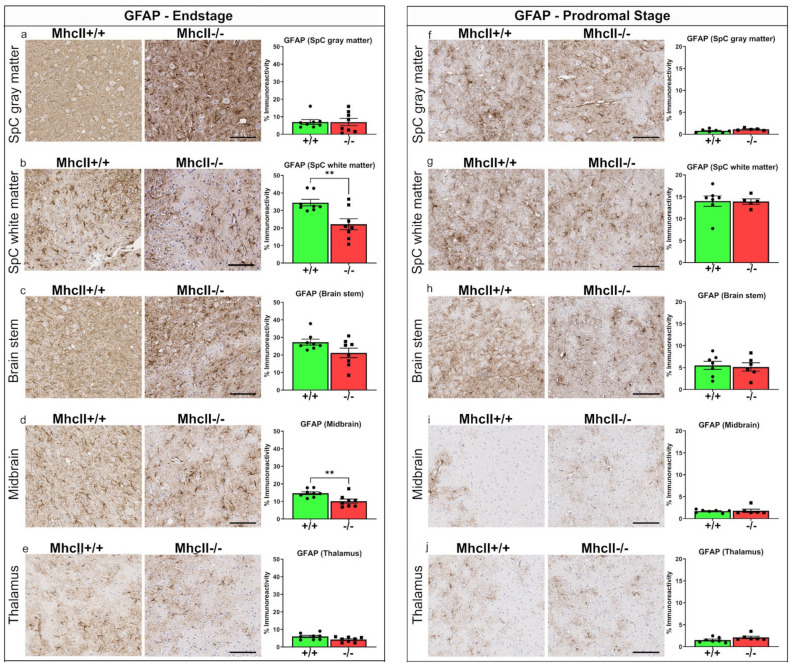
Astrocytosis is blunted in PFF-seeded *MhcII*−/− mice at endstage. *MhcII*+/+ or *MhcII*−/− mice were seeded with α-syn PFFs at 2 months of age and were analyzed at endstage (**a**–**e**) or at a prodromal stage 2 months post PFF seeding (**f**–**j**). Representative images and magnified detail (inset) of anti-GFAP antibody-stained tissue sections from spinal cord (gray and white matter), brain stem, midbrain and thalamus shown. SpC, spinal cord. Scale bar, 100 µm; inset, 50 µm. n = 7–8 mice/group (**a**–**e**) and n = 5–6 mice/group (**f**–**j**). Unpaired 2-tailed *t* test. ** *p* < 0.01.

Astrocytes are major cell types involved in synucleinopathies [42]. We have reported that PFF-seeded M83+/− mice show increased glial fibrillary acidic protein (GFAP) immunoreactivity in the spinal cord and brainstem at 3 months post injection [27]. Interestingly, the PFF-seeded endstage *MhcII*−/− mice showed reduced GFAP immunoreactivity compared to age-matched *MhcII*+/+ mice in the white matter of the spinal cord (*p* < 0.01) and midbrain areas (*p* < 0.01) (Figure 8b,d). No significant differences were noted in other areas examined, such as gray matter of the spinal cord, brainstem and thalamus (Figure 8a,c,e). In the prodromal stage, there was no difference in astrocyte staining in the PFF-seeded *MhcII*−/− mice relative to *MhcII*+/+ mice (Figure 8f–j). 

### 2.9. Focused Transcriptome Analysis Reveals Disease-Stage-Specific Gene Expression Signatures in PFF-Seeded Mice

**Figure 9:**
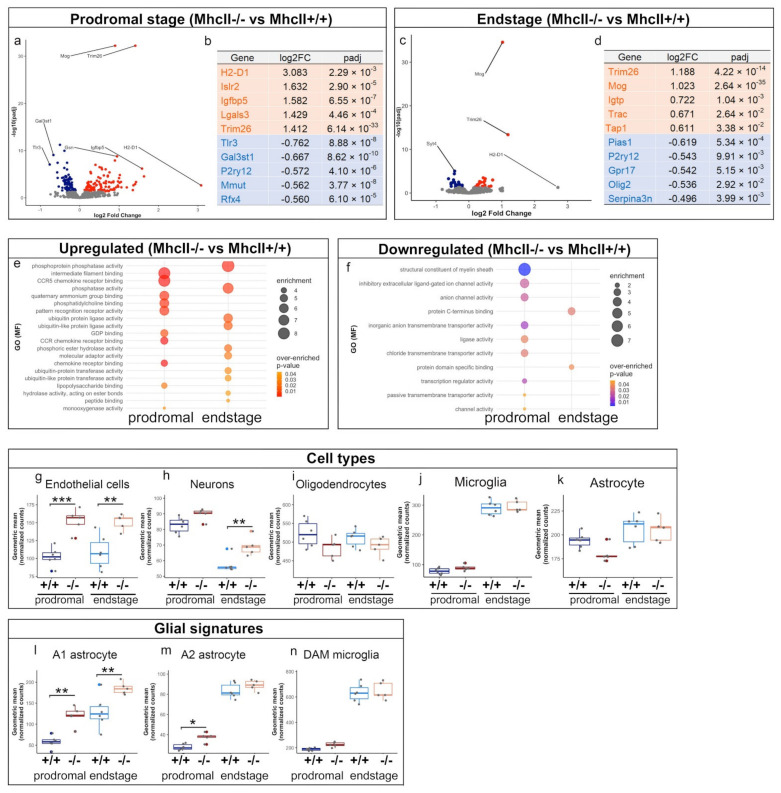
Focused transcriptomic analysis reveals altered immune function in PFF-seeded *MhcII*−/− mice. Differential gene expression was analyzed using NanoString Glial Profiling Panel in α-syn PFF-seeded *MhcII*+/+ or *MhcII*−/− mice. Mice were analyzed at endstage (paralysis) or at a prodromal stage (2 months post PFF seeding). Volcano plot and top 10 altered genes shown for prodromal cohort (**a**,**b**) and endstage cohort (**c**,**d**). Orange dots, upregulated genes; blue dots, downregulated genes. FC = fold change; padj = *p*-value adjusted for multiple comparison. The list of differentially expressed genes was used to impute enrichment of functional pathways (Gene Ontogeny Molecular Function, GO MF) among the upregulated genes (**e**) and downregulated genes (**f**) in bubble plots. Over-represented pathways with *p*-value ≤ 0.01, the number of module genes within the pathway > 5 and an enrichment score > 2.0 are depicted. *p*-value is indicated by the color score and the enrichment score by the dot size. Differential gene expression patterns of cell-type-specific markers in different cell types were imputed (**g**–**k**). Disease-associated glial signatures were identified in the prodromal and endstage mice (**l**–**n**). One-way Anova with Tukey’s correction. *** *p* < 0.001; ** *p* < 0.01; * *p* < 0.05. N = 5–6 mice/group.

We analyzed immune-specific gene expression changes in the endstage and prodromal PFF-seeded *MhcII*−/− mice and *MhcII*+/+ mice using the 770-gene NanoString Glial Profiling panel (Figure 9, Supplementary Tables S3 and S4). This panel is designed to highlight the neurodegeneration-related immune profile observed in humans and corresponding mouse models [43]. At endstage, 24 genes were upregulated and 28 were downregulated in the PFF-seeded *MhcII*−/− relative to *MhcII*+/+ mice (Supplementary Table S3). In the prodromal stage, 103 genes were upregulated and 98 were downregulated in the *MhcII*−/− vs. *MhcII*+/+ mice (Supplementary Table S4). We found that the genes that were induced in the prodromal and endstage of these mice were indicative of distinctive pathways (Figure 9a–f). Two genes that were significantly upregulated in both cohorts were Trim26 and Mog (Figure 9a,c). Trim26 (Tripartite motif-containing protein 26) is involved in protein ubiquitination and proteosomal degradation. It is regulated by CIITA (a regulator of *MhcII* genes) which plays a key role in antigen presentation [44] and anti-viral innate immune activation [45]. Mog (myelin oligodendrocyte glycoprotein) functions in maintaining the myelin sheath and is an important contributor to autoimmune diseases. Both Mog and Trim26 are located in a sub-telomeric region within the *MhcI* locus. Another *MhcI* locus gene, H2-D1, which is involved in antigen processing via MHC class Ib, is also upregulated in *MhcII*−/− mice, significantly in the prodromal cohort (Figure 9a,b) but marginally at endstage (Figure 9c,d). This is suggestive of increased MhcI-related autophagic processing in *MhcII*−/− mice. Supporting this observation, ubiquitination ligase, transferase, molecular adaptor activity, hydrolase and phosphatase-activity-associated GO pathways were enriched in both the prodromal and endstage cohorts of *MhcII*−/− mice (Figure 9e). Immune activation, as reflected by chemokine receptor, pattern recognition receptor and lipopolysaccharide-binding activities, were upregulated in the prodromal cohorts but not in the endstage cohort (Figure 9e). Among the downregulated GO pathways in *MhcII*−/− mice were those related to transcriptional control, such as C terminus binding and transcription regulation (Figure 9f). Astrocyte-function-related channel and transporter activities were additionally downregulated in the prodromal cohort of *MhcII*−/− mice (Figure 9f).

We next identified the cell types that were most perturbed in PFF-seeded *MhcII*−/− mice relative to *MhcII*+/+ mice. This classification is a facile representation of the extent of gene expression changes in cell-type-specific marker genes contained in bulk-analyzed tissue and does not imply any causation in disease progression. The endothelial gene expression signature was upregulated in prodromal and endstage *MhcII*−/− mice, while the neuronal signature was upregulated only in endstage mice, presumably in response to neurodegeneration (Figure 9g,h). We did not observe any changes in the oligodendrocyte or microglial gene expression levels in these cohorts (Figure 9i,j). Astrocyte gene expression patterns were marginally altered in the prodromal stage only (Figure 9k).

Several neurodegenerative gene expression signatures have been identified in AD and related dementias. Among these is the astrocyte-specific A1/A2 signature that is related to the expression of inflammatory cytokines [46] and microglia-specific DAM and MGnD signatures that are related to Trem2/Apoe function [47,48]. We found that neurotoxic A1 astrocytes were upregulated in both prodromal and endstage *MhcII*−/− mice relative to *MhcII*+/+ mice (Figure 9l), and the neuroprotective A2 astrocyte signature was only upregulated in the prodromal *MhcII*−/− mice (Figure 9m). This suggests that astrocytes play a key role in the progressive neurodegenerative pathology accompanying α-syn pathogenesis in an MhcII-deficient milieu. There was no change in DAM microglia profile (Figure 9n) or MGnD profile (data not shown) between the PFF-seeded *MhcII*−/− mice relative to *MhcII*+/+ mice at either the prodromal or endstage. 

## 3. Discussion

**Figure 10:**
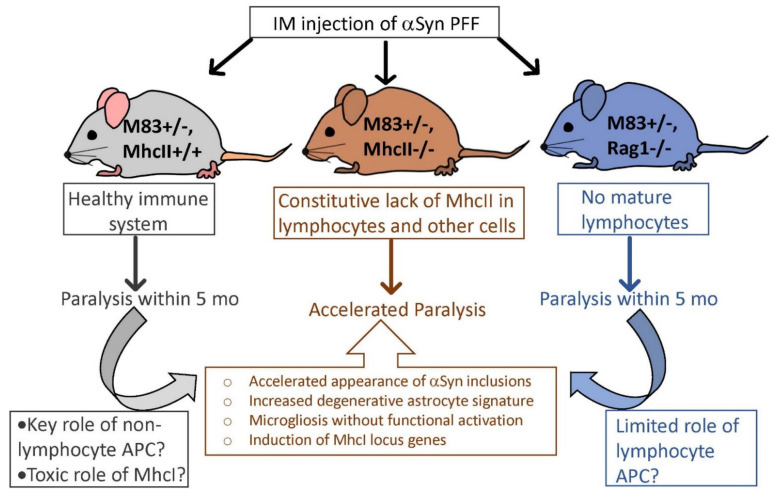
Schematic depiction of immune-mediated pathways influencing peripheral to central progression of synucleinopathy. Schematic summary of the main outcomes of our data pointing to a potential key role of MhcII activity in regulating disease progression of synucleinopathies. Increased astrocytosis and microgliosis accompanied worsened lifespan in α-Syn PFF-seeded mice constitutively lacking *MhcII* but not in mice lacking functional T cells or B cells, supporting a key role for non-lymphocytic antigen presentation activity in this process. Increased expression of *MhcI* locus genes in α-Syn PFF-seeded *MhcII*−/− mice also indicates its potential involvement in α-Syn pathogenesis.

Recent evidence strongly suggests that synucleinopathies, such as PD, are regulated by both innate and adaptive immune systems [16]. Based on genetic data that variants in the *HLA* allele increase the risk of PD [49,50] and MhcII is upregulated in rodent models of synucleinopathies [51,52], we investigated how deleting the *MhcII* genes would affect the peripheral to cerebral transmission of α-syn prion seeds (Figure 10). We report that (1) deficiency in the *MhcII* locus accelerates α-syn inclusion pathology and time to paralysis following peripheral PFF seeding; (2) complete deficiency in mature lymphocytes does not influence the transmission rates of PFF-seeded α-syn pathology or survival; (3) a deficiency in the *MhcII* locus is associated with astrocyte dysfunction evidenced by a reduced number of astrocytes and the induction of the neurodegeneration-associated A1 signature; (4) *MhcII* locus deficiency increases microglial burden, but the microglial phagocytosis marker is reduced; (5) gene expression analysis reveals the induction of *MhcI* locus genes related to antigen presentation and anti-viral response. Overall, we show that a deficiency in the *MhcII* locus induces selective *MhcI* locus gene expression and dysfunctional microglial and astrocytic response to α-syn pathology, accelerating the paralysis phenotype accompanying the peripheral to cerebral transmission of α-syn pathology. Our study is consistent with a recent study that intracerebral PFF seeding is aggravated in immunocompromised NOD scid gamma mice that lack T, B and Natural Killer cells [17]. Combined with independent observations that allelic variations in *MHCII* in rats result in differential susceptibility to α-syn-induced pathology [53,54], our study suggests that MhcII function is a key factor in the etiology of synucleinopathies.

To our knowledge, this is the first demonstration of how MhcII deficiency regulates the prionoid transmission of α-syn from the periphery into the brain. Our results are somewhat distinct from earlier studies where *MhcII* deficiency prevented dopaminergic neurodegeneration following the intra-nigral expression of AAV-α-syn [52]. One of the mechanisms in this hyperexpression α-syn model was identified to be the infiltration of Ifn-γ producing neurotoxic CD4+ lymphocytes [16]. This study further showed that CD4-deficient mice were protected from α-syn-induced dopaminergic cell loss. A related study from this group showed that the RNA silencing of the class II transactivator (CIITA), a transcriptional co-activator required for MHCII induction, phenocopies their observations from the *MhcII*-deficient model [55]. In our model, we also observed severely attenuated CD4+ T cells, which is an expected phenotype in the *MhcII* KO model [26], but in our study, this was correlated with the increased induction of peripheral to cerebral transmission of α-syn. The disparate results between these studies [52] and ours might simply be attributed to the location of the initial α-syn pathology. It is possible that because we administered the α-syn in the hindlimb muscle which is highly vascular and available to peripheral immune system, our model was influenced relatively directly by the antigen-presenting and macroautophagy properties (or lack thereof in the *MhcII*−/− model) of the professional immune cells, whereas the former study [52] recapitulated a paradigm for the recruitment of peripheral cells into the relatively immune-privileged brain in response to the hyperexpression of α-syn in the brain. Another reason could be simply attributable to the immunodeficient *MhcII* KO mice displaying distinct baseline immune phenotypes when maintained under different laboratory conditions, which has been elegantly demonstrated to occur in a mouse model of c9orf72 [56].

A recent study identified CD3+ T cells in close proximity to neurons bearing α-syn inclusions in LBD patients [15]. This study also identified discrete populations of α-syn reactive CD4+ T cells that express CXCR4 and IL17A which were present in the cerebro-spinal fluid of synucleinopathy patients and are correlated to the neurodegenerative phenotype [15]. In our study, we observed that both CD3+ and CD4+ immunoreactivity increased in the brains of *MhcII*+/+ mice, consistent with these human data. However, in the *MhcII*−/− mice that inherently lacked CD4+ cells, we found that this phenotype was associated with reduced lifespan. Thus, it would imply that in our model of the peripheral to cerebral transmission of α-syn, the relative lower abundance of CD4+/CD3+ T cells is correlated with the exacerbation of α-syn-induced motoneuron degeneration. Collectively, our data suggest that other cellular pathways may exist, in addition to CD3/CD4 signaling, which could trigger the neurodegenerative effect induced by peripheral to cerebral α-syn transmission. 

Our results reveal an intriguing relationship between the loss of MhcII function and the upregulation of the neurotoxic disease-associated A1 astrocyte signature. The A1 signature has been shown to be transcriptionally related to the chronic expression of Il-1α, TNF and C1q by reactive microglia [46,57]. This astrocyte signature has been shown to be associated with chronic microgliosis, which would be consistent with increased Iba-1 immunostaining that we observed in the PFF-seeded *MhcII*−/− mice. It is important to note here that though we observed higher Iba-1 immunostaining, the transcriptome analysis of PFF-seeded *MhcII*−/− mice did not reveal neurodegeneration-specific microglial phenotypes [58]. Notably, we also observed that the A2 astrocyte phenotype, characterized by S100a expression and related to cell repair, is also upregulated in the prodromal disease phase [46]. Indeed, in the prodromal disease stage in PFF-seeded *MhcII*−/− seeded mice, we found that both A1 and A2 signatures were upregulated. As mice aged and approached the endstage, the A1 phenotype predominated the immune landscape. Keeping in mind the fluidity of the A1/A2 classification [59], our data seem to indicate that *MhcII* deficiency is associated with increased inflammatory, and potentially neurodegenerative, astrocyte response to α-syn. It is notable that this neurodegenerative signature is not reflected in facile immunohistochemistry with anti-GFAP antibody, which showed reduced burden especially at tendstage. Given that the emerging literature suggests that astrocytes, in particular, are efficient APCs in the context of α-syn [60], the role of astrocytes and astrocytic MhcII in α-syn transmission will need further clarification.

Our gene expression data indicate an anti-viral response as shown by the upregulation of chemokine receptors and the downregulation of transcription in the prodromal phase in the PFF-seeded *MhcII*−/− mice. This is also exemplified by the upregulation of several genes in the *MhcI* locus (*Trim 21*, *Mog*, *H2*-*D1*) as MhcI is canonically induced as part of the anti-viral response [61]. The induction of *MhcI* genes in our model is intriguing, as this would imply some form of crosstalk between the antigen presentation function within the MhcI/II axis. Interestingly, previous studies have identified that α-syn can induce neuronal MhcI, leading to a neurodegenerative effect on midbrain neurons [62]. The induction of neuronal MhcI in this study [62] was related to the recruitment of CD8+ T lymphocytes, which have been shown to be a major pathway for neurodegeneration following viral infection [63]. Further studies in our transmission model could include establishing whether CD8+ lymphocytes play a mechanistic role in α-syn induced neurodegeneration. 

Our neuropathologic and gene expression data suggest that α-syn transmission in *MhcII*−/− mice is associated with the deregulation of autophagic responses. On the one hand, we saw the upregulation of *MhcI* genes which would be consistent with the increased processing of antigens, but neuropathologically, we observed reduced p62 levels. Previous data show that p62 facilitates the autophagic removal of α-syn and its deficiency exacerbates α-syn aggregation [64,65], which would be consistent with our data. We also observed reduced CD68, which would suggest reduced phagocytosis. Together, our data show that a deficiency in MhcII leads to the deregulation of intracellular trafficking and clearance pathways. 

In conclusion, we showed that a deficiency in *MhcII* gene activity, but not *Rag1*, leads to exacerbated α-syn seeding and transmission from the periphery into the brain (Figure 10). Our data indicate that microgliosis, the induction of neurotoxic astrocyte gene expression signature and the induction of *MhcI* genes underlie this neurodegenerative phenotype. Our study reflects the close association of MhcII-mediated immune function and intracellular proteinopathy machinery in regulating the etiology of synucleinopathies.

## 4. Methods and Materials

### 4.1. Mice

All experiments were approved by the University of Florida IACUC. Mice were fed ad libitum and maintained in an SPF facility on a 12 h light/dark cycle. For these experiments, M83+/+ mice [25] were mated with *MhcII*−/− mice (JAX stock #003584; [26]) or *Rag1*−/− mice (Jax stock # 002216; [39]). Mice that are deficient in the whole *MhcII* locus have dramatically attenuated CD4+ T cells which interferes with antigen presentation capability but are otherwise viable and fertile [26]. Mice that are deficient in Rag1 produce no mature T cells or B cells but are phenotypically indistinguishable from wild-type mice [39]. The resulting heterozygotes were back-crossed to parental *MhcII*−/− mice or *Rag1*−/− mice, and M83+/− mice that were heterozygotes or homozygotes for MhcII or Rag1 deficiency were selected for further experiments. Genotyping was conducted following Jax labs protocols. There was no premature death noticed in these mice. Endstage was characterized by complete hindlimb paralysis and an IACUC body condition score of 2+. The cohort sizes are for the PFF-seeded endstage group were: *MhcII*+/+ = 7M and 3F mice; *MhcII*−/− = 4M and 4F mice. The prodromal group of PFF-seeded mice was analyzed at 4 months of age (2 months post PFF injection). The cohort sizes were *MhcII*+/+ = 3M and 3F mice; *MhcII*−/− = 3M and 2F mice. An additional group of naïve (not injected with PFF) *MhcII*−/− mice was analyzed at 17 months (n = 3M and 2F). The cohort size for PFF-seeded *Rag1*−/− mice was 3M and 6F mice.

### 4.2. Generation of α-Syn PFF and Intramuscular Injection

Recombinant full-length mouse αSyn protein expressed in *E. coli* was purified and aggregated in sterile PBS (Invitrogen) via incubation at 37 °C with continuous shaking at 1050 rpm. We have shown earlier that recombinant αSyn prepared in our laboratory contains ~5EU/ml bacterial lipopolysaccharide carry-over using the HEK-Blue hTLR4 assay (Invivogen) [66]. Preformed fibrillar (PFF) αSyn was validated using K114 fluorometry and electron microscopy, as previously described [66,67,68] (Supplementary Figure S1d,e). These 1 mg/mL mouse α-syn PFFs were fragmented via water bath sonication for 1 h before intramuscular (IM) injection in mice. Two-month-old M83+/− mice were anesthetized with isoflurane. After shaving the back of the hindlimb, a 10 μL Hamilton syringe with a 27-gauge needle was inserted ~1 mm into the gastrocnemius muscle to deliver 5 µg of α-syn fibril or 5 µL of sterile PBS in each hindlimb, as described earlier [27]. The injection of α-syn PFFs in these M83+/− mice leads to the progressive induction of α-syn pathology in the neuraxis, motoneuron degeneration, neuroinflammation and paralysis by 4 months post injection [28]. PBS-injected or naïve, uninjected M83+/− mice did not show αSyn pathologies or phenotypes during their lifespan [25,28].

### 4.3. Mouse Tissue Harvesting

Mice were deeply anesthetized using carbon dioxide inhalation, euthanasia was performed using intracardiac perfusion with PBS containing heparin, and brains and spinal cords were harvested as described earlier [29]. Brains were sliced sagittally into two halves, with one half flash frozen while the other half was fixed in 10% normal buffered formalin and processed for paraffin embedding. The entire spinal cord was divided into 3 sections: the anterior portion containing the cervical and thoracic segments (12 mm tissue from the proximal section of the spinal cord); the middle portion containing part of the thoracic segment (4 mm tissue from midline); and the posterior portion containing the lumbar segment (12 mm tissue from distal section). The anterior and posterior portions were fixed in 10% normal buffered formalin, while the middle portion was flash frozen. Following paraffin processing, the anterior and posterior sections were serially dissected every ~4 mm into 2–3 coronal sections and mounted on a single paraffin block. The hemi brain (dissected at the midline ~0.5 mm from the midline) was fixed in 10% normal buffered formalin and embedded in the same block as the spinal cord sections. Thus, each slide contained 4–6 coronal sections representing the entire spinal cord and a sagittal section of the brain (at 0.6–0.96 mm lateral from the midline). Each paraffin block was serially sectioned at 5 µm thickness. Each antibody immunohistochemistry was conducted on numerically identical slides from different mice, thus ensuring as much as possible that slides were stained at similar anatomic levels across the cohorts.

### 4.4. Immunohistochemical Characterization and Data Analysis

Immunohistochemical staining was performed on formalin-fixed, paraffin-embedded sections as described earlier [29]. A list of primary antibodies used in this study is provided in Supplementary Table S1. After the slides were de-paraffinized in xylene and rehydrated in a series of graded ethanol, antigen retrieval was performed in an appropriate buffer solution (Table S1), followed by incubation in an appropriate secondary antibody (ImmPRESS Polymer Reagent, Vector Labs) for 30 min at room temperature. Color was developed using 3,3′-diaminobenzidine (DAB Peroxidase HRP Substrate Kit, Vector Labs) and counterstained using hematoxylin (Vector Labs). Finally, brain sections were dehydrated in a series of graded ethanol, cleared in xylene, and mounted using Cytoseal-60 media. 

Immunostained slide images were captured using the Scanscope XT image scanner (Leica Biosystems, Deer Park, IL, USA). One sagittal brain section accompanied by 4–6 coronal sections of the spinal cord were quantified for each stain. Initially, the brain and spinal cord regions were defined by Paxinos and Franklin’s Mouse Brain in Stereotaxic Coordinates brain atlas using hematoxylin staining, and slides containing anatomically similar regions across all samples were selected for immunohistochemistry. The immunostained brain and spinal cords were outlined into anatomical specific regions using Aperio ImageScope software (Leica Biosystems). Then, the percent immunoreactivity for each region was computed at equivalent bregma levels using the Aperio Positive Pixel Count program (Aperio, Vista, CA, USA). The Positive Pixel count program was configured to detect brown color (ImageScope, Aperio Technologies, Vista, CA, USA). The total number of brown pixels (positive) divided by the total number of all pixels (positive + negative) was represented as % immunoreactivity burden. For spinal cords, the % immunoreactivity values on all serial sections on each slide were counted for any given sample and averaged across the anterior and posterior spinal cord sections for each mouse. For brain sections, the different parts of the annotated brain were separately analyzed using the Positive Pixel Count program. 

### 4.5. Western Analysis

Frozen hemibrains were weighed and homogenized using Omni Tissue Master 125 Handheld Homogenizer with an appropriate volume of 1× RIPA buffer with Triton ×100 (Boston BioProduct, Milford, MA, USA) and a protease and phosphatase inhibitor cocktail (Pierce). The homogenate was cleared by centrifugation at 12,000 rpm for 30 min at 4 °C. Following protein quantification (Bicinchoninic Acid assay, Thermo Scientific, Waltham, MA, USA), 30 µg of protein was separated on 4–20% Tris-Glycine gel (Novex, Invitrogen, Thermo Scientific, Waltham, MA, USA). Protein samples were then transferred to the Immobilon-PSQ membrane (MilliporeSigma, St. Louis, MO, USA) and incubated with primary antibodies overnight at 4 °C (Table S1). Protein bands were visualized using the multiplex Li-Cor Odyssey Infrared Imaging system (Li-Cor Biosciences, Lincoln, NE, USA) and quantified using ImageJ 1.53k software (NIH, Bethesda, MD, USA). The results are presented as average relative band intensity (normalized to loading control) ± SEM.

### 4.6. RNA Preparation and NanoString Analysis of Gene Expression

RNA was extracted from frozen thoracic spinal cords using the column (Invitrogen) purification of Trizol homogenates. For the endstage group, all mice displayed complete bilateral paralysis, whereas for the prodromal group, all mice were asymptomatic and healthy. A total of 100 ng of the total RNA was analyzed on a commercially available NanoString Glial Profiling codeset (NanoString, Seattle, WA, USA). Raw count data were checked for data quality using nSolver version 4.0 (NanoString, Seattle, WA, USA) and imported into R version 3.6 and Bioconductor version 3.9 (https://bioconductor.org/news/bioc_3_9_release/, accessed on 15 June 2022). Count matrices were normalized, and differentially expressed genes were analyzed with DESeq2 version 1.24.0 (https://bioconductor.org/packages/release/bioc/html/DESeq2.html, accessed on 15 June 2022) using default normalization parameters and housekeeping genes included in the codeset. For gene ontology analysis, a false discovery rate (FDR) of less than 0.05 was used as a cutoff. Goseq version 1.36.0 (https://bioconductor.org/packages/release/bioc/html/goseq.html, accessed on 15 June 2022) was used for gene ontology analysis using the list of genes included on the NanoString codeset as a background for analysis. The final *p*-values reported were adjusted for multiple comparisons (Padj). Graphs were generated with ggplot2 version 3.2.0 (https://cran.r-project.org/web/packages/ggplot2/index.html, accessed on 15 June 2022). The significance of the association (*p*-value) was reported following Tukey’s correction. To calculate the cell-type-specific DEGs and neurodegeneration-associated glial gene expression signatures, the geometric means of a set of genes characterizing each profile were used, as described before [69]. Briefly, we used a transcriptomic database which compiled the expression from RNA sequencing from different cell types (neurons, astrocytes, microglia, newly formed oligodendrocytes, endothelial cells and pericytes) in the brain [70] to generate lists of genes whose expression was enriched in each representative cell type (Table S2). To calculate neurodegeneration-associated gene expression A1, A2, DAM and MGnD signatures, the gene list was derived from the original reports [46,47,71] (Table S2). Inner joins between these gene lists and the genes present in the NanoString codeset were used to further restrict analyses to just those genes whose expression was monitored in these experiments. For each individual sample, the geometric mean of the normalized counts was calculated, and cell type signatures were further evaluated as the mean for each experimental group. 

### 4.7. Statistics and Data Preparation

Data were analyzed as specified in the figure legends. All statistics and graphical representations were conducted and performed using Graph Pad Prism version 9. The final images were assembled using Adobe Photoshop.

## Data Availability

All data generated or analyzed during this study are included in this published article and its Supplementary Information Files.

## Supplementary Materials

The following supporting information can be downloaded at: www.mdpi.com/article/10.3390/ijms23158175/s1.

## Abbreviations

α-syn	α-synuclein
AAV	adeno-associated virus
DAM	damage-associated microglia
DEG	differential expression of genes
DLB	dementia with Lewy bodies
FDR	false discovery rate
HLA	human leukocyte antigen
IM	intramuscular
KO	knock-out
Mhc	major histocompatibility complex
Mog	myelin oligodendrocyte glycoprotein
MSA	multiple system atrophy
PD	Parkinson’s disease
PFF	pre-formed fibrils of α-syn
SpC	spinal cord
Trim 26	Tripartite motif-containing protein 26
WT	wild-type
