# Supplementary material for: MhcII Regulates Transmission of α-Synuclein-Seeded Pathology in Mice
Source: Int J Mol Sci. 2022 Jul 25;23(15):8175. doi: 10.3390/ijms23158175 (PMC9332117; doi:10.3390/ijms23158175)
Supplement: Supplementary file 1 [file ijms-23-08175-s001.zip › Revised_Supplementary Tables.pdf]

Supplementary Table S1: List of antibodies used in this study

Primary Antibody	Dilution	Retrieval	Secondary Antibody	Source	Catalogue #
**Immunohistochemistry**					
81A	1 : 10,000	Water	Mouse	Benoit Giasson	N/A
p62	1 : 1000	Water	Rabbit	Proteintech	18420-1-AP
LC3B	1 : 1000	Citrate, pH 6.0	Rabbit	Cell Signaling	3868S
Iba-1	1 : 1500	Citrate, pH 6.0	Rabbit	Wako	019-19741
CD68	1 : 2000	Citrate, pH 6.0	Rabbit	Invitrogen	PA5-78996
CD3	1 : 100	Tris EDTA, pH 9.0	Mouse	Abcam	ab5690
GFAP	1 : 4000	Water	Rabbit	DAKO	Z0334
TMEM119	1 : 2000	Citrate, pH 6.0	Rabbit	Synaptic Systems	400002
CD169	1 : 75	Citrate, pH 6.0	Mouse	Novus	NB600-534
CD4	1 : 500	Citrate, pH 6.0	Mouse	Abcam	ab183685
**Immunoblotting**					
Tyrosine Hydroxylase	1 : 500	-	Rabbit	Millipore	ab152
DARPP32	1 : 500	-	Rabbit	Cell Signaling	2306
Beta Actin (AC-15)	1 : 1000	-	Mouse	Sigma	A5441

Supplementary Table S2: List of genes used to impute cell type-specific DEG and glia-specific signature

Cell type markers	Gene names
Astrocyte	Adcyap1r1, Aldh1l1, Aqp4, Egfr, Entpd2, Gdpd2, Gria2, Il18, Itga7, Nwd1, Ptprz1, Ptx3, Rfx4, Sox9, Vegfa
Endothelial cell	Cd34, Cldn5, Emcn, Fcgrt, Flt1, H2-D1, Icam2, Lsr, Myct1, Nostrin, Ptgis, Sparc, Srgn, Tie1
Microglia	B2m, C1qa, C1qb, C1qc, Ccl12, Ccl2, Ccl3, Cd14, Csf1r, Cst3, Ctsb, Cx3cr1, Gpr84, Il1a, Il1b, Irf8, Itgam, Lag3, Lrrc25, Ncf1, Olfm3, P2ry12, P2ry13, P2ry6, Sirpa, Tgfb1, Tlr2, Tmem119, Tnf, Trem2, Tyrobp
Neuron	Cnr1, Cx3cl1, Dlx1, Dlx2, Gabrb3, Gabrg2, Gria1, Grm2, Islr2, Opcml, Slc17a6, Tbr1
Oligodendrocyte	Erbb3, Gjb1, Gpr17, Gsn, Klk6, Mbp, Mog, Myrf, Olig1, Plekhb1, Plxnb3, Prkcz, Slpi, Sox10

Glia signatures	Gene names
A1	Amigo2, Fbln5, Gbp2, Ggta1, H2-D1, H2-T23, Psmb8, Serping1, Srgn
A2	B3gnt5, Cd109, Cd14, Clcf1, Emp1, Ptgs2, Ptx3, S100a10, Slc10a6, Sphk1, Tgm1, Tm4sf1
DAM	Adamts1, Ank, Aplp2, Apoe, Axl, Bhlhe41, C3ar1, Ccl3, Ccl6, Cd14, Cd34, Cd68, Cd74, Cd9, Clec7a, Csf1, Cst7, Ctsb, Ctsd, Ctsl, Egr1, Fabp5, Fcgr1, Flt1, Fos, Gnas, Gpnmb, Grn, H2-T23, Hexb, Igf1, Il1b, Irf8, Itgax, Lamp2, Lpl, Lyz2, Mafb, Mertk, Myo5a, Nptn, Plxdc2, Pmp22, Rhob, Serpine2, Sirpa, Slco2b1, Spp1, Sqstm1, Syngr1, Timp2, Tlr2, Tnf, Trem2, Tyrobp, Vim, Ywhah, Gusb
MGnD	Apoe, Axl, Ccl2, Clec7a, Csf1, Cybb, Gas7, Gpnmb, Itgax, Lag3, Lgals3, Msr1, Spp1, Tlr2

Supplementary Table S3. Differential gene expression list of end stage α-Syn PFF seeded MhcII-/- vs MhcII+/+ mice. baseMean, mean transcript levels across all samples; log2FoldChange, log of fold change of RNA; lfcSE, standard error of log of fold change; stat, Wald statistics; padj, p value adjusted for multiple comparison.

	**baseMean**	**log2FoldChange**	**lfcSE**	**stat**	**pvalue**	**padj**
Mog	1874.4532	1.057308455	0.090642	11.66471	1.93E-31	1.34E-28
Trim26	122.48446	1.160070648	0.146991	7.892134	2.97E-15	1.03E-12
Hsp90ab1	2439.0195	0.430975236	0.095279	4.523286	6.09E-06	0.001411
Syt4	708.00613	-0.398028966	0.090641	-4.39128	1.13E-05	0.001958
Igtp	75.630598	0.806028185	0.186909	4.312405	1.61E-05	0.0021
Lgals3	1571.7909	0.552598677	0.12891	4.286714	1.81E-05	0.0021
Slc1a3	623.82314	-0.34041449	0.084233	-4.04134	5.31E-05	0.00521
Psmb8	196.57432	0.467983747	0.116619	4.012944	6.00E-05	0.00521
Ppp3r1	1656.5709	0.373640257	0.094373	3.959178	7.52E-05	0.005808
Serpina3n	1584.9554	-0.523047912	0.133548	-3.91656	8.98E-05	0.006243
Gnai1	706.75943	0.309410528	0.082344	3.757537	0.000172	0.010842
Gpr17	177.67872	-0.650235425	0.174262	-3.73137	0.00019	0.01103
Camk2g	618.96543	0.263545394	0.073687	3.576539	0.000348	0.018614
Ubqln1	803.86275	0.297993556	0.083851	3.553846	0.00038	0.018846
Hsd11b1	94.296318	0.507436922	0.143532	3.535346	0.000407	0.018869
Zfp365	374.74538	-0.4185622	0.130053	-3.2184	0.001289	0.051003
Trim2	609.99306	0.246205065	0.076601	3.214137	0.001308	0.051003
Gal3st1	202.27893	-0.634266118	0.198456	-3.196	0.001393	0.051003
H2-D1	583.68596	2.861827293	0.89549	3.195823	0.001394	0.051003
Plxnb3	146.94443	-0.591567604	0.187968	-3.14718	0.001649	0.054153
Atp1b1	2316.3187	-0.346394462	0.110419	-3.1371	0.001706	0.054153
Nefh	1370.7743	0.41403938	0.132086	3.134623	0.001721	0.054153
Ndufb5	661.00682	-0.35116781	0.112457	-3.12268	0.001792	0.054153
Cst3	16821.859	-0.264117921	0.086128	-3.06657	0.002165	0.060939
Olig2	67.286866	-0.833191719	0.272027	-3.0629	0.002192	0.060939
Ube3c	202.7953	-0.358628082	0.118112	-3.03634	0.002395	0.061271
Bhlhe41	456.78383	0.33878164	0.111846	3.029002	0.002454	0.061271
Ppp3cb	780.47166	0.245569469	0.081285	3.021092	0.002519	0.061271
Ndufa10	870.64724	0.209454518	0.069435	3.016558	0.002557	0.061271
Ube2d2a/l	569.02284	0.245863434	0.08267	2.974019	0.002939	0.068093
Mbp	10571.58	-0.395191879	0.133487	-2.96053	0.003071	0.068852
Atp6v1g2	380.07496	0.384558716	0.132057	2.912065	0.00359	0.077981
Stat1	245.34428	0.539446626	0.187101	2.883178	0.003937	0.082912
Ass1	203.44339	0.315929111	0.112264	2.814164	0.00489	0.099966
Ccl12	274.93028	0.334641934	0.120378	2.779922	0.005437	0.107967
Cd47	404.24568	0.275841478	0.100907	2.733622	0.006264	0.115905
Lpl	292.45375	0.613527874	0.224655	2.730978	0.006315	0.115905
Eid1	1016.362	0.171127387	0.062782	2.72572	0.006416	0.115905
Gng3	2011.2784	-0.276301708	0.101536	-2.72123	0.006504	0.115905
Tlr3	135.60776	-0.411231252	0.153351	-2.68163	0.007326	0.125251
Pias1	115.37041	-0.513002172	0.191505	-2.67879	0.007389	0.125251

Phyh	701.60741	0.224079372	0.08484	2.641209	0.008261	0.131516
Sparc	5736.9544	-0.308205387	0.116724	-2.64045	0.00828	0.131516
Acat2	237.80055	-0.259986178	0.098534	-2.63855	0.008326	0.131516
Ptgs2	10.821021	0.948223383	0.361378	2.623907	0.008693	NA
Syngr1	465.93995	-0.654449785	0.250533	-2.61223	0.008995	0.13893
Myrf	454.35386	-0.49035988	0.18836	-2.60332	0.009233	0.139494
Pdha1	738.92141	-0.300288314	0.115734	-2.59465	0.009469	0.140015
C1ra	47.39995	0.624244319	0.241838	2.581251	0.009844	0.140473
Cd74	520.77913	-0.748754015	0.290308	-2.57917	0.009904	0.140473
Jak1	480.13826	0.26342136	0.10274	2.563963	0.010348	0.143843
Klrd1	10.626503	0.948550259	0.372753	2.544713	0.010937	NA
Ncam1	1343.983	-0.382744187	0.151207	-2.53126	0.011365	0.154879
Klk6	6114.7618	-0.355451211	0.141141	-2.51841	0.011789	0.157085
Gbp2	83.527058	0.607795981	0.243419	2.496915	0.012528	0.157085
Sptan1	1061.6324	-0.431641037	0.173055	-2.49425	0.012622	0.157085
Iigp1	106.21205	1.135402854	0.455977	2.490042	0.012773	0.157085
Uchl1	450.66442	-0.505606978	0.203248	-2.48764	0.01286	0.157085
Tardbp	1462.6528	-0.181159821	0.072843	-2.48698	0.012883	0.157085
Aqp4	3482.9904	-0.247507562	0.100478	-2.46331	0.013766	0.164956
Nlrp1b	14.078748	-0.811418734	0.332368	-2.44132	0.014634	NA
Emp1	225.69789	-0.37434424	0.154412	-2.42432	0.015337	0.180664
Lsr	55.31964	0.503315761	0.209823	2.398762	0.016451	0.190553
Tap1	84.564769	0.512654266	0.21428	2.392445	0.016737	0.190687
Ccl6	213.54621	-0.329234701	0.138969	-2.36913	0.01783	0.199869
Irf8	80.793228	0.391043197	0.167107	2.340081	0.01928	0.210726
Gng2	517.72144	-0.378134063	0.161758	-2.33766	0.019405	0.210726
Itgax	137.61235	-0.485925202	0.209017	-2.32481	0.020082	0.214726
Itm2a	449.64604	0.300949066	0.130203	2.311383	0.020812	0.215846
Etv5	104.58979	0.323630893	0.140303	2.306662	0.021074	0.215846
Ppt1	948.88974	-0.279113858	0.121046	-2.30585	0.021119	0.215846
Ifit3	810.86692	0.799398516	0.35035	2.281716	0.022506	0.222001
Wdr35	166.25269	-0.29887702	0.131047	-2.28068	0.022567	0.222001
Clta	1863.2727	0.260818612	0.114455	2.278795	0.022679	0.222001
Kpna1	417.77049	-0.197184522	0.086845	-2.27053	0.023176	0.222411
Ptpn11	802.06758	0.305890991	0.135125	2.263769	0.023588	0.222411
Mal2	191.4395	-0.347837049	0.153756	-2.26226	0.023681	0.222411
Basp1	311.01778	0.216442609	0.095957	2.255632	0.024094	0.223268
Itgam	387.2596	-0.330842707	0.148205	-2.23233	0.025593	0.23109
Scp2	313.97869	-0.207232729	0.092839	-2.23218	0.025603	0.23109
Ptprz1	992.66044	-0.226116132	0.101799	-2.22121	0.026337	0.234669
Fam104a	179.00521	0.253566894	0.115021	2.204524	0.027488	0.240615
P2ry12	240.9802	-0.439382521	0.199785	-2.19928	0.027858	0.240615
Adam22	293.11604	-0.36461587	0.165985	-2.19669	0.028043	0.240615
Adcy1	160.7167	-0.32113326	0.147521	-2.17687	0.029491	0.24995
Skp1a	1720.3548	-0.195505423	0.090991	-2.14863	0.031664	0.261731
Itga7	52.495117	-0.492450647	0.229255	-2.14805	0.03171	0.261731
Mmut	427.32813	-0.363780953	0.170242	-2.13684	0.032611	0.261731

Selenos	147.3617	-0.224061952	0.104913	-2.1357	0.032704	0.261731
Acadsb	432.78647	-0.171818915	0.080478	-2.13497	0.032763	0.261731
F11r	190.46121	0.232570766	0.109191	2.129942	0.033176	0.262018
Kcnd2	78.789168	-0.393263901	0.185078	-2.12486	0.033599	0.262372
Cybb	279.48584	0.473326531	0.224192	2.11126	0.03475	0.266757
Bhlhe40	603.15625	0.26593308	0.126083	2.109193	0.034928	0.266757
Arntl	33.011424	0.400152107	0.191432	2.090306	0.03659	0.276416
Cx3cl1	108.37886	-0.322493467	0.156483	-2.06089	0.039313	0.290688
Galc	238.56595	-0.206162922	0.100037	-2.06086	0.039316	0.290688
Sh2d1a	5.7109429	1.018641099	0.49504	2.057695	0.039619	NA
Icam2	28.491535	0.492053364	0.240607	2.045048	0.04085	0.296031
Tnfrsf21	380.68661	-0.359203858	0.175681	-2.04464	0.040891	0.296031
Csf3r	111.63977	-0.320309064	0.157211	-2.03744	0.041605	0.296798
Nf1	221.50544	0.189908986	0.093321	2.035002	0.041851	0.296798
Fbln5	55.03523	-0.409430733	0.20245	-2.02238	0.043137	0.301927
Ube2n	1223.8695	0.223811526	0.110829	2.019429	0.043443	0.301927
Mertk	135.35243	-0.361011063	0.179653	-2.00949	0.044485	0.306109
Gad1	305.27413	-0.281984027	0.14178	-1.98888	0.046714	0.318299
Klrk1	5.6173365	0.911927986	0.458609	1.988466	0.04676	NA
Tspo	144.22557	0.281604655	0.142982	1.969514	0.048894	0.329916
Ip6k2	238.97648	-0.170793289	0.087538	-1.95108	0.051048	0.335584
Stab1	34.873178	0.489657313	0.2513	1.948499	0.051355	0.335584
Cltc	3503.3031	0.098696865	0.05071	1.946318	0.051617	0.335584
Clec7a	798.18188	0.374335638	0.192444	1.945162	0.051756	0.335584
Mtor	136.31499	-0.437007838	0.22504	-1.94191	0.052148	0.335584
Slc2a5	30.8667	0.432183543	0.223385	1.934705	0.053026	0.335668
Adcy5	208.46942	-0.256151654	0.132454	-1.93389	0.053127	0.335668
Fcrlb	8.9917274	0.731102565	0.378411	1.932032	0.053356	NA
Tfec	3.3334692	1.104798526	0.575055	1.921204	0.054706	NA
Stambpl1	135.80632	0.218185399	0.114271	1.909374	0.056214	0.350411
Kitl	306.30482	0.345020369	0.180885	1.907398	0.056469	0.350411
Mx1	17.092153	0.653812862	0.346324	1.887865	0.059044	0.363147
Gdnf	12.050057	0.593451528	0.317346	1.870046	0.061477	NA
Akt3	599.93662	0.294720389	0.157748	1.868305	0.06172	0.371504
Gjb1	441.64292	-0.350719505	0.187776	-1.86776	0.061796	0.371504
Igf1	61.765318	0.347301706	0.186153	1.865674	0.062087	0.371504
Fos	80.92334	-0.482032099	0.25899	-1.8612	0.062716	0.371504
Htr5a	19.171054	-0.46408079	0.24981	-1.85774	0.063206	0.371504
Fcgr1	253.54297	0.224988699	0.121449	1.852541	0.063948	0.371504
Olfml3	210.58738	0.527998352	0.285224	1.851173	0.064145	0.371504
Ncf1	150.33961	-0.324795979	0.176306	-1.84223	0.065441	0.375883
Adora2b	93.46206	-0.273809889	0.149115	-1.83624	0.066323	0.377821
Pik3cb	308.66911	0.139281628	0.076035	1.831815	0.066979	0.378458
Gprasp1	1012.6333	-0.177804253	0.097543	-1.82284	0.068328	0.382969
Chil1	358.53248	-0.251036719	0.138429	-1.81347	0.06976	0.387866
Chchd3	237.9922	0.29038794	0.161481	1.798278	0.072133	0.395316
Hmox1	217.6212	-0.213143872	0.11857	-1.79762	0.072238	0.395316

Erlec1	309.08565	-0.203312509	0.113407	-1.79278	0.073009	0.396415
Ret	119.20689	-0.248228371	0.139145	-1.78395	0.074432	0.39891
Gabra5	159.68841	0.284847998	0.159774	1.782816	0.074616	0.39891
Fbxw11	825.87879	0.175733796	0.09897	1.775634	0.075793	0.401399
Atp6v1b2	1561.6958	0.143987493	0.081213	1.772951	0.076237	0.401399
Spi1	155.69356	0.303951848	0.171965	1.767523	0.077141	0.403104
Gria1	238.15101	0.187814298	0.106632	1.761337	0.078181	0.405493
Camk2a	187.69915	-0.413220167	0.235158	-1.7572	0.078884	0.405598
Dnm3	965.06592	-0.191661742	0.109411	-1.75176	0.079816	0.405598
P2rx7	144.78139	0.189617658	0.108293	1.750962	0.079952	0.405598
Reep5	734.97757	-0.162109219	0.092988	-1.74333	0.081276	0.406632
Becn1	248.83702	-0.214164502	0.122868	-1.74304	0.081326	0.406632
Gfap	22049.388	-0.180222144	0.1039	-1.73457	0.082817	0.409599
Prkacb	172.04373	0.15892729	0.091707	1.732984	0.083098	0.409599
Zpr1	129.51459	-0.259195478	0.150258	-1.72501	0.084526	0.410434
Aars	297.46177	-0.173684558	0.100703	-1.72472	0.084577	0.410434
Ctsf	466.85062	-0.164375188	0.095447	-1.72216	0.08504	0.410434
Pmp22	598.38559	-0.221079433	0.129008	-1.71369	0.086586	0.414522
Slc38a1	746.34506	-0.214996552	0.125655	-1.71101	0.087079	0.414522
Rfx4	108.84778	-0.432110146	0.253374	-1.70542	0.088116	0.416602
Jak2	215.75661	-0.158718959	0.093443	-1.69856	0.089403	0.41983
Tcl1	8.8523714	0.675430019	0.399104	1.692364	0.090577	NA
Stmn1	1230.059	0.144623964	0.085531	1.690903	0.090855	0.423788
Dmd	158.26018	-0.371967449	0.220922	-1.68371	0.092239	0.425154
Syt13	64.512802	-0.477531687	0.283735	-1.68302	0.092372	0.425154
Ifnar1	271.71485	-0.202107061	0.120819	-1.67281	0.094365	0.429938
Erbb3	57.942357	-0.362471687	0.217122	-1.66944	0.095031	0.429938
Ppm1a	1018.9574	0.182742197	0.109541	1.668246	0.095267	0.429938
Dnm1	1390.6011	0.22327637	0.134966	1.65431	0.098064	0.439708
Nrxn1	354.84728	-0.17317563	0.105042	-1.64863	0.099224	0.442055
Hdc	16.963328	-0.426318927	0.26103	-1.63322	0.102423	0.44671
Olfr110	34.289962	-0.471484672	0.288782	-1.63267	0.102539	0.44671
Lpar6	82.233997	-0.256336306	0.157027	-1.63244	0.102588	0.44671
Khsrp	145.26126	-0.359046372	0.220106	-1.63124	0.10284	0.44671
Sec61a2	126.63314	-0.204284899	0.125483	-1.62799	0.103526	0.446899
Cd3d	6.0230777	0.86809331	0.535375	1.621467	0.104917	NA
Prkab2	60.336281	-0.378387972	0.233962	-1.61731	0.105812	0.453946
Gria2	222.60342	-0.246602281	0.153016	-1.61161	0.107046	0.455186
Grm1	79.197653	-0.240562064	0.149423	-1.60994	0.107411	0.455186
Crot	296.52563	-0.179197137	0.112219	-1.59685	0.1103	0.464598
Ina	766.23897	0.211992775	0.133136	1.592297	0.111318	0.46606
Slc17a7	10.444248	-0.750539849	0.472429	-1.58868	0.112132	NA
Trem2	666.33627	-0.240749026	0.151583	-1.58823	0.112235	0.467084
Eno2	768.32064	-0.279975263	0.177119	-1.58072	0.113942	0.471367
Cntf	334.37102	-0.267123602	0.169876	-1.57246	0.115843	0.474428
Cldn5	194.11017	0.293541435	0.186782	1.571574	0.116049	0.474428
Fth1	128692.88	0.18815523	0.119947	1.568649	0.11673	0.474428

Ifit1	129.39144	0.712194217	0.456467	1.560232	0.118705	0.476441
Flt1	79.377064	-0.437098508	0.280394	-1.55887	0.119027	0.476441
Atp6v1g1	383.31382	-0.143764209	0.092287	-1.5578	0.119282	0.476441
Atp9a	632.96663	-0.162221368	0.104353	-1.55455	0.120054	0.476552
Eif2b5	226.81059	0.182730149	0.117744	1.551923	0.120681	0.476552
Opcml	178.72277	0.235176125	0.152662	1.540503	0.123438	0.482215
Sugt1	677.99359	0.140723373	0.091365	1.540237	0.123503	0.482215
Hsd17b4	351.48855	-0.153854514	0.100978	-1.52364	0.127599	0.493717
Dlat	477.53543	0.131237727	0.086196	1.522559	0.127869	0.493717
Elmo1	340.24975	-0.196200785	0.129427	-1.51591	0.129541	0.495753
Igfbp5	238.83764	-0.60529287	0.400097	-1.51286	0.130314	0.495753
Ninj2	23.737128	0.391709542	0.259436	1.50985	0.131082	0.495753
Prkcd	226.71602	-0.254890782	0.168899	-1.50913	0.131266	0.495753
Acly	583.5735	0.138977465	0.092258	1.506406	0.131963	0.495753
C3	1498.7634	0.184503373	0.122913	1.501085	0.133334	0.498209
Slc8a1	612.80008	0.159964199	0.107536	1.487544	0.136871	0.505962
P2ry13	85.472892	-0.2244	0.151154	-1.48458	0.137655	0.505962
Il21r	25.69821	0.309405481	0.208705	1.482504	0.138206	0.505962
Hk1	817.27226	-0.115100829	0.077893	-1.47768	0.139494	0.505962
Islr2	8.7901542	0.548376398	0.372538	1.472	0.141021	NA
Sh3gl2	889.78371	0.11918041	0.080982	1.471699	0.141102	0.505962
Lamp1	2868.4823	-0.11604135	0.07887	-1.47131	0.141208	0.505962
Tbk1	125.47592	-0.167932413	0.114264	-1.46969	0.141646	0.505962
Atp2a2	1962.5764	0.105883646	0.072099	1.468591	0.141944	0.505962
Abcc3	27.89612	-0.306935579	0.209009	-1.46853	0.14196	0.505962
Hspa1a/b	31.875257	-0.633486759	0.435467	-1.45473	0.145744	0.512976
Gls	658.79433	-0.114040305	0.078452	-1.45363	0.14605	0.512976
Ccl3	291.82845	-0.182084757	0.125371	-1.45237	0.1464	0.512976
Msr1	63.479521	-0.470152597	0.324101	-1.45064	0.146881	0.512976
Nrcam	808.21759	-0.171822731	0.118663	-1.44799	0.147619	0.512976
Kcnab1	341.85207	0.143690461	0.099587	1.442864	0.149059	0.515403
Ptx3	89.912418	0.320406271	0.223525	1.433427	0.151736	0.519311
Thy1	1395.9536	0.130881889	0.091512	1.430223	0.152653	0.519311
B2m	8611.0494	0.199210993	0.139806	1.424914	0.154182	0.519311
Ywhah	2156.2754	0.138574065	0.09749	1.421413	0.155197	0.519311
Amigo1	213.09208	-0.176533174	0.124375	-1.41937	0.155792	0.519311
Fig4	174.17032	-0.156413237	0.110226	-1.41902	0.155894	0.519311
Gabrb3	366.40436	0.122650613	0.086556	1.417006	0.156481	0.519311
Kras	433.9137	0.213911409	0.15106	1.41607	0.156755	0.519311
Dnajc11	121.35632	-0.226739251	0.160211	-1.41526	0.156993	0.519311
Slc12a5	400.51012	-0.303718229	0.21503	-1.41245	0.157818	0.519311
Chrnb2	40.912861	-0.261469977	0.185381	-1.41044	0.158408	0.519311
Dld	963.74242	0.134107663	0.095374	1.406127	0.159686	0.521042
Chrm3	23.995339	-0.470808729	0.336827	-1.39778	0.16218	0.526707
Csnk2a1	821.62415	0.164162068	0.117707	1.394661	0.163118	0.527288
P2ry1	120.58565	0.168473099	0.12122	1.389814	0.164585	0.529277
Psmd14	876.16178	0.163591456	0.118219	1.383796	0.166421	0.529277

Scoc	1662.8108	0.142867866	0.103323	1.382736	0.166746	0.529277
Olig1	136.85573	-0.298269962	0.215727	-1.38263	0.166779	0.529277
Rlim	517.64835	-0.166935913	0.121056	-1.379	0.167895	0.529831
Mapk9	553.3045	0.170666869	0.124547	1.370303	0.170592	0.529831
Pak1	1239.878	-0.116864666	0.085285	-1.37029	0.170597	0.529831
Bdnf	49.804448	0.231033117	0.168633	1.370032	0.170677	0.529831
Amph	387.12264	0.154558631	0.113034	1.367366	0.171511	0.529831
Adora1	79.94243	-0.219829714	0.16091	-1.36617	0.171886	0.529831
Rasgrp1	39.853995	-0.342863887	0.25165	-1.36246	0.173053	0.529831
Cacnb2	81.299309	0.192552402	0.141487	1.360923	0.173538	0.529831
Mag	1077.4779	-0.129545792	0.095251	-1.36005	0.173815	0.529831
Mapk13	8.0136185	-0.536475885	0.394765	-1.35898	0.174154	NA
Lta4h	216.29231	0.116002207	0.085799	1.352019	0.176369	0.534963
Sqstm1	1593.4872	0.131693456	0.097556	1.349931	0.177038	0.534963
Cd244a	28.821191	0.358394183	0.268503	1.334784	0.181947	0.547416
Rapgef2	91.192181	-0.198405621	0.149333	-1.32862	0.183975	0.548888
Csnk2a2	237.03865	0.112884261	0.08524	1.324311	0.1854	0.548888
Atp8a2	258.61364	0.130204296	0.098511	1.321725	0.18626	0.548888
Nkg7	9.5843273	0.46710709	0.353881	1.319955	0.18685	NA
Agmo	181.88822	-0.158633516	0.12024	-1.31931	0.187065	0.548888
Bcas1	1897.043	0.193742189	0.147035	1.317665	0.187616	0.548888
Pecam1	77.541243	0.268312979	0.20375	1.316871	0.187882	0.548888
Ltc4s	10.746517	-0.460400978	0.350357	-1.31409	0.188815	NA
Dnajb9	446.6126	0.156678972	0.119288	1.313451	0.189031	0.548888
Ppp2r2b	228.11531	0.182856446	0.139294	1.312736	0.189272	0.548888
Tyrobp	4269.6744	-0.184709484	0.140792	-1.31193	0.189544	0.548888
Ncam2	517.10656	-0.127695036	0.097641	-1.3078	0.190941	0.55064
Cnot10	170.80909	-0.203937066	0.156909	-1.29972	0.193698	0.55271
Cntnap2	113.96544	-0.162199339	0.124948	-1.29813	0.194242	0.55271
Olfm3	141.72095	0.234260952	0.18065	1.296764	0.194712	0.55271
Ccnb1	47.735866	0.282305152	0.217993	1.295018	0.195314	0.55271
Gabrd	15.174883	-0.399213329	0.308408	-1.29443	0.195516	NA
Por	194.21341	0.136832369	0.105737	1.294086	0.195636	0.55271
Mef2a	325.69066	-0.11430013	0.088756	-1.2878	0.197816	0.556607
Pccb	261.32854	0.138154369	0.107495	1.285213	0.198718	0.556891
Tmem88b	14.288884	0.379788241	0.295628	1.284684	0.198903	NA
Hectd1	425.40137	-0.153610847	0.120887	-1.2707	0.203837	0.568942
Cdk5r1	62.584806	0.219864932	0.174345	1.261091	0.207276	0.576227
Cspg4	100.40111	-0.26940865	0.214257	-1.25741	0.208606	0.577614
Tspan7	1274.2608	-0.204525036	0.16316	-1.25352	0.210016	0.579212
Eloc	1505.4724	0.154113205	0.123503	1.247852	0.212085	0.582606
Xk	54.318142	0.203669628	0.163743	1.243838	0.213559	0.584034
Myorg	62.072712	-0.276864003	0.223557	-1.23845	0.215549	0.584034
Gusb	318.29154	-0.194654859	0.157257	-1.23782	0.215784	0.584034
Slc6a1	720.38009	-0.159091844	0.128577	-1.23733	0.215966	0.584034
Tmem100	26.40596	0.269404504	0.220099	1.224012	0.220948	0.594824
Dnaja2	366.75633	0.130491912	0.106776	1.222104	0.221668	0.594824

Kir3dl1/2	3.7545661	0.664465819	0.544626	1.220041	0.222449	NA
C1qa	5241.7278	-0.189567018	0.155685	-1.21763	0.223363	0.597068
Ctnnb1	2025.9804	-0.132987785	0.11027	-1.20602	0.227809	0.606468
Plekhb1	8409.8753	-0.109667853	0.091161	-1.20302	0.22897	0.606468
Tgfb1	212.73631	0.177369225	0.147695	1.200916	0.229784	0.606468
Dnm1l	604.44791	-0.097249496	0.081087	-1.19932	0.230405	0.606468
Xcl1	3.3868262	0.676217818	0.565679	1.195409	0.231927	NA
Unc79	449.74714	0.171852723	0.144433	1.189844	0.234108	0.606468
Egfr	51.742478	-0.254190867	0.214112	-1.18719	0.235153	0.606468
Acsl4	275.80934	0.104055845	0.087661	1.187021	0.235219	0.606468
Psen1	267.72087	-0.107193096	0.090323	-1.18677	0.235318	0.606468
Ywhae	5258.8499	0.187025919	0.157644	1.186384	0.235471	0.606468
Panx1	91.607231	-0.198852761	0.168154	-1.18257	0.236982	0.606468
Psmc6	1246.2019	0.181994035	0.154201	1.180241	0.237904	0.606468
Trac	35.053927	0.340416975	0.288551	1.179748	0.2381	0.606468
Atg12	332.27095	-0.104554254	0.088648	-1.17944	0.238224	0.606468
Tlr2	128.98382	0.182651081	0.155602	1.173838	0.24046	0.607476
Hmgb1	1304.9569	0.088473867	0.075408	1.173274	0.240686	0.607476
Entpd1	117.48408	0.158248363	0.135037	1.171888	0.241242	0.607476
Sox2	354.31756	-0.148687752	0.127365	-1.16742	0.243041	0.609797
Tgm1	103.08585	0.255027971	0.219419	1.162285	0.24512	0.612799
Arpc5l	511.38571	0.126936739	0.109577	1.15843	0.246689	0.614511
Pde1a	165.59418	0.177751196	0.154342	1.151673	0.249455	0.61713
Adgra2	48.676006	-0.290757878	0.252498	-1.15153	0.249516	0.61713
Tanc2	258.94131	-0.154490731	0.134743	-1.14656	0.251562	0.618502
Atp6v1d	912.5628	0.108648167	0.094818	1.145866	0.251851	0.618502
Vegfa	262.79514	-0.292896126	0.256616	-1.14138	0.253712	0.620879
Mto1	133.67173	-0.143679629	0.12625	-1.13806	0.255097	0.622079
Kcnq3	157.45338	-0.171976764	0.151852	-1.13253	0.257412	0.623862
Pnoc	16.855822	-0.301946521	0.266731	-1.13203	0.257624	0.623862
Fgf2	65.803218	-0.284180327	0.252022	-1.1276	0.259488	0.626195
Psma5	723.01154	-0.135796654	0.12098	-1.12248	0.26166	0.628913
Acat1	686.05783	-0.104274474	0.093046	-1.12068	0.262424	0.628913
Akt1	643.19784	-0.189277496	0.169546	-1.11638	0.264261	0.631138
Ptger4	20.772915	0.268841525	0.24147	1.113353	0.265557	0.631542
Egf	10.653555	-0.388404483	0.349937	-1.10993	0.26703	NA
Tln2	203.49956	0.147273462	0.132779	1.109161	0.267361	0.631542
Grik2	161.95454	-0.140751291	0.127002	-1.10826	0.267751	0.631542
Gad2	410.39215	0.121564437	0.110192	1.103209	0.269936	0.631542
Tmem47	806.96831	0.07614732	0.069145	1.101263	0.270782	0.631542
Kctd12	27.125107	-0.335635608	0.305085	-1.10014	0.271272	0.631542
Spp1	12229.762	-0.274650062	0.24979	-1.09952	0.271539	0.631542
Gjd2	41.899896	0.183890265	0.167458	1.098129	0.272148	0.631542
Esam	77.87286	0.218867229	0.1995	1.097077	0.272608	0.631542
Tomm20	1074.8225	0.106218339	0.097546	1.088904	0.276196	0.637729
Parp2	70.320105	-0.172077114	0.159145	-1.08126	0.279582	0.638564
Nefl	853.36151	-0.201309552	0.186268	-1.08075	0.279807	0.638564

Gpnmb	2207.5584	0.445009966	0.411893	1.080402	0.279963	0.638564
Abcb1a	236.19172	0.162559475	0.151056	1.076152	0.281859	0.638564
Cacna1b	47.971948	-0.311379564	0.289682	-1.0749	0.282419	0.638564
Rnf10	544.76841	0.07783929	0.072462	1.074215	0.282726	0.638564
Ccdc127	389.85738	0.108844044	0.10138	1.073627	0.28299	0.638564
Cd3g	4.7548169	0.510446472	0.477912	1.068077	0.285486	NA
Gabarapl1	2550.2403	0.063002213	0.059109	1.065862	0.286486	0.641721
Atg2b	201.98611	-0.09835087	0.092819	-1.0596	0.289326	0.641721
Rock2	368.65227	-0.084868838	0.080236	-1.05774	0.290174	0.641721
Aldh1l1	514.53156	0.097993458	0.092759	1.056435	0.29077	0.641721
Grin2a	34.552683	-0.233492204	0.2213	-1.05509	0.291383	0.641721
Nostrin	28.484702	0.257888012	0.244587	1.054383	0.291707	0.641721
Dlx1	13.402489	0.38278727	0.363264	1.053744	0.292	NA
Supt7l	67.176016	-0.180727708	0.172115	-1.05004	0.293699	0.641721
Sumo1	722.70323	0.182592844	0.174339	1.047341	0.294942	0.641721
Nrg1	187.12004	-0.152632391	0.145739	-1.0473	0.294962	0.641721
Cep68	42.26412	-0.193819053	0.185375	-1.04555	0.295767	0.641721
Tbp	122.97185	-0.158184535	0.151626	-1.04325	0.29683	0.641721
Vim	5245.7072	-0.126862376	0.121611	-1.04318	0.296864	0.641721
Ranbp9	570.92804	-0.112544255	0.107947	-1.04259	0.29714	0.641721
Wasf1	290.12385	0.112453156	0.10817	1.039597	0.298527	0.641721
Ap3m2	224.07183	0.121444628	0.116941	1.038509	0.299033	0.641721
Cnp	6236.4109	-0.087103879	0.083896	-1.03823	0.299162	0.641721
Sec23a	507.92717	-0.100527039	0.097225	-1.03396	0.301153	0.642121
Ywhag	2885.6642	-0.281309541	0.272093	-1.03387	0.301197	0.642121
Ifngr2	230.68169	0.126896856	0.123693	1.025902	0.304938	0.648109
Orc3	374.26851	-0.093083102	0.090978	-1.02314	0.306243	0.648899
Scn1a	684.85212	0.133505296	0.130755	1.021033	0.307239	0.64903
Diaph1	203.19174	0.110736454	0.109143	1.014597	0.310298	0.653506
Lars	235.29772	-0.151298755	0.149544	-1.01173	0.311666	0.654404
Rab3a	3487.7493	0.113944319	0.113094	1.007521	0.313684	0.656659
Atp2b4	176.76711	-0.110160002	0.109596	-1.00515	0.314825	0.657068
Tpsab1	1.9261988	-0.799604679	0.795543	-1.00511	0.314846	NA
Sybu	100.29081	-0.182854776	0.183376	-0.99716	0.318687	0.661492
Gabra3	360.92919	-0.099282732	0.09979	-0.99492	0.319774	0.661492
Trim68	34.849754	-0.214336492	0.215442	-0.99487	0.319801	0.661492
Nos1ap	52.202605	0.158221744	0.15967	0.990928	0.321721	0.663489
Adora3	21.521207	0.24709913	0.252084	0.980226	0.326974	0.672329
Serping1	254.64378	-0.230904182	0.237155	-0.97364	0.330233	0.676938
Fcgr4	68.200533	0.162646769	0.167642	0.970203	0.331946	0.676938
Cpne2	184.12568	0.106373989	0.109685	0.969816	0.332138	0.676938
Tnfrsf17	4.9215535	-0.465944332	0.484031	-0.96263	0.335731	NA
Slc7a8	55.150222	0.170034827	0.176662	0.962489	0.335804	0.678367
Prkdc	121.56528	-0.16178179	0.168427	-0.96054	0.336782	0.678367
Tbr1	2.8464574	0.682673455	0.711029	0.96012	0.336995	NA
Acsl3	920.71579	-0.102476828	0.106882	-0.95878	0.337668	0.678367
Syt1	407.06009	-0.135277583	0.141158	-0.95834	0.337889	0.678367

Slc25a12	419.52887	0.093921865	0.098013	0.958263	0.33793	0.678367
Adh5	779.17266	-0.084661773	0.088489	-0.95675	0.338696	0.678367
S100b	4291.5359	-0.083513413	0.087607	-0.95327	0.340452	0.679446
Lilrb4a	392.24821	0.184554079	0.19406	0.951015	0.341597	0.679446
Kcnv1	14.306965	0.275494113	0.289859	0.950441	0.341888	NA
B4galt5	104.95474	0.117090353	0.123267	0.949892	0.342167	0.679446
Ppp2ca	2257.3667	0.153035721	0.163539	0.935777	0.349388	0.691809
Bub3	446.3065	-0.080018872	0.085835	-0.93225	0.35121	0.69344
Hexb	5667.5092	0.102804069	0.110818	0.927688	0.35357	0.696121
Gnas	2693.9491	-0.125399957	0.136277	-0.92019	0.357476	0.701824
Ptgis	52.51672	0.296674598	0.324228	0.915019	0.360181	0.705144
Il34	147.1898	0.161873024	0.177614	0.911375	0.362098	0.706904
Sphk1	13.300197	-0.256213109	0.283024	-0.90527	0.365323	NA
Sox9	225.40581	-0.147108973	0.163527	-0.8996	0.368332	0.715726
Ctss	6103.8089	0.149901307	0.16728	0.89611	0.370194	0.715726
Gdpd2	73.79019	-0.141957923	0.158721	-0.89438	0.371116	0.715726
Bax	158.12954	-0.086888801	0.097186	-0.89404	0.371299	0.715726
Ubqln2	519.52957	0.077259225	0.0865	0.893171	0.371765	0.715726
Nos2	5.8390106	0.373302679	0.419177	0.89056	0.373165	NA
Mapt	575.78452	-0.171044153	0.192659	-0.88781	0.374643	0.719273
Nap1l2	150.46006	0.129210915	0.146514	0.881904	0.377829	0.723391
Rab1a	1932.8128	0.058700438	0.066823	0.87844	0.379705	0.723407
Prkcz	182.02205	-0.105037742	0.119974	-0.87551	0.381299	0.723407
Cd200	293.4306	-0.083766822	0.095801	-0.87438	0.38191	0.723407
Dagla	68.430352	-0.217337865	0.249257	-0.87194	0.383239	0.723407
Slc9b2	31.311907	-0.200965687	0.230876	-0.87045	0.384054	0.723407
Itpr1	155.26355	0.104647398	0.120284	0.87	0.3843	0.723407
Dlgap1	62.323491	0.21571604	0.248379	0.868495	0.385123	0.723407
Ahcyl1	1341.5154	0.06753252	0.078703	0.858069	0.390854	0.731869
Grk3	124.45417	-0.126245913	0.147401	-0.85648	0.391734	0.731869
Rorb	160.33573	0.097226749	0.114256	0.850952	0.394796	0.735612
Stat3	621.42614	0.100477793	0.118561	0.84748	0.396728	0.735792
Npas2	55.413957	-0.133652265	0.158026	-0.84576	0.397687	0.735792
Hdac2	429.48245	0.143478478	0.169782	0.845076	0.398069	0.735792
Nkiras1	397.21648	0.134449408	0.160704	0.836629	0.402801	0.739919
Snap25	3862.7104	0.08879827	0.10633	0.835118	0.403651	0.739919
Plp1	31388.409	-0.071240822	0.085419	-0.83401	0.404273	0.739919
Tie1	37.865975	0.169722113	0.203964	0.832119	0.405342	0.739919
Unc80	290.94121	0.168240231	0.202305	0.831618	0.405624	0.739919
Kcnq5	5.4234541	-0.563273312	0.680514	-0.82772	0.40783	NA
Cyp4x1	39.066986	0.150588842	0.182278	0.826149	0.40872	0.743613
Grin3a	232.49003	0.088972166	0.108237	0.822009	0.411072	0.745939
Il10	4.8292915	0.404073389	0.493542	0.818721	0.412945	NA
Aplp2	3003.7559	0.096015098	0.117595	0.816492	0.414219	0.749693
Glrb	1649.5558	0.08655022	0.106617	0.811784	0.416916	0.752614
Adgrg1	48.862233	-0.181692379	0.225144	-0.80701	0.419663	0.755611
Cacna2d3	69.541818	0.124042365	0.154164	0.804611	0.421044	0.756139

Th	10.995084	0.268544382	0.336539	0.797958	0.424895	NA
Map1lc3b	910.3592	-0.064250487	0.08056	-0.79754	0.425135	0.758741
Tada2b	104.88561	-0.137769167	0.172945	-0.79661	0.42568	0.758741
Ctsl	692.67526	-0.109247423	0.13738	-0.79522	0.426484	0.758741
Tenm4	85.462991	0.133733102	0.16836	0.794329	0.427004	0.758741
Atf2	227.55968	-0.137552511	0.173544	-0.79261	0.428006	0.758741
Ulk1	72.208847	-0.158127011	0.200154	-0.79003	0.429513	0.758741
Sirt5	72.602394	-0.111099214	0.140817	-0.78896	0.430135	0.758741
Cd200r1	18.73966	-0.201238324	0.25648	-0.78462	0.432678	0.759447
Chn1	609.86524	-0.079327007	0.101112	-0.78454	0.432721	0.759447
Atp2b3	99.086435	0.121717726	0.15664	0.777054	0.437127	0.765247
Apoe	15204.802	0.122184945	0.157995	0.773346	0.439318	0.76715
Sirpa	548.12334	0.106669023	0.138911	0.767895	0.44255	0.770857
P2ry6	33.283184	0.177519575	0.232042	0.765031	0.444253	0.77189
Ywhaq	3266.4069	-0.112765722	0.14906	-0.75651	0.449343	0.775798
Cd68	1217.6283	-0.117460834	0.155611	-0.75483	0.450348	0.775798
Atr	88.516236	-0.117453394	0.155865	-0.75356	0.451113	0.775798
Samm50	441.02722	-0.057820392	0.076769	-0.75317	0.451348	0.775798
Nptn	2093.0585	-0.088125324	0.117196	-0.75195	0.452084	0.775798
Gria3	574.52499	-0.097788928	0.130524	-0.7492	0.453735	0.776714
Gmpr2	133.0535	-0.094798158	0.127276	-0.74482	0.456378	0.779319
Ncr1	3.8504186	-0.414629584	0.557264	-0.74405	0.456849	NA
Slpi	9.5184486	-0.258408964	0.347719	-0.74315	0.457388	NA
Cd9	2679.7968	-0.165382948	0.225271	-0.73415	0.462856	0.784442
Egr1	141.63485	0.129803223	0.177091	0.732975	0.463574	0.784442
Atp5pb	1497.62	-0.093790967	0.128086	-0.73225	0.464017	0.784442
Rbx1	1170.4409	0.065806324	0.090197	0.729588	0.465642	0.784442
Psmd6	838.68343	-0.083975395	0.115154	-0.72925	0.465851	0.784442
Plcb4	309.34938	-0.127165362	0.174496	-0.72876	0.46615	0.784442
Cd19	5.9717202	-0.32313758	0.44342	-0.72874	0.466162	NA
Ddah1	651.801	-0.065021271	0.0897	-0.72487	0.46853	0.78534
Gabra1	254.65045	-0.09306721	0.128525	-0.72412	0.468993	0.78534
Fpr1	16.721101	0.213040463	0.295696	0.72047	0.471236	NA
Atp1a1	906.79417	-0.082814851	0.115094	-0.71954	0.471809	0.78534
Atp6v1a	1231.8436	-0.063965249	0.088912	-0.71942	0.471879	0.78534
Tomm70a	1138.0115	0.125759218	0.175122	0.718124	0.472681	0.78534
Rims1	146.4238	-0.148194468	0.206729	-0.71685	0.473464	0.78534
Adar	237.30685	-0.128228671	0.180791	-0.70926	0.478161	0.791243
Gpr84	19.189658	0.166054933	0.235789	0.704253	0.481275	0.794504
Serinc3	1483.948	-0.073145822	0.104257	-0.70159	0.482932	0.79535
Ccna1	4.2294614	0.353747628	0.507067	0.697635	0.485406	NA
Fcrls	861.25331	-0.112933485	0.1625	-0.69498	0.48707	0.800268
Cd209e	5.059266	0.347436836	0.502496	0.691422	0.4893	NA
Abca1	875.95671	-0.102156048	0.147961	-0.69042	0.489928	0.802506
Syn2	617.46133	-0.097389897	0.141476	-0.68839	0.49121	0.802506
Dusp3	236.97319	-0.084053964	0.12247	-0.68632	0.492511	0.802506
Hspa9	1525.7618	-0.041694208	0.060984	-0.6837	0.494167	0.802506

Dclk1	316.73745	-0.076267555	0.111615	-0.68331	0.49441	0.802506
Cd109	300.00941	-0.169179944	0.248134	-0.68181	0.49536	0.802506
Prf1	10.341763	0.22959862	0.33797	0.679345	0.496919	NA
Calm1	4176.7485	0.038778041	0.057349	0.676182	0.498925	0.803584
Aldh1a1	879.90142	0.059111142	0.087755	0.673596	0.500568	0.803584
Extl2	406.11619	0.069240209	0.103068	0.671792	0.501716	0.803584
Bin1	1082.0661	0.046576644	0.069727	0.667987	0.504142	0.803584
Abce1	360.36108	0.081307037	0.121786	0.667624	0.504374	0.803584
Camk2d	620.58239	-0.071385849	0.106974	-0.66732	0.504569	0.803584
Ank	955.45848	0.071594004	0.107612	0.6653	0.505859	0.803584
Slc24a3	92.140966	-0.099935329	0.150346	-0.6647	0.50624	0.803584
Rhob	203.86295	-0.092173007	0.139048	-0.66289	0.507403	0.803584
Fbxl3	490.43238	0.049851626	0.075236	0.662599	0.507587	0.803584
Tm4sf1	1153.2853	0.117488274	0.178728	0.657359	0.51095	0.805685
Eif2ak1	229.13866	0.077017007	0.118222	0.651463	0.514748	0.805685
H2-T23	173.98888	-0.101977261	0.156718	-0.65071	0.515237	0.805685
Ptprm	155.86026	0.07798036	0.120331	0.64805	0.516953	0.805685
Ckb	9582.6565	-0.056410168	0.087108	-0.64759	0.51725	0.805685
Opa1	328.39887	0.084226618	0.130162	0.647092	0.517573	0.805685
Tjp1	607.47533	-0.08379666	0.129805	-0.64556	0.518567	0.805685
Lyz2	2666.4165	0.22397552	0.347755	0.644061	0.519536	0.805685
Cttnbp2nl	66.840573	0.128400959	0.199975	0.642087	0.520817	0.805685
Alas1	174.61888	0.070188072	0.109448	0.641293	0.521332	0.805685
Prkag1	542.49514	0.091728288	0.143186	0.640621	0.521769	0.805685
Cd14	142.8712	-0.081149483	0.127148	-0.63823	0.523325	0.805685
Trim45	46.453852	-0.12599596	0.197748	-0.63715	0.524025	0.805685
Inpp5f	769.62006	-0.074053558	0.11654	-0.63544	0.525144	0.805685
Epb41l3	762.70484	-0.069974895	0.111563	-0.62722	0.530513	0.812128
Psmd10	160.71376	-0.085608734	0.137687	-0.62176	0.534099	0.813716
Abcg2	306.3544	0.095554246	0.153861	0.621042	0.534572	0.813716
Klhl12	144.54819	0.069585435	0.112181	0.620297	0.535062	0.813716
Gsn	1521.438	0.120685496	0.196121	0.615363	0.538315	0.816875
Nsf	2974.6542	0.05216507	0.085041	0.613413	0.539603	0.817046
Rad23b	559.75371	-0.049711735	0.081336	-0.61119	0.541073	0.81749
Pllp	260.25591	-0.072368968	0.118806	-0.60914	0.542434	0.817769
Psmc2	939.22305	-0.041278178	0.068297	-0.60439	0.545585	0.81849
Cd33	101.8155	0.071109803	0.117953	0.602863	0.5466	0.81849
Crkl	152.57032	-0.07682404	0.127697	-0.60161	0.547434	0.81849
Mbtps1	181.23043	0.072866567	0.122249	0.596051	0.551141	0.81849
Ptk2b	66.871906	-0.089373775	0.150324	-0.59454	0.552151	0.81849
C1s1	33.022355	0.133286759	0.224883	0.592693	0.553387	0.81849
Usp2	182.32667	0.093266656	0.157416	0.592484	0.553527	0.81849
Ticam1	43.182022	-0.112796083	0.190379	-0.59248	0.553528	0.81849
Tsc1	154.35259	-0.06191568	0.104766	-0.59099	0.554528	0.81849
Trim35	103.20212	0.091588908	0.155039	0.590748	0.554689	0.81849
Scn8a	194.97448	0.100529932	0.170964	0.588019	0.556519	0.819041
Atrn	466.81743	-0.064906645	0.110634	-0.58668	0.557419	0.819041

Tbx21	2.7507575	0.372799421	0.644741	0.578216	0.563119	NA
Pcca	228.72748	0.058195606	0.101251	0.574765	0.56545	0.827977
Pmepa1	84.964695	-0.08938097	0.155761	-0.57383	0.566081	0.827977
Grn	1142.1142	0.087918646	0.153606	0.572365	0.567075	0.827977
Vdac1	1539.1138	-0.040143285	0.070684	-0.56792	0.570087	0.829343
Ccl2	144.1617	0.111874822	0.197293	0.567049	0.570681	0.829343
Cd3e	3.9141075	0.312678813	0.551942	0.566506	0.57105	NA
Prkce	230.83143	0.056457557	0.100006	0.564542	0.572385	0.829343
Il18	220.83325	-0.061653863	0.109828	-0.56136	0.574549	0.829343
Casp3	119.06576	-0.06776099	0.121609	-0.5572	0.577389	0.829343
Cd40	10.181264	-0.187016123	0.335707	-0.55708	0.577471	NA
Reck	50.857858	0.103897946	0.187607	0.553806	0.579712	0.829343
Ube4b	201.25868	-0.050769756	0.092067	-0.55144	0.581329	0.829343
Trim3	331.86613	0.054751125	0.099485	0.550343	0.582084	0.829343
Gas6	705.33691	0.063648087	0.11577	0.549782	0.582469	0.829343
Tpsb2	4.3693305	-0.281084939	0.512709	-0.54824	0.58353	NA
Pdgfra	221.30277	0.05938663	0.108362	0.548037	0.583666	0.829343
Rxfp1	12.468182	0.171377889	0.313171	0.547234	0.584218	NA
Atp5b	2933.2339	0.038828458	0.071157	0.545677	0.585288	0.829343
Gsk3b	625.86464	-0.067635756	0.124242	-0.54439	0.586175	0.829343
Adrb1	28.613447	-0.169980099	0.312572	-0.54381	0.586572	0.829343
Grb2	378.79037	0.048415281	0.089072	0.543552	0.58675	0.829343
Atp6v0d1	1220.1936	0.069256346	0.127468	0.543325	0.586906	0.829343
Prkci	169.63879	0.062231479	0.114774	0.54221	0.587674	0.829343
Amigo2	48.795451	0.118979107	0.2198	0.541306	0.588297	0.829343
Drd1	15.392687	0.176734308	0.328896	0.537355	0.591022	NA
Tmsb4x	48491.285	-0.061847477	0.115374	-0.53606	0.591915	0.831331
Orc5	161.41908	-0.075567238	0.141336	-0.53467	0.592881	0.831331
Eomes	4.7064735	0.266374696	0.499676	0.533095	0.593968	NA
Tpi1	120.36155	-0.066766873	0.125249	-0.53307	0.593983	0.831331
Cst7	1203.2802	0.081786641	0.15429	0.530085	0.596053	0.831331
Smad3	60.344706	-0.089491677	0.169164	-0.52902	0.596789	0.831331
Siglech	165.67347	-0.084521287	0.159901	-0.52858	0.597094	0.831331
Pdpk1	224.03584	0.053182874	0.100885	0.527164	0.59808	0.831331
Trim46	118.59754	-0.095607719	0.181994	-0.52533	0.599351	0.831435
Orc4	352.0015	-0.042148596	0.081344	-0.51815	0.604351	0.835474
Arpc2	692.72332	0.106350984	0.205604	0.517262	0.604973	0.835474
Cdc23	200.80966	-0.052210138	0.101398	-0.5149	0.606621	0.835474
Mapk8	305.91524	-0.039652238	0.077106	-0.51426	0.607071	0.835474
Tnf	8.4518334	0.184678652	0.360876	0.511751	0.608825	NA
Thrb	71.646637	0.078166153	0.152901	0.51122	0.609197	0.836742
Myo5a	718.40705	-0.047192024	0.092729	-0.50892	0.610807	0.8373
Lrrc7	93.346381	-0.141267482	0.279626	-0.5052	0.613417	0.839222
Pgam1	1096.1537	-0.058933235	0.117307	-0.50238	0.615398	0.840278
Cdk7	292.87604	0.065620692	0.131998	0.497133	0.619095	0.843669
Slc46a1	32.752345	0.109533982	0.221494	0.494524	0.620936	0.844522
Nwd1	89.444648	-0.152723495	0.310301	-0.49218	0.622593	0.845122

Hpgds	159.69213	-0.087177946	0.178281	-0.48899	0.624847	0.845548
Cul2	340.57513	0.070645207	0.144677	0.488295	0.625341	0.845548
Atp6v1c1	1339.3508	0.029723076	0.061187	0.485777	0.627125	0.846315
Cd8a	5.7871623	-0.219506808	0.457116	-0.4802	0.631086	NA
B3gnt5	8.9275413	-0.168503295	0.353848	-0.4762	0.63393	NA
Gphn	267.02016	-0.046390519	0.098711	-0.46996	0.638382	0.859319
C3ar1	482.82574	-0.064890276	0.138427	-0.46877	0.639235	0.859319
Entpd2	72.882917	-0.077484653	0.167378	-0.46293	0.643413	0.863266
Hadh	284.07723	0.068430242	0.149835	0.456705	0.647883	0.867445
Pros1	472.79715	0.051993218	0.114241	0.455119	0.649023	0.867445
Hacd2	469.82125	0.05136745	0.115735	0.443839	0.657159	0.871967
Pdhb	1044.241	-0.0403819	0.090985	-0.44383	0.657165	0.871967
Atp5g3	1258.7812	0.033388624	0.075282	0.443511	0.657396	0.871967
Map2	1296.453	-0.055129267	0.124384	-0.44322	0.657608	0.871967
Abi2	308.20802	0.03404764	0.077238	0.440815	0.659347	0.871967
Nuak1	65.119644	-0.07435622	0.16899	-0.44	0.659935	0.871967
Lgmn	3410.7863	0.052774949	0.120746	0.437074	0.662058	0.873112
Stat2	112.72922	-0.07878683	0.185094	-0.42566	0.670357	0.882382
Fcgrt	201.42225	0.075120518	0.180352	0.416521	0.677029	0.888145
Ms4a4a	62.843537	0.092981441	0.22373	0.415596	0.677706	0.888145
Gabra4	57.534439	-0.065114172	0.157122	-0.41442	0.678569	0.888145
Tlr4	92.991465	0.089493872	0.219685	0.407373	0.683734	0.893224
Mapk1	967.38965	0.033931109	0.083697	0.405405	0.68518	0.893236
Nlrp3	19.871636	0.120213454	0.298802	0.402318	0.68745	0.893236
Nes	215.15667	0.049633102	0.12343	0.402116	0.687599	0.893236
Glra3	72.178764	-0.075199461	0.18836	-0.39923	0.689721	0.894322
Mapk14	298.11398	-0.037535996	0.095355	-0.39364	0.693843	0.897232
Slc17a6	240.95282	-0.042427515	0.108043	-0.39269	0.694548	0.897232
Ank2	1617.7403	0.049047407	0.126299	0.388343	0.697762	0.899041
Cdc7	29.860996	0.096292655	0.250404	0.38455	0.700571	0.899041
Epb41l2	652.44697	0.031680394	0.082395	0.384493	0.700613	0.899041
Timp2	3323.634	0.047522473	0.123819	0.383806	0.701122	0.899041
Ceacam3	4.3926079	-0.192754811	0.506865	-0.38029	0.703732	NA
Shc3	210.77443	-0.095028383	0.250929	-0.37871	0.704906	0.901376
Cysltr1	33.629599	0.082193596	0.217526	0.377857	0.705537	0.901376
Gabrg2	297.44308	-0.045474974	0.121247	-0.37506	0.707615	0.901844
Mafb	79.777651	0.065533918	0.176559	0.371174	0.710508	0.901844
Ywhaz	2106.7663	-0.031052823	0.083688	-0.37106	0.710596	0.901844
Il1b	11.38121	0.122126633	0.32923	0.370946	0.710678	NA
Ap2m1	1823.1087	0.027870024	0.075681	0.368258	0.712681	0.901844
Tnfrsf11a	63.571391	0.055506904	0.150759	0.368184	0.712736	0.901844
Lamp2	2354.202	0.064318353	0.175299	0.366906	0.713689	0.901844
Sgce	463.60375	0.075337317	0.208255	0.361754	0.717536	0.903291
Cd84	573.26415	-0.053232972	0.14892	-0.35746	0.720747	0.903291
Rasgrf1	203.80873	0.034138798	0.09551	0.357438	0.720764	0.903291
Dlx2	8.271244	0.139535742	0.39114	0.356741	0.721285	NA
Calm2	2043.0813	-0.031067588	0.087238	-0.35613	0.721746	0.903291

Xpnpep1	252.22238	0.032834939	0.092303	0.35573	0.722043	0.903291
Gzmb	5.6408471	0.15406034	0.434045	0.354941	0.722634	NA
Rasa1	141.23289	0.047659814	0.134578	0.354143	0.723231	0.903291
Cacna2d1	102.04318	0.03962564	0.112609	0.351886	0.724924	0.903291
C1qc	3517.3163	-0.059790512	0.170113	-0.35147	0.725232	0.903291
Lag3	156.18991	0.056054824	0.162356	0.345259	0.729899	0.907478
Klrb1	6.7145546	0.140667665	0.410893	0.342347	0.73209	NA
Mad2l1	23.059681	-0.089346559	0.262554	-0.3403	0.733632	0.909013
Nlk	79.582223	0.046014645	0.135281	0.340141	0.73375	0.909013
C9orf72	230.3797	-0.039521094	0.117029	-0.3377	0.735587	0.909667
Shank2	23.207145	-0.099464463	0.296243	-0.33575	0.737057	0.909866
Ube2g1	301.52413	0.032867011	0.098743	0.332853	0.739245	0.910949
Immt	648.25244	0.02453737	0.074579	0.329013	0.742146	0.912592
Dnajc5	929.71819	0.023446625	0.071803	0.32654	0.744016	0.912592
Cnr1	163.93961	-0.036776176	0.112853	-0.32588	0.744518	0.912592
Grm2	13.15262	0.097314874	0.302188	0.322035	0.747427	NA
Ptprc	133.91387	-0.042673707	0.132926	-0.32103	0.748184	0.913439
Slc10a6	7.6457008	-0.123203578	0.38495	-0.32005	0.74893	NA
Sall1	213.00812	-0.036261939	0.113377	-0.31983	0.749094	0.913439
Gnb1	1586.6882	0.021728347	0.067952	0.319758	0.749152	0.913439
Acot13	549.73831	0.026752307	0.084644	0.316058	0.751958	0.914931
Gla	180.52718	0.036352377	0.116046	0.313258	0.754085	0.914931
Vdac3	1646.8446	-0.032448509	0.103688	-0.31294	0.754324	0.914931
Il1a	24.357476	0.070974084	0.228948	0.31	0.756561	0.915141
Ermn	2362.067	0.03775851	0.122097	0.309251	0.757131	0.915141
Sox10	410.04751	-0.034705639	0.113267	-0.3064	0.759297	0.916165
Fa2h	228.96798	-0.045923692	0.151419	-0.30329	0.76167	0.917437
Cd4	7.3250888	-0.137434146	0.456666	-0.30095	0.763452	NA
Cux2	54.724877	0.055375628	0.185628	0.298315	0.765462	0.920409
Foxp3	3.964922	0.155968396	0.525104	0.297024	0.766449	NA
Tusc3	397.48476	-0.02681011	0.091238	-0.29385	0.768875	0.921461
Atg5	244.36213	0.035655614	0.121403	0.293697	0.768989	0.921461
C1qb	2970.1525	0.041976573	0.14637	0.286784	0.774278	0.926202
Aif1	762.25466	-0.039343975	0.140265	-0.2805	0.779096	0.929185
Nmnat2	464.22378	-0.060889025	0.217429	-0.28004	0.779446	0.929185
Cyfip2	939.84673	0.028597296	0.103726	0.275701	0.782777	0.93069
Cdc42	3958.9934	0.022922087	0.083381	0.274908	0.783387	0.93069
Cacna1e	64.764426	-0.064815342	0.241846	-0.268	0.788697	0.9354
Plaa	132.8834	-0.035282267	0.133478	-0.26433	0.791526	0.936624
Rnasel	62.542146	-0.045558279	0.173118	-0.26316	0.792424	0.936624
Gria4	438.53207	0.026284729	0.100945	0.260387	0.794566	0.93756
Arl6ip5	733.42367	0.028210448	0.109468	0.257705	0.796635	0.938409
Csf1	620.93519	0.028050349	0.113458	0.247231	0.804729	0.940245
Kcnd1	30.02245	-0.061366658	0.248531	-0.24692	0.804972	0.940245
Map3k4	124.74483	-0.029872167	0.121672	-0.24551	0.806059	0.940245
Ppm1l	143.37157	-0.027169221	0.110967	-0.24484	0.80658	0.940245
Hacd3	531.52776	0.02917677	0.120146	0.242845	0.808125	0.940245

Serpini1	449.37186	-0.032763674	0.137401	-0.23845	0.81153	0.940245
Trim17	26.280752	0.084271456	0.356832	0.236166	0.813304	0.940245
Ctsd	33373.344	-0.044892408	0.190837	-0.23524	0.814023	0.940245
Rtn4	2414.7838	-0.025579437	0.109272	-0.23409	0.814915	0.940245
Dock3	321.42426	0.038281496	0.164539	0.232659	0.816027	0.940245
Slc25a4	5283.5359	0.028938002	0.124464	0.232501	0.816149	0.940245
Ccr5	113.87532	0.036318506	0.156609	0.231905	0.816612	0.940245
Slco2b1	36.665288	-0.049205942	0.2134	-0.23058	0.81764	0.940245
Pik3r1	675.42084	0.017114811	0.074263	0.230461	0.817734	0.940245
Mapk10	1043.1395	0.017764409	0.077407	0.229492	0.818486	0.940245
Ggta1	105.87415	0.032737376	0.15228	0.214981	0.829782	0.947859
Cul1	753.55154	-0.018317197	0.085486	-0.21427	0.830335	0.947859
Mki67	50.103958	-0.037235487	0.173992	-0.21401	0.830542	0.947859
P2ry14	27.964678	-0.043797179	0.206703	-0.21188	0.832197	0.947859
Cd34	756.55184	-0.032949155	0.158383	-0.20803	0.835202	0.947859
Glul	7290.39	0.023782173	0.115116	0.206594	0.836327	0.947859
Il10ra	60.920564	0.038476618	0.186256	0.206579	0.836339	0.947859
Trat1	6.6182047	0.089217041	0.432438	0.206312	0.836547	NA
Anapc1	335.00706	0.018306316	0.088754	0.206258	0.836589	0.947859
Fgf13	268.12026	-0.033632324	0.163873	-0.20523	0.837389	0.947859
Atp2b2	754.04746	-0.042246464	0.20985	-0.20132	0.840451	0.949778
Pth2r	6.6278176	0.0850777	0.424267	0.200529	0.841067	NA
E2f3	96.94407	-0.031112485	0.156415	-0.19891	0.842333	0.950141
Trim37	859.48813	-0.018463924	0.09353	-0.19741	0.843506	0.950141
Ms4a2	6.6290822	-0.080907649	0.412994	-0.19591	0.844684	NA
Jun	359.90829	-0.024896129	0.127489	-0.19528	0.845173	0.950478
Map2k4	569.16055	0.019902722	0.103926	0.191508	0.848128	0.95226
Nckap1	1436.6721	-0.017544624	0.094552	-0.18556	0.852793	0.955954
Ugt8a	655.84655	-0.030673069	0.168438	-0.1821	0.855502	0.957446
Ngf	15.761134	0.054330737	0.301353	0.180289	0.856925	NA
Axl	795.22894	-0.023689502	0.135878	-0.17434	0.861595	0.960604
Tiam1	65.865102	-0.031075064	0.181466	-0.17124	0.864031	0.960604
Asb10	15.032871	-0.049509186	0.293781	-0.16852	0.866171	NA
Pgk1	4381.6827	0.013398108	0.079809	0.167877	0.86668	0.960604
Adgrl4	87.282994	0.02761	0.165436	0.166893	0.867455	0.960604
Acadm	338.87756	0.029285532	0.176809	0.165633	0.868446	0.960604
Anapc7	22.490853	0.037861166	0.229025	0.165314	0.868697	0.960604
Cd163	44.58807	0.050984806	0.308775	0.165119	0.86885	0.960604
Cables1	121.06994	0.019926502	0.121175	0.164444	0.869381	0.960604
Gas7	293.27242	0.016987826	0.10443	0.162671	0.870777	0.960619
Camk4	58.405443	-0.027466956	0.173249	-0.15854	0.874031	0.960757
Kcnk13	91.557913	-0.025362865	0.160778	-0.15775	0.874653	0.960757
Clcf1	23.812423	-0.035053491	0.223346	-0.15695	0.875286	0.960757
Atp6v1h	1772.192	0.019542605	0.127695	0.153041	0.878366	0.960757
Mtmr4	162.67824	0.017095241	0.111718	0.153022	0.878381	0.960757
Ptk2	207.94065	0.016841893	0.11081	0.151988	0.879196	0.960757
Cdc27	258.11083	0.015098588	0.102293	0.147601	0.882658	0.961941

Pkd2	167.6339	0.015866398	0.107856	0.147107	0.883048	0.961941
Sall3	65.765663	0.031921362	0.221621	0.144036	0.885472	0.963072
Ms4a1	5.8310065	0.063618227	0.446346	0.142531	0.886661	NA
Cd6	12.189256	0.045323481	0.318446	0.142327	0.886822	NA
Abcc8	20.759258	0.057793195	0.407436	0.141846	0.887202	0.963446
Fabp5	340.66636	-0.014138458	0.102737	-0.13762	0.890543	0.963945
Txn1	2127.2025	-0.025131078	0.188148	-0.13357	0.893742	0.963945
Grm7	191.88212	-0.027438787	0.205977	-0.13321	0.894025	0.963945
Prkar1a	2255.1982	-0.007966248	0.05995	-0.13288	0.894287	0.963945
Bace1	248.07341	0.012290338	0.092764	0.13249	0.894597	0.963945
Asns	253.82379	0.013567057	0.1056	0.128476	0.897772	0.965483
Ccnh	585.20175	0.008979254	0.070606	0.127174	0.898802	0.965483
Cfi	5.8174736	-0.054424287	0.429398	-0.12675	0.899142	NA
Il6	6.2536367	-0.056747036	0.449551	-0.12623	0.899549	NA
Sod1	2280.3411	0.01657747	0.132769	0.12486	0.900635	0.965959
Cxcl15	6.1734475	0.050915705	0.419354	0.121415	0.903363	NA
Rtn1	943.02622	0.014987749	0.127747	0.117324	0.906604	0.970862
Chrm2	109.54754	0.017702906	0.158212	0.111894	0.910908	0.9725
Atp6v1e1	1217.0567	-0.013573208	0.122735	-0.11059	0.911942	0.9725
Lamtor3	454.61765	0.013602606	0.123549	0.110099	0.912331	0.9725
Cyp2u1	76.197446	0.015313197	0.147995	0.103471	0.917589	0.974779
Tgfbr1	300.48997	0.011827327	0.114854	0.102977	0.917981	0.974779
App	6358.5237	0.009565614	0.093688	0.102101	0.918677	0.974779
Meaf6	213.82615	0.010004589	0.102449	0.097655	0.922207	0.976257
Prkcq	75.719874	0.014260228	0.149441	0.095424	0.923978	0.976257
Stxbp1	1054.4802	0.008786297	0.098165	0.089505	0.92868	0.976257
Slc25a17	232.05722	0.011816784	0.133376	0.088598	0.929402	0.976257
Gpr34	309.64986	-0.019533948	0.228441	-0.08551	0.931856	0.976257
Serpine2	2694.6591	0.011959587	0.140119	0.085353	0.931981	0.976257
Il4	7.8869441	0.031740013	0.378382	0.083883	0.933149	NA
Plxdc2	673.16485	-0.00975268	0.118055	-0.08261	0.934161	0.976257
Adcyap1r1	546.85604	0.009454444	0.114626	0.082481	0.934265	0.976257
Rab6b	1172.3062	0.007176695	0.087661	0.081869	0.934751	0.976257
Srpr	469.73623	-0.009395516	0.116334	-0.08076	0.93563	0.976257
Ptpn1	248.78155	-0.010431033	0.131317	-0.07943	0.936687	0.976257
Mkrn1	567.04727	0.006231898	0.078751	0.079134	0.936926	0.976257
B4galt6	392.56633	0.008115322	0.10669	0.076065	0.939368	0.976795
Gzma	7.9698804	0.030653432	0.415773	0.073726	0.941228	NA
Gpm6a	2720.1071	0.007503585	0.102242	0.073391	0.941495	0.976795
C4a/b	2281.6295	0.009641013	0.133028	0.072474	0.942225	0.976795
Prkab1	107.81487	-0.011420395	0.159907	-0.07142	0.943064	0.976795
Syn1	826.94174	0.010697971	0.15399	0.069472	0.944614	0.976945
Got1	1375.2083	-0.006129322	0.095607	-0.06411	0.948883	0.977242
Braf	210.05068	0.009239331	0.148316	0.062295	0.950328	0.977242
Cx3cr1	418.47557	-0.006407605	0.10575	-0.06059	0.951684	0.977242
Dync1li1	108.51668	0.007420185	0.12285	0.060401	0.951837	0.977242
Srgn	440.90363	0.007063444	0.117903	0.059909	0.952228	0.977242

Lair1	66.522335	0.011768688	0.201121	0.058516	0.953338	0.977242
S100a10	2109.7294	-0.007124754	0.125559	-0.05674	0.954749	0.977247
Spib	8.1523556	0.021319034	0.420593	0.050688	0.959574	NA
Tmem119	35.033241	-0.010705447	0.22882	-0.04679	0.962684	0.98392
Ctsw	8.2313086	-0.018320706	0.409173	-0.04478	0.964287	NA
Myct1	18.497495	0.012433505	0.277713	0.044771	0.96429	0.984114
Cpa3	6.744392	0.018895699	0.428936	0.044052	0.964863	NA
Cntnap1	214.21161	-0.006133918	0.144627	-0.04241	0.96617	0.984587
Map2k1	887.2141	-0.005389717	0.134063	-0.0402	0.967931	0.984938
Blk	6.1149754	-0.016804953	0.472958	-0.03553	0.971656	NA
Cd8b1	6.8690001	-0.013230193	0.416686	-0.03175	0.974671	NA
Adamts1	174.74189	-0.003136984	0.113083	-0.02774	0.977869	0.992975
Btrc	281.36148	0.004771599	0.178612	0.026715	0.978687	0.992975
Calm3	3056.3168	-0.003103315	0.134582	-0.02306	0.981603	0.994482
Hcn1	167.03455	-0.001849479	0.132252	-0.01398	0.988842	0.996775
Emcn	47.110918	0.002570422	0.185896	0.013827	0.988968	0.996775
Arpc1a	1076.75	0.001212544	0.100735	0.012037	0.990396	0.996775
Lrrc25	18.238868	0.003125998	0.261644	0.011948	0.990467	0.996775
Arg2	9.9303458	0.003607697	0.372376	0.009688	0.99227	NA
Slc12a2	858.3105	0.001561853	0.175358	0.008907	0.992894	0.996775
Csf1r	898.80693	-0.000825853	0.099572	-0.00829	0.993382	0.996775
Slc9a6	865.51832	-0.001478244	0.193575	-0.00764	0.993907	0.996775
Ctsb	6332.0072	0.001358847	0.234733	0.005789	0.995381	0.996815
Tlr7	59.414304	-0.000610468	0.208851	-0.00292	0.997668	0.997668

Supplementary Table S4. Differential gene expression list of prodromal stage α-Syn PFF seeded MhcII-/- vs MhcII+/+ mice. baseMean, mean transcript levels across all samples; log2FoldChange, log of fold change of RNA; lfcSE, standard error of log of fold change; stat, Wald statistics; padj, p value adjusted for multiple comparison.

	baseMean	log2FoldChange	lfcSE	stat	pvalue	padj
Trim26	135.2848	1.391395139	0.106641	13.04743	6.57E-39	4.67E-36
Mog	2847.1427	0.961780076	0.100196	9.598993	8.07E-22	2.87E-19
Mmut	466.05362	-0.550865917	0.087085	-6.32563	2.52E-10	5.97E-08
Ptpn11	848.14013	0.276545573	0.047774	5.788666	7.09E-09	1.26E-06
Hsp90ab1	3132.9353	0.503154043	0.105245	4.780784	1.75E-06	0.000248
Ywhaq	4763.8848	-0.343132831	0.073909	-4.64266	3.44E-06	0.000407
Cd47	548.01424	0.35066552	0.077495	4.525003	6.04E-06	0.000613
Acat2	430.60693	-0.327045236	0.076529	-4.27349	1.92E-05	0.001708
Gal3st1	220.52337	-0.574816242	0.13985	-4.11023	3.95E-05	0.002005
Gnai1	1114.7661	0.221804409	0.053937	4.112267	3.92E-05	0.002005
Ifit3	295.26495	0.639889391	0.15541	4.11742	3.83E-05	0.002005
Ndufb5	964.21463	-0.431465232	0.103437	-4.1713	3.03E-05	0.002005
Pdha1	895.02342	-0.279473576	0.067114	-4.16418	3.12E-05	0.002005
Tlr3	99.508047	-0.666945777	0.158929	-4.19651	2.71E-05	0.002005
Kpna1	545.93047	-0.249454831	0.061997	-4.02368	5.73E-05	0.002542
Rhob	210.94125	-0.359880252	0.089114	-4.03845	5.38E-05	0.002542
H2-D1	196.86521	3.194902692	0.798076	4.003258	6.25E-05	0.002609
Nefl	1816.5252	-0.269616407	0.068804	-3.91859	8.91E-05	0.003513
Por	206.4166	0.413488399	0.107077	3.861593	0.000113	0.00421
Abcb1a	324.32568	0.549459301	0.145429	3.778186	0.000158	0.005608
Atp1b1	3803.9302	-0.300478799	0.081066	-3.70659	0.00021	0.005966
Atp5pb	2122.1659	-0.336682774	0.0903	-3.72847	0.000193	0.005966
Pgam1	1461.2675	-0.202733952	0.054399	-3.7268	0.000194	0.005966
Pkd2	175.05778	0.444673017	0.118667	3.747236	0.000179	0.005966
Ubqln1	1019.1859	0.246185753	0.066316	3.712294	0.000205	0.005966
Phyh	798.92311	0.186430436	0.050665	3.679694	0.000234	0.006377
Atp6v1g2	637.59773	0.31742528	0.087002	3.648465	0.000264	0.006459
Clec7a	26.107458	1.087343386	0.296698	3.664809	0.000248	0.006459
Olfml3	95.59177	0.670097654	0.1833	3.655743	0.000256	0.006459
Syt4	932.59237	-0.249795302	0.068826	-3.62938	0.000284	0.006724
Ppp3r1	2683.4329	0.172608563	0.049236	3.505751	0.000455	0.010428
Abca1	262.01745	0.403300955	0.115524	3.491054	0.000481	0.010675
Lgals3	135.53315	1.239819871	0.358831	3.455164	0.00055	0.011832
Sec23a	777.46027	-0.20771643	0.060412	-3.43831	0.000585	0.012224
Stat1	137.90057	0.385176329	0.11244	3.42561	0.000613	0.012444
Fcgrt	207.05144	0.574423509	0.170395	3.37113	0.000749	0.014118
Syngr1	330.8947	-0.324931123	0.09646	-3.36856	0.000756	0.014118
Vegfa	410.08029	-0.384318905	0.114061	-3.36943	0.000753	0.014118
Btrc	365.77348	0.216304426	0.064662	3.345144	0.000822	0.014242
C4a/b	405.13742	0.548131093	0.163582	3.350796	0.000806	0.014242

Gria1	306.1003	-0.358319904	0.106742	-3.35687	0.000788	0.014242
Lyz2	224.97731	0.772299287	0.232069	3.327889	0.000875	0.014793
Pros1	166.54738	0.3649637	0.110239	3.310662	0.000931	0.015368
Bhlhe41	380.86656	0.252109344	0.076625	3.290175	0.001001	0.016157
Ccl12	40.40324	0.693194288	0.21298	3.254743	0.001135	0.016762
Gabrg2	588.4925	-0.292592947	0.090048	-3.24932	0.001157	0.016762
Gng3	3556.9006	-0.175438281	0.053861	-3.25726	0.001125	0.016762
Tlr2	30.564475	0.666300383	0.20419	3.26314	0.001102	0.016762
Tspo	48.425332	0.669733854	0.205508	3.258923	0.001118	0.016762
Eif2ak1	267.44693	0.309564323	0.096546	3.206407	0.001344	0.017354
Map2k4	931.71592	-0.181107505	0.056484	-3.20635	0.001344	0.017354
Pdgfra	175.54295	0.49442993	0.153883	3.213033	0.001313	0.017354
Pllp	304.40488	-0.272425382	0.08485	-3.21067	0.001324	0.017354
Serpini1	833.08977	-0.250031896	0.077631	-3.22076	0.001278	0.017354
Wdr35	183.81721	-0.285237923	0.08826	-3.23178	0.00123	0.017354
Gsn	2196.3538	0.758490635	0.238641	3.178374	0.001481	0.018777
Ptprz1	904.87646	-0.327049392	0.104818	-3.12018	0.001807	0.022513
Gabra3	578.87249	-0.300019597	0.096919	-3.09556	0.001964	0.024047
Slc6a1	1045.3188	-0.236514749	0.077009	-3.07128	0.002131	0.025222
Trim37	1549.7873	-0.234816975	0.076381	-3.07428	0.00211	0.025222
Gnb1	2194.9711	-0.126714403	0.04147	-3.05559	0.002246	0.025314
Mkrn1	815.6471	-0.17263674	0.056486	-3.05625	0.002241	0.025314
Vim	1101.2022	0.585702848	0.191575	3.057296	0.002233	0.025314
Epb41l2	484.04379	0.207945404	0.068646	3.029225	0.002452	0.026974
Ptgis	76.105696	0.787662751	0.260207	3.027058	0.002469	0.026974
Grk3	175.37765	-0.268505764	0.089018	-3.0163	0.002559	0.027527
Ank2	1867.5257	0.331006607	0.110537	2.994523	0.002749	0.027836
Atp5b	4297.5644	-0.209027181	0.06956	-3.00498	0.002656	0.027836
Fabp5	220.80105	-0.318324505	0.106439	-2.99067	0.002784	0.027836
Il34	165.59851	0.514122417	0.171452	2.998643	0.002712	0.027836
Pde1a	199.05232	0.462378257	0.154298	2.99666	0.00273	0.027836
Rfx4	110.02134	-0.464635531	0.155678	-2.9846	0.00284	0.028001
Ccl6	37.235432	0.839678436	0.283002	2.967037	0.003007	0.028849
Ugt8a	1117.062	-0.308145076	0.10381	-2.96836	0.002994	0.028849
Hacd3	636.35436	0.184410857	0.063121	2.92155	0.003483	0.032538
Lpl	37.932436	0.542890956	0.18567	2.923963	0.003456	0.032538
Acsl3	1627.3144	-0.233535817	0.080599	-2.8975	0.003762	0.033002
Gprasp1	1481.3259	-0.216795705	0.074763	-2.89979	0.003734	0.033002
Nrg1	337.53984	-0.273416118	0.093872	-2.91265	0.003584	0.033002
P2ry12	240.22756	-0.468836415	0.161824	-2.8972	0.003765	0.033002
Psmb8	62.66405	0.571657297	0.196941	2.902687	0.0037	0.033002
Lilrb4a	24.301745	0.76193698	0.263382	2.892893	0.003817	0.033051
Lamtor3	547.24987	-0.185545031	0.064402	-2.88106	0.003963	0.033904
Psma5	893.74978	-0.229470273	0.079809	-2.87523	0.004037	0.034125
Tln2	263.68424	0.412508176	0.143721	2.8702	0.004102	0.034265
Skp1a	2476.0575	-0.191714011	0.067214	-2.85231	0.00434	0.035832
Ube2d2a/l	714.96026	0.175291618	0.061647	2.843462	0.004463	0.036044

Ube3c	282.93088	-0.266251871	0.093648	-2.84312	0.004467	0.036044
Cd74	277.36424	1.136610972	0.401056	2.834048	0.004596	0.036667
Cnp	7982.7306	-0.258382281	0.092255	-2.80073	0.005099	0.039781
Mbp	13540.445	-0.33798953	0.120552	-2.80367	0.005052	0.039781
Adcy1	238.20363	-0.316605034	0.113398	-2.79197	0.005239	0.04043
E2f3	106.10042	-0.302057366	0.108559	-2.78243	0.005395	0.04119
Gjb1	457.73409	-0.314208624	0.114183	-2.7518	0.005927	0.044766
Dlgap1	75.939182	0.448842341	0.164163	2.734125	0.006255	0.046309
Prkacb	201.74759	0.266384401	0.097442	2.73376	0.006262	0.046309
Klk6	2062.6104	-0.314597397	0.1159	-2.71439	0.00664	0.048105
Sox10	459.38799	-0.292634345	0.107784	-2.71502	0.006627	0.048105
Mapk10	1834.3147	-0.14004828	0.051814	-2.70288	0.006874	0.049299
Iigp1	45.42719	0.808337924	0.300986	2.685631	0.007239	0.05089
Kcnk13	117.20858	-0.318259229	0.118386	-2.68831	0.007181	0.05089
Atp5g3	1932.0149	-0.137338146	0.051542	-2.66456	0.007709	0.052396
Cd34	310.84538	0.529187447	0.198731	2.66283	0.007749	0.052396
Slc9a6	1363.988	-0.130439037	0.048954	-2.66454	0.007709	0.052396
Vdac3	2471.5943	-0.249809753	0.093527	-2.67099	0.007563	0.052396
Reck	63.460223	0.652419662	0.245525	2.657239	0.007878	0.05277
Samm50	550.37979	-0.184309229	0.069662	-2.64578	0.00815	0.054081
Gbp2	30.813475	0.654027684	0.249551	2.620814	0.008772	0.057139
Sqstm1	1783.7286	0.160439049	0.061213	2.620979	0.008768	0.057139
Emp1	110.43683	0.731339684	0.279676	2.61495	0.008924	0.057273
Igfbp5	332.73685	1.129831535	0.432762	2.610743	0.009035	0.057273
Nes	79.137616	0.431715227	0.165351	2.610905	0.00903	0.057273
Pak1	1757.3826	-0.226426958	0.088123	-2.56943	0.010187	0.064004
Gpr17	72.42369	-0.407916837	0.159909	-2.55092	0.010744	0.066913
Zpr1	139.8407	-0.287235451	0.113069	-2.54037	0.011074	0.068368
Pdhb	1565.4038	-0.189169748	0.074686	-2.53288	0.011313	0.069242
Pias1	130.94332	-0.304989814	0.121902	-2.50193	0.012352	0.074956
Amigo2	70.374811	0.394594228	0.159787	2.469503	0.01353	0.080726
Icam2	37.337625	0.432850221	0.175188	2.470781	0.013482	0.080726
Bhlhe40	551.6933	0.141825784	0.057824	2.452701	0.014179	0.08282
Fos	34.933714	-0.621461702	0.253515	-2.45138	0.014231	0.08282
Pecam1	104.03817	0.293421277	0.119545	2.454488	0.014109	0.08282
Ptgs2	11.346207	0.957460705	0.393012	2.436213	0.014842	0.085673
Csnk2a1	969.40671	0.167596805	0.069022	2.42818	0.015175	0.086888
Stat3	405.11246	0.400579773	0.166202	2.410197	0.015944	0.090561
Glrb	3153.0227	-0.166586404	0.069233	-2.40616	0.016121	0.090842
Acat1	968.85297	-0.21628329	0.090455	-2.39106	0.0168	0.092281
Grn	197.22612	0.294875305	0.123579	2.386134	0.017027	0.092281
Islr2	16.902434	1.043062455	0.435372	2.395796	0.016584	0.092281
Plp1	47387.289	-0.229353476	0.096101	-2.38658	0.017006	0.092281
Sirpa	282.43444	0.222668542	0.093292	2.386793	0.016996	0.092281
Aldh1a1	729.05879	0.236535332	0.099942	2.366732	0.017946	0.096527
Olig1	169.68507	-0.294255762	0.124707	-2.35958	0.018296	0.097669
Pgk1	4876.3825	-0.156141634	0.066436	-2.35026	0.01876	0.099402

Nkiras1	824.98475	-0.197949159	0.084542	-2.34142	0.019211	0.101034
Clta	2189.2402	0.12506469	0.053588	2.333802	0.019606	0.102355
Atg12	413.41656	-0.145757863	0.062546	-2.33041	0.019785	0.102533
Nuak1	64.811932	-0.310788097	0.134221	-2.3155	0.020586	0.105911
Egfr	54.70631	0.392414618	0.169936	2.309188	0.020933	0.106925
Adamts1	143.24641	0.579380299	0.25141	2.304522	0.021193	0.107481
Serinc3	1290.2937	0.144416639	0.062791	2.299961	0.02145	0.108013
Aqp4	1973.6101	-0.220954802	0.096267	-2.29524	0.021719	0.108597
Adgra2	60.411356	0.605182292	0.264601	2.28715	0.022187	0.108635
Adora2b	102.28842	-0.357253615	0.156378	-2.28455	0.022339	0.108635
Eno2	1270.6751	-0.204908375	0.089543	-2.28838	0.022115	0.108635
Hexb	1318.8592	0.177964874	0.077726	2.289637	0.022042	0.108635
Timp2	2570.9845	0.216766879	0.095249	2.275782	0.022859	0.109819
Lars	239.42901	-0.184510429	0.081095	-2.27524	0.022892	0.109819
Aars	328.89192	-0.184094799	0.081978	-2.24567	0.024725	0.117817
Ncam2	627.85781	-0.150630369	0.067301	-2.23816	0.025211	0.11933
Rab6b	2106.5156	-0.11964888	0.053711	-2.22762	0.025906	0.121007
Syt13	93.422344	-0.270180537	0.121181	-2.22957	0.025776	0.121007
Dnm1l	918.16267	-0.10222995	0.046082	-2.21843	0.026526	0.123094
Lsr	68.45968	0.414949055	0.187396	2.214288	0.026809	0.1236
Olfm3	301.687	-0.224128834	0.101875	-2.20005	0.027804	0.127359
C1s1	28.085174	0.677258737	0.308653	2.19424	0.028218	0.128429
Fth1	116268.76	0.223940414	0.102494	2.184919	0.028895	0.130671
Ccnh	778.34957	-0.129253155	0.059504	-2.17219	0.029841	0.134055
Ggta1	38.71406	0.400911873	0.18498	2.167331	0.03021	0.134055
Ppp2ca	3262.8651	-0.108498259	0.050059	-2.16742	0.030203	0.134055
C1ra	32.926692	0.892569184	0.412841	2.162016	0.030617	0.135019
Atp2b4	207.06103	0.22779596	0.106516	2.138606	0.032468	0.141639
H2-T23	56.050954	0.436670488	0.204477	2.135543	0.032717	0.141639
Mbtps1	211.84617	0.218767014	0.102355	2.137346	0.03257	0.141639
Cacnb2	83.864175	0.284672603	0.133476	2.132757	0.032945	0.141762
Prkcq	112.09157	0.363016844	0.170915	2.123961	0.033673	0.144025
Erlec1	348.89278	-0.139394894	0.065726	-2.12086	0.033934	0.144269
Psmd6	1093.8972	-0.135298837	0.06392	-2.1167	0.034285	0.144897
Ndufa10	1219.3093	0.12408853	0.058891	2.107085	0.03511	0.147504
Atp6v1c1	1682.1772	-0.097294874	0.046318	-2.10057	0.035678	0.149009
Myrf	277.6636	-0.266600606	0.12796	-2.08346	0.037209	0.152709
Nf1	249.38678	0.207410071	0.09936	2.087462	0.036846	0.152709
Tomm20	1476.7659	-0.140154902	0.067195	-2.08581	0.036996	0.152709
Fcgr1	44.057058	0.349974526	0.168698	2.074566	0.038027	0.155167
Ckb	11981.469	-0.17490558	0.084761	-2.06351	0.039064	0.157404
Dnaja2	488.27328	0.131369333	0.063649	2.063967	0.039021	0.157404
Psmd14	1114.9068	0.097025615	0.047062	2.06166	0.03924	0.157404
Aplp2	3662.4182	0.12812184	0.062341	2.05519	0.039861	0.158345
Olig2	58.06381	-0.408394057	0.198774	-2.05457	0.039921	0.158345
Igtp	36.424239	0.533815516	0.260688	2.047715	0.040588	0.160097
Prkar1a	3079.9248	-0.073266571	0.036003	-2.03501	0.04185	0.164164

Dusp3	262.02622	-0.193452462	0.095426	-2.02725	0.042637	0.165601
Ywhaz	2401.2836	0.101036365	0.04985	2.026797	0.042683	0.165601
Cst3	12873.402	-0.205637335	0.101596	-2.02407	0.042963	0.165781
Lgmn	2332.5638	0.076936456	0.038152	2.016584	0.043739	0.167241
Slc25a4	7919.0089	-0.136269786	0.067598	-2.01588	0.043812	0.167241
Fa2h	390.50978	-0.236572519	0.117845	-2.00748	0.044698	0.169709
Tlr4	44.075327	0.469768732	0.235003	1.998992	0.045609	0.172248
Cd163	30.895105	0.658590184	0.329887	1.996413	0.045889	0.172264
Cd33	34.866171	0.429767284	0.215478	1.994487	0.046099	0.172264
Cd200	381.15777	0.171320739	0.086018	1.991683	0.046406	0.172503
Dync1li1	141.37395	0.218521933	0.110172	1.983463	0.047316	0.17497
Eid1	1294.1291	0.119752675	0.060652	1.974437	0.048332	0.176748
Htr5a	22.368085	-0.5223964	0.264791	-1.97286	0.048512	0.176748
Tspan7	1807.788	-0.12370387	0.062712	-1.97258	0.048544	0.176748
Rtn4	2366.1152	-0.089638971	0.045591	-1.96614	0.049283	0.178525
C1qc	367.64967	0.232573326	0.119552	1.945379	0.051729	0.186436
Serping1	228.25854	1.009966621	0.521369	1.937142	0.052728	0.189075
Prkcz	267.04605	-0.1788274	0.092433	-1.93467	0.053031	0.189204
Sec61a2	162.20717	-0.194787484	0.101221	-1.92437	0.054308	0.192794
Cspg4	51.869723	0.38214487	0.199737	1.913243	0.055717	0.195837
Pccb	299.76324	0.175505514	0.091666	1.914625	0.05554	0.195837
Cd14	30.459117	0.441858221	0.231484	1.908805	0.056287	0.196867
Atp6v1b2	2271.7939	0.117829008	0.061882	1.90409	0.056898	0.198029
Bdnf	66.561949	0.283584286	0.150669	1.882169	0.059813	0.207157
Mapk1	1238.9263	0.084570581	0.045085	1.875785	0.060685	0.209156
Cd84	51.449502	0.315232906	0.169155	1.863571	0.062382	0.213601
Sall3	60.668186	-0.31102803	0.167214	-1.86006	0.062877	0.213601
Slc1a3	618.14622	-0.220091733	0.11822	-1.86171	0.062644	0.213601
Epb41l3	1315.8256	-0.139522575	0.075238	-1.85441	0.06368	0.215299
Cpne2	140.75773	0.292010529	0.157928	1.849009	0.064457	0.216892
Mapk14	268.69343	0.145953439	0.079152	1.843967	0.065188	0.218318
Snap25	7624.1077	-0.105812018	0.057542	-1.83885	0.065937	0.218792
Tusc3	531.62384	-0.120003111	0.065336	-1.8367	0.066254	0.218792
Zfp365	430.10893	-0.219568664	0.119421	-1.83862	0.065972	0.218792
Atp6v1e1	1803.5455	-0.127455256	0.069618	-1.83077	0.067135	0.220676
Ctnnb1	1917.1778	-0.104109546	0.056976	-1.82727	0.06766	0.221376
Stab1	21.959226	0.540031463	0.296593	1.820781	0.06864	0.223553
Atp6v1a	1667.4748	-0.113749208	0.062752	-1.81267	0.069882	0.226559
Tbp	137.93984	-0.175832507	0.09757	-1.80211	0.071527	0.230839
Adcyap1r1	702.41721	-0.182396494	0.101546	-1.7962	0.072462	0.231748
Klrb1	8.7103191	-0.657559027	0.365909	-1.79706	0.072327	0.231748
Cx3cl1	136.07093	-0.211043993	0.118213	-1.78529	0.074214	0.236286
Srgn	248.83066	0.388876591	0.21908	1.775044	0.075891	0.240546
Slc24a3	142.81819	-0.197130235	0.111955	-1.7608	0.078272	0.246991
Ass1	178.0193	0.232072534	0.132092	1.756898	0.078935	0.247929
Csnk2a2	263.27314	0.134292246	0.076522	1.754952	0.079268	0.247929
Usp2	152.61952	0.192364492	0.109833	1.751421	0.079873	0.248729

Becn1	291.86775	-0.151551823	0.086839	-1.7452	0.08095	0.249888
Ccl2	17.354513	0.68645349	0.39331	1.745326	0.080928	0.249888
Atp6v1h	2528.898	-0.131754149	0.075727	-1.73986	0.081883	0.250591
Lamp2	1557.1282	0.149439211	0.085847	1.740765	0.081725	0.250591
Axl	313.26863	0.170845834	0.098375	1.736678	0.082444	0.251224
Cntnap1	380.90842	-0.175311607	0.101074	-1.73448	0.082832	0.251329
Agmo	267.76857	-0.266590577	0.154258	-1.72821	0.08395	0.253277
Ulk1	83.431585	0.428237734	0.247982	1.726888	0.084188	0.253277
Sall1	216.90646	-0.198281614	0.115082	-1.72295	0.084897	0.254332
Plxnb3	125.28425	-0.245347937	0.142634	-1.72013	0.08541	0.254794
Acot13	771.28825	-0.151147184	0.088916	-1.69988	0.089153	0.261564
Grm1	110.99253	-0.241718146	0.14218	-1.70008	0.089115	0.261564
Sox2	316.43085	-0.235631645	0.138411	-1.70241	0.088679	0.261564
Tpi1	217.87584	-0.138484433	0.081246	-1.7045	0.088288	0.261564
Cybb	31.56807	0.4767744	0.280867	1.697508	0.089601	0.261796
Ms4a4a	22.214749	0.504549376	0.298254	1.691678	0.090707	0.263944
P2ry1	205.15406	0.200214388	0.118971	1.682882	0.092398	0.267765
Gabra4	62.717038	-0.247068685	0.147678	-1.67303	0.094322	0.271723
Ncam1	1210.8415	-0.158490365	0.094792	-1.67198	0.094529	0.271723
S100a10	1390.2026	0.502393975	0.302579	1.660371	0.09684	0.277243
Lamp1	2177.9804	-0.078828879	0.048343	-1.63061	0.102972	0.292224
Tnfrsf21	430.077	-0.135183275	0.082913	-1.63042	0.103013	0.292224
Uchl1	781.63243	-0.134625757	0.082642	-1.62903	0.103307	0.292224
Rnf10	687.45343	0.096182665	0.059333	1.62107	0.105003	0.295841
Abcg2	274.053	0.244234676	0.151193	1.615386	0.106227	0.298108
Plekhb1	10526.893	-0.163835915	0.102561	-1.59745	0.110166	0.306738
Stxbp1	1856.7602	-0.138050977	0.086327	-1.59917	0.109784	0.306738
Igf1	37.815848	0.430857922	0.270841	1.590812	0.111652	0.308454
Il10ra	25.566443	0.32913705	0.206736	1.592063	0.111371	0.308454
Got1	2250.5217	-0.129703084	0.081734	-1.58689	0.112538	0.309582
Jak1	639.31022	0.139353526	0.087912	1.585151	0.112932	0.309582
Chil1	179.53054	-0.187455887	0.11864	-1.58004	0.114097	0.311573
C1qb	385.01065	0.208070118	0.131865	1.577907	0.114587	0.311712
Mki67	15.858865	-0.654003985	0.415383	-1.57446	0.115381	0.312674
Nrcam	543.77288	-0.117483989	0.075053	-1.56535	0.1175	0.317206
C9orf72	296.86208	-0.128881359	0.082658	-1.55922	0.118944	0.319888
Atp9a	1006.471	-0.08947677	0.058084	-1.54047	0.123446	0.323599
Camk2g	944.22008	0.116090597	0.075451	1.53863	0.123895	0.323599
Cd109	48.680383	0.502208013	0.32679	1.536793	0.124344	0.323599
Chrm2	202.69118	-0.161134437	0.104043	-1.54873	0.121447	0.323599
Cnr1	258.21739	-0.138998204	0.090067	-1.54327	0.122766	0.323599
Jak2	222.00672	-0.159465735	0.103788	-1.53646	0.124426	0.323599
Kitl	234.85531	0.157821532	0.102262	1.543299	0.122758	0.323599
Lpar6	49.386345	0.260165487	0.167807	1.550388	0.121048	0.323599
Serpine2	2066.7947	-0.161834361	0.104718	-1.54543	0.122243	0.323599
Gpm6a	3465.9162	-0.116777949	0.076267	-1.53118	0.125724	0.325782
Fcrls	69.487059	0.240319374	0.15722	1.528553	0.126375	0.326229

Kcnd1	55.894675	0.541422069	0.354617	1.52678	0.126816	0.326229
Adam22	364.46814	-0.110930048	0.072993	-1.51974	0.128577	0.328381
Gas7	316.06422	0.121125641	0.079638	1.520961	0.12827	0.328381
Gpnmb	27.872364	0.434477527	0.289801	1.499227	0.133815	0.339316
Prkci	230.53261	0.131816592	0.087908	1.499486	0.133748	0.339316
Chn1	1022.5687	-0.084968406	0.056759	-1.49702	0.134389	0.339559
Adrb1	23.443688	0.354742345	0.238835	1.485302	0.137464	0.342454
Cntnap2	183.0969	-0.156976609	0.105642	-1.48593	0.137298	0.342454
Ptprc	24.031464	0.346113399	0.232456	1.48894	0.136503	0.342454
Sod1	3114.755	-0.092143268	0.061859	-1.48957	0.136337	0.342454
Atg2b	222.79819	0.147293928	0.100018	1.472673	0.140839	0.346503
Entpd1	65.235894	0.2301774	0.156186	1.473737	0.140552	0.346503
Entpd2	79.349646	0.21533263	0.146473	1.47012	0.141529	0.346503
Galc	165.97489	-0.137930678	0.093405	-1.47669	0.139759	0.346503
Tap1	32.768175	0.321957128	0.218885	1.470898	0.141319	0.346503
Ptprm	187.53435	0.137805396	0.095083	1.449318	0.147249	0.359267
Il18	260.2459	-0.183343732	0.126881	-1.44501	0.148456	0.360972
C3ar1	46.25831	0.286212544	0.199261	1.436369	0.150897	0.365656
Map1lc3b	1131.0747	-0.065733999	0.046203	-1.42274	0.154812	0.373867
Gdpd2	77.616859	-0.184719924	0.130752	-1.41275	0.157729	0.37962
Slc2a5	31.070186	0.387808325	0.275044	1.409988	0.158543	0.380289
Slc8a1	1024.0293	-0.13149476	0.094063	-1.39795	0.162129	0.387582
Atp1a1	1189.2345	0.208553006	0.149687	1.393262	0.163541	0.38834
Unc80	446.77462	0.117993115	0.084672	1.393531	0.163459	0.38834
Cd68	92.675915	0.195675543	0.14064	1.391318	0.164129	0.388439
B2m	2078.9027	0.185500414	0.134183	1.382439	0.166837	0.390938
Esam	98.316357	0.187978629	0.135837	1.383853	0.166404	0.390938
Nmnat2	609.3624	0.098467248	0.071115	1.38461	0.166172	0.390938
Ifit1	45.812873	0.425059946	0.308272	1.378848	0.167942	0.390946
Slc46a1	31.913628	-0.259628873	0.188135	-1.38001	0.167583	0.390946
Gphn	400.9774	-0.113581725	0.082585	-1.37532	0.169031	0.392197
Acsl4	317.59984	0.13920014	0.102101	1.363353	0.172771	0.396857
Ppp3cb	1202.5065	0.0629929	0.046149	1.365	0.172253	0.396857
Rlim	574.3823	-0.102530352	0.075293	-1.36175	0.173276	0.396857
Tmem47	819.6131	0.082443518	0.06038	1.365402	0.172127	0.396857
Ube4b	255.94323	0.147774667	0.108802	1.358202	0.1744	0.398147
Atf2	279.7233	-0.097011448	0.071886	-1.34952	0.177169	0.403174
Spi1	48.28039	0.271389679	0.201742	1.345232	0.17855	0.405018
Arpc1a	1242.0607	-0.059145944	0.044426	-1.33134	0.183077	0.413965
Calm2	3346.0991	-0.117562153	0.089302	-1.31645	0.188021	0.422453
Sh3gl2	1687.4494	-0.098593568	0.07489	-1.31651	0.188004	0.422453
Ddah1	586.56734	-0.078066908	0.059898	-1.30334	0.192458	0.429703
Inpp5f	1154.7164	-0.098411618	0.075499	-1.30348	0.19241	0.429703
Chchd3	304.26052	0.090161375	0.069653	1.294432	0.195516	0.433801
Erbb3	63.131569	-0.188284249	0.145421	-1.29475	0.195406	0.433801
Arntl	40.098356	-0.21689526	0.169374	-1.28057	0.200344	0.437591
Chrnb2	59.402052	-0.196115638	0.153344	-1.27893	0.200922	0.437591

Cltc	4364.0028	0.089479375	0.069786	1.282201	0.199772	0.437591
Fgf13	561.76155	0.390556972	0.304932	1.280801	0.200264	0.437591
Myct1	28.235996	-0.254228459	0.19749	-1.2873	0.19799	0.437591
Slc9b2	52.598459	0.211384283	0.165194	1.279616	0.20068	0.437591
Gnas	3453.1267	-0.080349171	0.063367	-1.268	0.204798	0.444668
Adgrg1	61.870318	-0.173612082	0.137909	-1.25888	0.208072	0.4504
Gjd2	88.578097	-0.160730012	0.128213	-1.25362	0.20998	0.453149
Adh5	772.53972	-0.098498423	0.079115	-1.245	0.213131	0.45725
Ccnb1	13.644069	-0.439257421	0.352995	-1.24437	0.213362	0.45725
Vdac1	2082.5514	-0.056884676	0.045759	-1.24315	0.213813	0.45725
P2rx7	81.070373	0.145735709	0.117401	1.241352	0.214476	0.457291
Arl6ip5	865.96547	0.079959434	0.064763	1.234641	0.216964	0.459999
Tgfb1	71.7533	0.171880743	0.139239	1.234432	0.217042	0.459999
Rad23b	643.95341	-0.062040192	0.050679	-1.22419	0.220882	0.466744
Itga7	37.869042	0.342562025	0.28063	1.220688	0.222204	0.468145
Atp2a2	2603.5048	0.118211235	0.097722	1.209664	0.226408	0.47559
Ccr5	54.920035	0.210092092	0.173992	1.207482	0.227247	0.475944
Shc3	271.4527	-0.09746346	0.081356	-1.19798	0.230924	0.482223
Casp3	79.528939	0.164024996	0.137688	1.191283	0.233542	0.483426
Opcml	276.23361	0.109195895	0.091497	1.193433	0.2327	0.483426
Trim2	951.31427	0.071269788	0.05977	1.192408	0.233101	0.483426
Ubqln2	719.4773	0.09545224	0.080247	1.189485	0.234249	0.483479
Orc5	231.11782	-0.111558984	0.094269	-1.18342	0.236644	0.487007
Pmp22	637.66648	0.214483951	0.182687	1.174052	0.240374	0.491832
Th	20.333767	0.501700043	0.426733	1.175677	0.239724	0.491832
Gas6	729.80367	0.086273688	0.073768	1.169527	0.242191	0.49271
Slc17a7	22.078828	0.433057043	0.369782	1.171114	0.241553	0.49271
Wasf1	424.45175	0.089487394	0.076876	1.164052	0.244403	0.495789
Crkl	170.83542	-0.1178769	0.101489	-1.16147	0.24545	0.496495
Ywhah	3618.5556	0.082282085	0.071621	1.148854	0.250616	0.505504
Slc17a6	471.08566	-0.089653331	0.079364	-1.12965	0.258624	0.520178
Gria3	744.7284	-0.091798546	0.08171	-1.12347	0.261239	0.523954
Gdnf	13.506538	0.399449279	0.356931	1.119122	0.263088	0.526176
C3	403.0466	-0.127128256	0.114005	-1.11511	0.264804	0.528121
Lta4h	233.48507	0.084041402	0.075923	1.106932	0.268323	0.53364
Prkab2	72.977921	0.166514596	0.150725	1.104761	0.269263	0.534014
Akt3	1023.465	0.069674926	0.063474	1.097696	0.272337	0.538606
Cdk7	379.20177	-0.075420959	0.06897	-1.09354	0.274158	0.5407
Atp6v1d	1304.8434	-0.069838524	0.064623	-1.08071	0.279828	0.547323
Mapt	571.33167	-0.090201212	0.08341	-1.08143	0.279508	0.547323
Tlr7	11.648004	0.336704522	0.311325	1.081521	0.279466	0.547323
Myorg	100.10641	-0.125394186	0.116396	-1.07731	0.281342	0.548772
Fbln5	73.846985	0.288657172	0.269792	1.069924	0.284653	0.552196
Ccdc127	500.90594	0.062674533	0.058574	1.070013	0.284613	0.552196
Grin3a	365.78191	-0.081676569	0.076995	-1.06081	0.288777	0.558669
Itgam	81.974988	-0.131956616	0.124809	-1.05727	0.290387	0.560258
Nap1l2	236.51399	0.10639881	0.100812	1.055421	0.291233	0.560367

Psen1	283.37535	0.08040245	0.07634	1.053213	0.292244	0.560792
Klrd1	9.7585835	0.376445349	0.358383	1.050398	0.293535	0.561752
Fam104a	219.38246	-0.092786242	0.088764	-1.04531	0.295878	0.564714
Cux2	80.447927	-0.143957763	0.138227	-1.04146	0.297664	0.565084
Rasgrp1	94.868524	0.491875048	0.472185	1.041699	0.297551	0.565084
Tardbp	1510.2316	-0.05318331	0.05122	-1.03834	0.299114	0.566322
Clcf1	14.488227	-0.317349849	0.307615	-1.03165	0.302238	0.570716
Gad2	581.00426	0.076779238	0.074823	1.026148	0.304822	0.574067
Crot	307.24648	-0.086232942	0.084654	-1.01866	0.308366	0.576157
Cttnbp2nl	51.485744	-0.155828968	0.152703	-1.02047	0.307505	0.576157
Pik3r1	791.75533	0.058148354	0.057012	1.019927	0.307763	0.576157
Adora3	9.1204696	0.379390013	0.374923	1.011915	0.311579	0.577877
Ahcyl1	2065.7626	-0.066993635	0.066493	-1.00753	0.31368	0.577877
B4galt5	151.6804	0.101357642	0.100432	1.009214	0.312872	0.577877
F11r	90.498384	0.152357406	0.150436	1.012771	0.31117	0.577877
Tnfrsf11a	41.395273	0.20979932	0.208793	1.00482	0.314984	0.577877
Trim45	47.35778	-0.167687627	0.166612	-1.00646	0.314197	0.577877
Ywhag	3671.3352	0.077185454	0.076382	1.010512	0.31225	0.577877
Ap3m2	284.00783	-0.107574264	0.108266	-0.99361	0.320414	0.583317
Gpr34	119.72703	-0.13922016	0.13995	-0.99479	0.31984	0.583317
Stmn1	1903.1521	0.055166364	0.055386	0.99604	0.319231	0.583317
Dnajc5	1258.4175	0.04836338	0.049007	0.986863	0.32371	0.584529
Hacd2	359.67499	-0.075836023	0.077345	-0.98049	0.326842	0.584529
Prkcd	193.09079	0.438283664	0.445737	0.983279	0.32547	0.584529
Tie1	50.263296	0.171074804	0.174379	0.981051	0.326568	0.584529
Tomm70a	1701.6587	-0.041304301	0.04187	-0.98648	0.323897	0.584529
Supt7l	74.309767	0.126126239	0.128091	0.984663	0.32479	0.584529
Xpnpep1	276.18133	0.071590256	0.072324	0.989852	0.322246	0.584529
Fbxw11	1103.057	0.043694956	0.04478	0.975767	0.32918	0.58723
Calm1	6763.0332	-0.045644855	0.046961	-0.97197	0.331064	0.589111
Bub3	483.15423	0.055045954	0.057521	0.956975	0.33858	0.600979
Dlat	707.0363	0.048254452	0.05064	0.952886	0.340648	0.603142
Gad1	404.88838	-0.096630386	0.103665	-0.93214	0.351262	0.614379
Hmgb1	1313.374	0.078521014	0.084143	0.933187	0.350723	0.614379
Rtn1	1412.3393	0.059851285	0.064216	0.932031	0.351321	0.614379
Scp2	250.17708	-0.156266135	0.1673	-0.93405	0.350278	0.614379
Sparc	5084.5977	-0.074059879	0.078981	-0.93769	0.348405	0.614379
Fgf2	18.427182	0.25349841	0.272922	0.928832	0.352976	0.615757
Tiam1	102.7806	0.110791954	0.119504	0.927098	0.353876	0.615813
Slc12a5	528.24957	-0.120858567	0.130835	-0.92375	0.355618	0.617332
Ctsf	527.63322	-0.060326848	0.065469	-0.92146	0.356812	0.6175
Gpr84	9.3249375	0.331105225	0.359808	0.920227	0.357454	0.6175
Cnot10	179.36921	0.089356613	0.097854	0.913167	0.361155	0.622378
C1qa	532.49622	0.101406132	0.111295	0.911144	0.36222	0.622702
Adcy5	311.44768	-0.087136984	0.096879	-0.89944	0.368416	0.631048
Grm2	16.612677	-0.227738604	0.25343	-0.89863	0.368852	0.631048
Atp8a2	486.31453	0.11596006	0.129315	0.896725	0.369866	0.631261

Sugt1	878.36394	0.058711515	0.065983	0.889802	0.373572	0.636058
Ccl3	14.89651	0.306422762	0.348013	0.880492	0.378593	0.641201
Nlk	110.48876	-0.107308672	0.122055	-0.87918	0.379302	0.641201
Ranbp9	620.40072	-0.063743367	0.0723	-0.88165	0.377968	0.641201
Csf1r	309.06403	0.092206724	0.105533	0.873726	0.382267	0.643794
Klhl12	194.15772	0.094041444	0.108131	0.869703	0.384463	0.643794
Pik3cb	387.90361	-0.073869597	0.084662	-0.87253	0.38292	0.643794
Unc79	746.80144	-0.08498277	0.097602	-0.87071	0.383914	0.643794
Ap2m1	2552.9052	0.034239569	0.039453	0.867855	0.385474	0.643968
Ptk2b	32.927528	0.182431584	0.211207	0.863759	0.38772	0.646201
Gabarapl1	3705.7892	0.032774754	0.038465	0.852057	0.394183	0.653901
Sirt5	88.381293	-0.107849693	0.126379	-0.85338	0.393446	0.653901
Map2k1	1078.9371	-0.041778735	0.049537	-0.84338	0.399014	0.660373
Ifngr2	219.54103	-0.068560844	0.081974	-0.83637	0.402947	0.66533
Cyfip2	1490.388	0.065197806	0.07844	0.831176	0.405874	0.667061
Sumo1	1018.132	-0.093971557	0.112884	-0.83246	0.405149	0.667061
Cacna2d1	157.68809	0.100033474	0.121168	0.825579	0.409043	0.669172
Txn1	2829.3565	0.195745177	0.237032	0.825816	0.408909	0.669172
Elmo1	431.35231	-0.067815549	0.082582	-0.82119	0.411536	0.671703
Itm2a	771.43824	0.093549942	0.115013	0.813383	0.415999	0.677429
Diaph1	238.75761	0.069657767	0.085931	0.810623	0.417582	0.678452
P2ry14	19.328508	0.210475607	0.260686	0.807392	0.41944	0.679915
Irf8	15.648166	0.256212264	0.319221	0.802617	0.422196	0.681271
Nckap1	1828.3386	-0.030910321	0.038472	-0.80346	0.42171	0.681271
Sptan1	1346.3281	-0.073398562	0.092385	-0.79449	0.426912	0.687319
Opa1	471.77786	0.051655257	0.065203	0.792227	0.428228	0.687878
Atrn	585.77892	-0.066038683	0.083545	-0.79046	0.429261	0.687981
Gabrd	13.405628	-0.245994403	0.312922	-0.78612	0.431797	0.690486
Egf	11.67677	0.257442227	0.331823	0.775842	0.437842	0.693902
Immt	828.5392	0.03794429	0.048875	0.77635	0.437542	0.693902
Plaa	153.07576	-0.088495554	0.113692	-0.77838	0.436344	0.693902
Slc38a1	676.14034	-0.065127784	0.08366	-0.77848	0.436287	0.693902
Psmc6	1703.8204	0.067862407	0.087833	0.772627	0.439743	0.695362
Cdk5r1	98.556084	0.088567673	0.115392	0.767535	0.442763	0.697994
Fcgr4	9.6454456	0.276715313	0.361007	0.766509	0.443373	0.697994
Tjp1	554.02696	0.046218013	0.060467	0.764345	0.444662	0.698473
Bax	108.26943	-0.091189439	0.119747	-0.76152	0.446348	0.699574
Ywhae	6382.8822	0.024809801	0.032686	0.75904	0.447829	0.700349
Mertk	52.238162	-0.128927091	0.170938	-0.75424	0.450708	0.703303
Grb2	470.35202	0.047087112	0.06275	0.750395	0.453017	0.705355
Kcnab1	656.67357	-0.049746206	0.066903	-0.74355	0.457147	0.710229
Adar	260.29796	-0.07154351	0.09674	-0.73955	0.459574	0.712441
Adgrl4	92.29833	0.085160805	0.119157	0.714695	0.474798	0.715844
Cacna1b	62.686867	0.119884606	0.167181	0.717094	0.473316	0.715844
Ctsl	297.33677	0.135016041	0.187337	0.720713	0.471086	0.715844
Dnajb9	498.15449	0.058937594	0.082589	0.713622	0.475461	0.715844
Gla	134.36962	-0.08564764	0.119617	-0.71602	0.47398	0.715844

Gria4	759.18383	-0.057301993	0.078385	-0.73104	0.464757	0.715844
Ip6k2	276.8593	-0.051966202	0.072746	-0.71435	0.475012	0.715844
Jun	249.98999	0.087445229	0.122443	0.714173	0.47512	0.715844
Pdpk1	356.71687	0.052516864	0.07217	0.727684	0.466807	0.715844
Rasa1	160.86845	0.063056263	0.088373	0.713524	0.475522	0.715844
Shank2	29.951246	-0.142333895	0.196699	-0.72361	0.469304	0.715844
Sox9	136.07944	-0.08082267	0.113366	-0.71294	0.475885	0.715844
Spp1	1468.9808	-0.087162972	0.121245	-0.7189	0.472204	0.715844
Tsc1	171.71827	-0.062162992	0.085935	-0.72337	0.469451	0.715844
Nlrp3	11.406416	0.287794021	0.406557	0.707882	0.479019	0.719034
Braf	263.02753	-0.060307314	0.085969	-0.7015	0.482993	0.721947
Ppt1	758.55646	0.051837332	0.073764	0.702743	0.482216	0.721947
Cd9	938.10246	0.113619757	0.162926	0.69737	0.485571	0.724277
Ret	197.71745	0.223179044	0.323611	0.689652	0.490413	0.729965
Arpc2	668.85322	-0.04179472	0.063334	-0.65991	0.509314	0.737986
Arpc5l	687.38878	-0.052129203	0.078539	-0.66374	0.506859	0.737986
Camk2d	757.92228	0.057773477	0.086716	0.66624	0.505258	0.737986
Cd6	8.8902869	0.253344098	0.377966	0.670283	0.502677	0.737986
Dnm3	1440.0367	-0.048146509	0.07262	-0.66299	0.507335	0.737986
Pcca	306.35028	0.053837001	0.080725	0.666918	0.504825	0.737986
Prkce	391.79072	0.06250401	0.093033	0.671851	0.501679	0.737986
Ptx3	15.979106	-0.185723924	0.277229	-0.66993	0.502903	0.737986
Rab1a	2294.3156	-0.031710132	0.04792	-0.66174	0.50814	0.737986
S100b	6177.7358	-0.066627907	0.100941	-0.66007	0.50921	0.737986
Slc25a17	298.65483	-0.058064664	0.08798	-0.65997	0.509272	0.737986
Trim68	30.707596	-0.139609623	0.208263	-0.67035	0.502633	0.737986
Mto1	169.94877	-0.068211924	0.100778	-0.67686	0.498498	0.737986
Stambpl1	161.51934	0.063268283	0.096496	0.655655	0.512046	0.740433
Asns	366.5451	0.061049062	0.094343	0.647095	0.51757	0.7469
Atr	102.6631	0.075053332	0.11685	0.642306	0.520675	0.749854
Lrrc7	116.43134	0.103517534	0.161985	0.639058	0.522785	0.749854
Rims1	237.48067	-0.066771257	0.104295	-0.64022	0.522032	0.749854
Hsd17b4	337.81239	-0.047107602	0.074595	-0.63151	0.527708	0.755388
Ank	995.53838	0.045822573	0.073238	0.625667	0.531534	0.75629
Cx3cr1	135.1377	-0.08988142	0.143386	-0.62685	0.530759	0.75629
Sgce	488.81283	0.041309688	0.065738	0.628402	0.529741	0.75629
Rorb	155.99508	-0.062450914	0.10098	-0.61845	0.53628	0.761517
Syn1	1376.329	0.055826165	0.090988	0.613557	0.539508	0.764572
Mag	966.47174	-0.07119942	0.116669	-0.61027	0.541685	0.766128
Dnm1	2567.5656	0.062938358	0.104617	0.601607	0.547435	0.769662
Kcnd2	92.614105	-0.086834554	0.143577	-0.60479	0.545316	0.769662
Ppp2r2b	416.62424	-0.046305214	0.076823	-0.60275	0.546675	0.769662
B4galt6	550.36448	0.050012591	0.083488	0.599043	0.549144	0.770539
Hsd11b1	301.43554	0.085702444	0.143761	0.596144	0.551079	0.771382
Nwd1	111.10499	-0.063281927	0.10653	-0.59403	0.552492	0.771382
Slc12a2	1010.5918	-0.03643366	0.061581	-0.59164	0.554091	0.771382
Gusb	88.789657	0.070203786	0.118588	0.591999	0.553851	0.771382

Mtor	155.75203	-0.072429281	0.123182	-0.58798	0.556543	0.773279
Khsrp	112.80558	0.06299769	0.107941	0.583632	0.559468	0.775125
Tmem88b	10.737617	-0.213841226	0.366946	-0.58276	0.560055	0.775125
Dock3	444.15604	0.064833704	0.111652	0.580677	0.561458	0.775555
Gmpr2	131.51208	-0.056514708	0.097923	-0.57713	0.563851	0.775841
Srpr	536.49985	0.04578978	0.079264	0.577686	0.563476	0.775841
Lrrc25	8.3728482	0.250558304	0.435558	0.575259	0.565116	0.776079
Cpa3	10.70594	0.313137214	0.550887	0.568424	0.569747	0.780928
Trem2	55.738335	0.117905973	0.20955	0.562663	0.573664	0.784781
Slco2b1	20.304952	-0.140973327	0.254547	-0.55382	0.579702	0.791516
Prkab1	99.99394	0.06527171	0.118304	0.551727	0.581135	0.79195
Olfr110	9.7620675	-0.204153013	0.371384	-0.54971	0.58252	0.792316
Acly	967.02202	-0.050558167	0.095537	-0.5292	0.596668	0.799403
Atg5	306.43589	-0.040236795	0.074944	-0.53689	0.59134	0.799403
Atp2b3	154.84596	0.077810986	0.145631	0.534301	0.593133	0.799403
Bcas1	2777.8379	0.054301272	0.103238	0.525979	0.598903	0.799403
Cacna1e	75.419069	0.086222092	0.162995	0.528987	0.596815	0.799403
Glra3	91.904261	-0.068661874	0.130572	-0.52585	0.598989	0.799403
Hdc	10.411562	-0.18217905	0.338712	-0.53786	0.590675	0.799403
Ninj2	33.903269	-0.132125678	0.247434	-0.53398	0.593353	0.799403
Pnoc	21.255328	-0.129245834	0.243057	-0.53175	0.594899	0.799403
Ppm1a	1644.1967	-0.021113992	0.039848	-0.52987	0.596202	0.799403
Cacna2d3	83.493791	0.062719348	0.121562	0.515944	0.605893	0.805588
Lag3	25.795028	0.120459352	0.233417	0.51607	0.605806	0.805588
Psmc2	1133.7106	-0.021949123	0.042945	-0.5111	0.609283	0.808581
Abce1	463.01363	0.037132791	0.074223	0.500285	0.616875	0.813588
Akt1	717.23953	-0.026814131	0.053538	-0.50084	0.616481	0.813588
Amph	653.82092	0.033839879	0.069744	0.4852	0.627534	0.813588
Bin1	1464.8422	-0.041674034	0.084182	-0.49504	0.620568	0.813588
Cdc42	4084.9566	0.022920368	0.047296	0.484611	0.627952	0.813588
Cdc7	28.542535	0.113164795	0.23234	0.487066	0.626212	0.813588
Dclk1	461.68727	-0.043161252	0.08827	-0.48897	0.624865	0.813588
Fpr1	12.130213	0.163977293	0.331005	0.495392	0.620323	0.813588
Il21r	8.2639436	0.180686916	0.369655	0.488799	0.624984	0.813588
Kctd12	20.794485	0.120535834	0.248582	0.484894	0.627752	0.813588
Nefh	4160.0468	0.053718303	0.108788	0.49379	0.621454	0.813588
Ptk2	256.99755	0.040164566	0.080807	0.497043	0.619158	0.813588
Serpina3n	166.2574	-0.067438897	0.136957	-0.49241	0.622431	0.813588
Dld	1267.2552	-0.038353835	0.080038	-0.47919	0.6318	0.815418
Mapk8	447.90868	0.028604502	0.05987	0.477775	0.632811	0.815418
P2ry13	37.470781	0.095399505	0.199009	0.479373	0.631674	0.815418
Trim17	22.386348	0.116223294	0.247816	0.46899	0.639077	0.822001
Camk2a	211.07328	0.065396696	0.140044	0.466972	0.64052	0.822367
Anapc7	26.027923	-0.097878773	0.212197	-0.46126	0.644609	0.823152
Hpgds	47.150716	-0.076971297	0.166583	-0.46206	0.644039	0.823152
Kcnq5	13.212504	0.144877336	0.312548	0.463536	0.64298	0.823152
Kras	612.48342	-0.026418903	0.058269	-0.4534	0.650264	0.825916

Mad2l1	17.746811	0.123671536	0.272385	0.454032	0.649805	0.825916
Parp2	64.349857	0.072163128	0.158847	0.454294	0.649617	0.825916
Xk	85.907462	0.061847446	0.137694	0.449165	0.653313	0.828307
Dnajc11	141.70164	-0.045770805	0.10435	-0.43863	0.660931	0.836472
Mef2a	284.29318	0.039150381	0.090261	0.433748	0.664471	0.839456
Fig4	201.76996	-0.042710323	0.099999	-0.42711	0.669302	0.839638
Nsf	4657.4799	0.029416245	0.068883	0.427046	0.669345	0.839638
P2ry6	13.408132	0.131807736	0.307898	0.428089	0.668586	0.839638
Pmepa1	100.77592	0.065795024	0.153663	0.428178	0.668522	0.839638
Sybu	123.72163	0.044408981	0.105138	0.422389	0.672741	0.842409
Smad3	40.301699	0.078732795	0.188157	0.418442	0.675624	0.843046
Trim35	122.1047	0.041609151	0.099078	0.419962	0.674513	0.843046
Tyrobp	386.26507	0.065533528	0.160473	0.408376	0.682997	0.850751
Syt1	584.29243	0.055782523	0.137614	0.405354	0.685217	0.852022
Cables1	165.63238	0.043948308	0.108989	0.403236	0.686774	0.852172
Csf1	311.64726	-0.033319137	0.083236	-0.4003	0.688939	0.852172
Ticam1	30.749204	0.085419557	0.21271	0.401578	0.687995	0.852172
Camk4	95.738741	-0.058412502	0.148881	-0.39234	0.694803	0.852192
Cysltr1	20.967428	0.095619241	0.24019	0.398098	0.690558	0.852192
Grm7	257.09253	0.04850156	0.124199	0.390513	0.696157	0.852192
Scn1a	1230.3765	-0.035322447	0.090354	-0.39093	0.695847	0.852192
Siglech	96.360288	0.050341501	0.128498	0.391768	0.69523	0.852192
Tbk1	135.63174	0.040205348	0.101984	0.394233	0.693409	0.852192
Plcb4	432.11505	0.0286688	0.074389	0.38539	0.699949	0.855359
Eloc	2112.7816	-0.028598567	0.074755	-0.38257	0.702042	0.856443
Calm3	4748.8447	-0.022854483	0.060478	-0.3779	0.705506	0.857721
Orc3	433.2634	-0.027256952	0.071947	-0.37885	0.704799	0.857721
Cst7	10.585636	-0.163631204	0.437602	-0.37393	0.708459	0.859839
Map3k4	166.92307	0.042582877	0.115567	0.368469	0.712524	0.863297
Mal2	395.62964	0.100814528	0.275052	0.366528	0.713971	0.863576
Abcc8	19.407596	0.091885891	0.259079	0.354663	0.722842	0.864322
Acadm	309.81206	-0.034161308	0.097809	-0.34927	0.726889	0.864322
Cdc27	340.80099	0.025726283	0.071923	0.357691	0.720574	0.864322
Cep68	53.267593	-0.053334588	0.146538	-0.36396	0.715885	0.864322
Chrm3	31.215306	-0.068114058	0.195304	-0.34876	0.72727	0.864322
Cyp2u1	112.59943	0.054406101	0.155551	0.349764	0.726516	0.864322
Nrxn1	489.14936	0.022797358	0.063477	0.359142	0.719489	0.864322
Orc4	429.83232	0.024457917	0.070425	0.347292	0.728372	0.864322
Rasgrf1	301.64629	-0.030848009	0.089106	-0.3462	0.729196	0.864322
Trim46	167.64769	-0.034079786	0.094766	-0.35962	0.719131	0.864322
Ube2g1	422.63567	0.026610846	0.076796	0.346513	0.728957	0.864322
Tada2b	136.84257	-0.040971935	0.114802	-0.35689	0.721174	0.864322
Cyp4x1	69.084772	-0.048296097	0.142355	-0.33927	0.73441	0.867606
Trac	43.380537	-0.067226049	0.197223	-0.34086	0.733207	0.867606
Alas1	144.30141	-0.032142013	0.096017	-0.33475	0.737811	0.867755
Bace1	340.19088	-0.030258934	0.090532	-0.33423	0.738202	0.867755
Nptn	2791.9265	0.018484083	0.055191	0.33491	0.737693	0.867755

Tmsb4x	26484.335	0.030113186	0.091361	0.329608	0.741696	0.87042
Slc25a12	695.61521	0.025708535	0.079361	0.323942	0.745982	0.874005
Hcn1	248.85772	0.036490112	0.115508	0.31591	0.752071	0.879687
Amigo1	342.1066	-0.030161987	0.099512	-0.3031	0.761816	0.886703
Map2	1908.055	-0.025994021	0.085561	-0.30381	0.761274	0.886703
Rbx1	1535.0586	0.024858103	0.081686	0.304313	0.76089	0.886703
Selenos	142.33976	0.029489365	0.100943	0.292138	0.770181	0.894601
Sphk1	11.524802	-0.104044145	0.357651	-0.29091	0.771121	0.894601
Rab3a	6244.6285	-0.017959356	0.062132	-0.28905	0.772542	0.894788
Rapgef2	120.0585	0.032413486	0.11377	0.284905	0.775717	0.897002
Ctsb	2057.8945	0.020038287	0.070817	0.282957	0.77721	0.897267
Itpr1	192.41712	-0.027492607	0.098043	-0.28041	0.77916	0.898057
Atp6v1g1	362.79327	0.018114143	0.066302	0.273207	0.784694	0.902971
Tanc2	322.6376	-0.028107891	0.104203	-0.26974	0.787359	0.904571
Cul2	515.02157	0.014559222	0.055049	0.264477	0.791412	0.906295
Etv5	114.23014	0.02933736	0.110658	0.265116	0.79092	0.906295
Rnasel	57.13087	-0.037653337	0.147288	-0.25564	0.798225	0.911158
Rxfp1	17.51558	0.075240082	0.294228	0.25572	0.798167	0.911158
Fbxl3	531.76986	-0.021500073	0.089323	-0.2407	0.809787	0.921135
Ppm1l	175.1986	-0.027714674	0.115321	-0.24033	0.810078	0.921135
Thy1	2316.275	0.013416182	0.05606	0.239319	0.810858	0.921135
Grin2a	48.221727	0.040078426	0.170904	0.234508	0.81459	0.923896
Egr1	126.76812	-0.034237963	0.153798	-0.22262	0.823834	0.9315
Gabrb3	537.3898	-0.017872053	0.080332	-0.22248	0.823942	0.9315
Rock2	431.60523	-0.01307964	0.059687	-0.21914	0.826543	0.9315
Tmem119	26.808998	-0.045312897	0.206769	-0.21915	0.826535	0.9315
Abi2	395.45772	0.015500027	0.074041	0.209343	0.83418	0.933307
Gsk3b	825.32294	-0.013816651	0.067304	-0.20529	0.837347	0.933307
Hadh	255.14138	0.016723797	0.079701	0.209832	0.833799	0.933307
Kcnq3	192.18046	0.020209016	0.094534	0.213776	0.830722	0.933307
Reep5	984.90988	0.018335782	0.088977	0.206074	0.836733	0.933307
Scn8a	300.73256	0.02880984	0.136725	0.210714	0.833111	0.933307
Tgfbr1	113.63254	0.032894085	0.15881	0.207129	0.835909	0.933307
Ctsd	4066.4475	-0.011845145	0.058395	-0.20284	0.839257	0.933969
Mtmr4	211.42371	-0.02215805	0.111187	-0.19929	0.842039	0.935599
Mapk9	856.31705	-0.012071881	0.062189	-0.19412	0.846086	0.938626
Aldh1l1	302.12326	-0.015086143	0.079897	-0.18882	0.850234	0.938827
Hmox1	50.381591	-0.034729714	0.181501	-0.19135	0.848254	0.938827
Tm4sf1	245.4596	0.020567823	0.108807	0.18903	0.850069	0.938827
Slpi	8.8353417	-0.078135121	0.424192	-0.1842	0.853858	0.941366
Extl2	620.61135	-0.012168003	0.069662	-0.17467	0.861337	0.947825
Gabra1	447.85904	-0.012870322	0.074251	-0.17334	0.862387	0.947825
Meaf6	281.15654	-0.014646309	0.08538	-0.17154	0.863797	0.947907
Acadsb	414.75776	-0.012631404	0.07907	-0.15975	0.873079	0.949107
Anapc1	359.56621	-0.011715065	0.072314	-0.162	0.871304	0.949107
App	7432.9788	-0.01130977	0.069029	-0.16384	0.869856	0.949107
Dmd	125.34646	0.016656986	0.103317	0.161222	0.871918	0.949107

Gls	981.37973	-0.00896828	0.054425	-0.16478	0.869116	0.949107
Mafb	26.500363	0.034042082	0.215096	0.158265	0.874248	0.949107
Ncf1	43.854322	-0.033905834	0.206558	-0.16415	0.869616	0.949107
Gabra5	272.65661	-0.014470894	0.094497	-0.15314	0.87829	0.950589
Gria2	276.99037	-0.013438834	0.08709	-0.15431	0.877366	0.950589
Aif1	169.11778	-0.020243962	0.135447	-0.14946	0.88119	0.951535
Grik2	182.45942	0.013113498	0.088229	0.148631	0.881845	0.951535
Lair1	20.808193	-0.038339171	0.282002	-0.13595	0.891858	0.960879
Psmd10	202.05733	-0.014963887	0.11263	-0.13286	0.894305	0.962055
Flt1	64.377477	0.020261382	0.155902	0.129962	0.896596	0.962653
Scoc	2281.433	0.007295317	0.056722	0.128616	0.897661	0.962653
Ube2n	1800.0546	0.005877455	0.046273	0.127016	0.898928	0.962653
Eif2b5	310.15911	-0.00839788	0.067294	-0.12479	0.900687	0.963084
Adora1	85.90545	0.013693612	0.118009	0.116039	0.907622	0.96904
Ermn	2467.4651	0.014085163	0.124469	0.113162	0.909902	0.970016
Emcn	69.460818	-0.014284134	0.135408	-0.10549	0.915988	0.97447
Gfap	8089.4889	0.012844068	0.12439	0.103257	0.917759	0.97447
Hdac2	612.53944	0.006476627	0.063061	0.102704	0.918198	0.97447
Nos1ap	87.503651	-0.013440629	0.135427	-0.09925	0.920943	0.975924
Glul	8642.9847	0.006347148	0.065666	0.096659	0.922997	0.976081
Ngf	16.725144	-0.026387576	0.276467	-0.09545	0.923961	0.976081
Ptpn1	176.50431	0.00822066	0.088619	0.092764	0.926091	0.976081
Thrb	108.55659	0.011892913	0.12908	0.092136	0.92659	0.976081
Apoe	4339.0118	-0.004794526	0.064551	-0.07428	0.940791	0.976552
Cldn5	243.00479	0.008894051	0.116369	0.07643	0.939077	0.976552
Ctss	954.23098	-0.00968776	0.11756	-0.08241	0.934323	0.976552
Dagla	88.959313	0.009113291	0.116438	0.078267	0.937616	0.976552
Ina	1566.7141	-0.008238287	0.094279	-0.08738	0.930368	0.976552
Nostrin	38.368567	0.014099349	0.17911	0.078719	0.937256	0.976552
Plxdc2	512.64883	0.005246228	0.063875	0.082133	0.934541	0.976552
Prkag1	717.40915	-0.00438753	0.058995	-0.07437	0.940715	0.976552
Tmem100	24.675878	0.023177163	0.274098	0.084558	0.932613	0.976552
Asb10	9.4701947	0.027946866	0.359703	0.077694	0.938071	0.976552
Csf3r	22.391419	-0.016582677	0.2794	-0.05935	0.952672	0.980286
Dlx1	13.940575	-0.019006407	0.298047	-0.06377	0.949153	0.980286
Myo5a	808.52552	0.004532863	0.076016	0.05963	0.95245	0.980286
Panx1	107.47188	-0.009642325	0.149384	-0.06455	0.948535	0.980286
Prkdc	116.2049	-0.007972826	0.117257	-0.06799	0.94579	0.980286
Trim3	449.90489	-0.005220117	0.084097	-0.06207	0.950505	0.980286
Ifnar1	253.35911	0.004413561	0.081081	0.054434	0.956589	0.982892
Atp6v0d1	1633.6634	-0.002457178	0.054498	-0.04509	0.964038	0.984844
Cntf	250.76974	-0.006741525	0.149424	-0.04512	0.964014	0.984844
Cul1	1022.767	0.002472225	0.053835	0.045922	0.963372	0.984844
Hspa1a/b	28.860718	0.013087003	0.287943	0.04545	0.963749	0.984844
Basp1	423.1933	-0.002612396	0.060974	-0.04284	0.965826	0.985253
Drd1	14.854509	-0.010876413	0.286565	-0.03795	0.969724	0.985636
Kcnv1	22.39655	-0.010087142	0.250369	-0.04029	0.967863	0.985636

Tenm4	131.29496	0.004392544	0.118237	0.03715	0.970365	0.985636
Arg2	12.644965	0.010613851	0.316838	0.033499	0.973276	0.987098
Stat2	86.056359	-0.005093895	0.159896	-0.03186	0.974586	0.987098
Slc7a8	33.102962	0.005139647	0.190926	0.02692	0.978524	0.988321
Syn2	953.40124	0.002479407	0.092332	0.026853	0.978577	0.988321
Hk1	976.26845	0.001932366	0.085615	0.02257	0.981993	0.990362
Cdc23	224.38437	0.000885773	0.084284	0.010509	0.991615	0.995823
Gng2	734.44714	-0.001122317	0.097697	-0.01149	0.990834	0.995823
Hspa9	1567.5962	-0.000607238	0.04539	-0.01338	0.989326	0.995823
Npas2	69.874826	-0.000991335	0.141379	-0.00701	0.994405	0.997214
Atp2b2	1175.3119	0.000242365	0.115933	0.002091	0.998332	0.999025
Hectd1	469.4767	-8.78E-05	0.071897	-0.00122	0.999025	0.999025
Abcc3	7.2662786	-0.007424158	0.394963	-0.0188	0.985003	NA
B3gnt5	6.3384825	0.21230075	0.457517	0.464028	0.642627	NA
Blk	8.1214082	-0.269448496	0.394669	-0.68272	0.494784	NA
Ccna1	5.2193066	-0.156431264	0.501127	-0.31216	0.75492	NA
Cd19	7.3089171	0.08619673	0.389746	0.221161	0.824967	NA
Cd200r1	3.8695903	-0.116586036	0.604804	-0.19277	0.847142	NA
Cd209e	4.0702325	0.224765557	0.527605	0.426011	0.6701	NA
Cd244a	6.641867	0.269366435	0.41908	0.642756	0.520382	NA
Cd3d	5.9403729	0.097480131	0.450264	0.216496	0.828602	NA
Cd3e	2.0971661	0.260114603	0.745096	0.349102	0.727013	NA
Cd3g	4.3768588	0.677229341	0.500252	1.353776	0.175808	NA
Cd4	5.3530604	0.054485082	0.582311	0.093567	0.925453	NA
Cd40	7.6569457	0.317738351	0.407438	0.779845	0.435482	NA
Cd8a	4.5984838	0.468894942	0.550489	0.851779	0.394337	NA
Cd8b1	4.4598151	0.833849175	0.550821	1.51383	0.130069	NA
Ceacam3	4.4145331	-0.647052069	0.521685	-1.24031	0.21486	NA
Cfi	5.4901184	0.390784571	0.487871	0.800999	0.423132	NA
Ctsw	4.58687	0.056071267	0.522635	0.107286	0.914562	NA
Cxcl15	5.7306602	0.162022829	0.458799	0.353145	0.72398	NA
Dlx2	6.0302567	-0.181810927	0.465441	-0.39062	0.696078	NA
Eomes	4.1149782	-0.048338223	0.591348	-0.08174	0.934852	NA
Fcrlb	5.4421261	0.433731275	0.447191	0.969901	0.332096	NA
Foxp3	2.9546189	-0.0598239	0.607029	-0.09855	0.921494	NA
Gzma	6.7072407	0.085007009	0.433959	0.195887	0.844698	NA
Gzmb	5.4740743	-0.138326422	0.469833	-0.29442	0.76844	NA
Il10	4.5636805	0.594058048	0.521502	1.139128	0.25465	NA
Il1a	6.6132454	-0.163321154	0.466251	-0.35029	0.726124	NA
Il1b	5.0501131	0.800412976	0.540458	1.48099	0.138609	NA
Il4	3.8832648	0.171054127	0.552784	0.309441	0.756986	NA
Il6	7.1409686	-0.008056738	0.416181	-0.01936	0.984555	NA
Itgax	4.4668189	-0.326400091	0.557488	-0.58548	0.558223	NA
Kir3dl1/2	4.388706	-0.05980622	0.491939	-0.12157	0.903238	NA
Klrk1	4.0787768	0.499498461	0.512394	0.974832	0.329643	NA
Ltc4s	6.4533089	0.167014271	0.412786	0.404602	0.68577	NA
Mapk13	6.389012	0.552578088	0.430205	1.284452	0.198984	NA

Ms4a1	5.8444248	0.364654944	0.431857	0.844388	0.398452	NA
Ms4a2	7.6610195	-0.189541372	0.391594	-0.48403	0.628368	NA
Msr1	6.4759405	0.783636991	0.539283	1.453109	0.146193	NA
Mx1	6.4837187	-0.575298106	0.455435	-1.26318	0.206523	NA
Ncr1	3.6119418	-0.967039089	0.631589	-1.53112	0.12574	NA
Nkg7	5.9654923	0.452465238	0.423071	1.069479	0.284854	NA
Nlrp1b	6.2805151	-0.295490751	0.439143	-0.67288	0.501023	NA
Nos2	4.1910438	-0.022834394	0.540492	-0.04225	0.966301	NA
Prf1	7.2544542	0.637652726	0.424939	1.500575	0.133466	NA
Ptger4	8.0367592	0.169981485	0.476008	0.357098	0.721018	NA
Pth2r	6.1993698	0.001785869	0.448274	0.003984	0.996821	NA
Sh2d1a	7.456335	0.55225213	0.374853	1.473248	0.140684	NA
Slc10a6	8.0122975	0.032663141	0.409499	0.079764	0.936425	NA
Spib	7.9455336	0.571493912	0.422849	1.351531	0.176525	NA
Tbr1	2.5971005	0.520713221	0.637899	0.816294	0.414332	NA
Tbx21	1.5884473	-0.478141379	0.815661	-0.5862	0.557741	NA
Tcl1	8.1404467	0.565761174	0.384256	1.472354	0.140925	NA
Tfec	3.8642807	-0.270826301	0.603732	-0.44859	0.65373	NA
Tgm1	5.0049979	-0.47111464	0.539948	-0.87252	0.382926	NA
Tnf	4.5308092	0.477997447	0.510821	0.935744	0.349405	NA
Tnfrsf17	4.7552457	-0.614478718	0.562268	-1.09286	0.274457	NA
Tpsab1	3.1903298	0.125942009	0.607048	0.207466	0.835646	NA
Tpsb2	4.0727047	0.568226345	0.592866	0.95844	0.337841	NA
Trat1	3.501587	-0.007070737	0.658973	-0.01073	0.991439	NA
Xcl1	3.146581	-0.072328346	0.648944	-0.11146	0.911255	NA
