# Supplementary material for: MhcII Regulates Transmission of α-Synuclein-Seeded Pathology in Mice
Source: Int J Mol Sci. 2022 Jul 25;23(15):8175. doi: 10.3390/ijms23158175 (PMC9332117; doi:10.3390/ijms23158175)
Supplement: Supplementary file 1 [file ijms-23-08175-s001.zip › Supplementary Figures and Figures Legends.pdf]

Supplementary Figures and Figures Legends:

MhcII Regulates Transmission of α-Synuclein-Seeded Pathology in Mice

Elsa Gonzalez De La Cruz, Quan Vo, Katie Moon, Karen N. McFarland, Mary Weinrich,

Tristan Williams, Benoit I. Giasson and Paramita Chakrabarty.

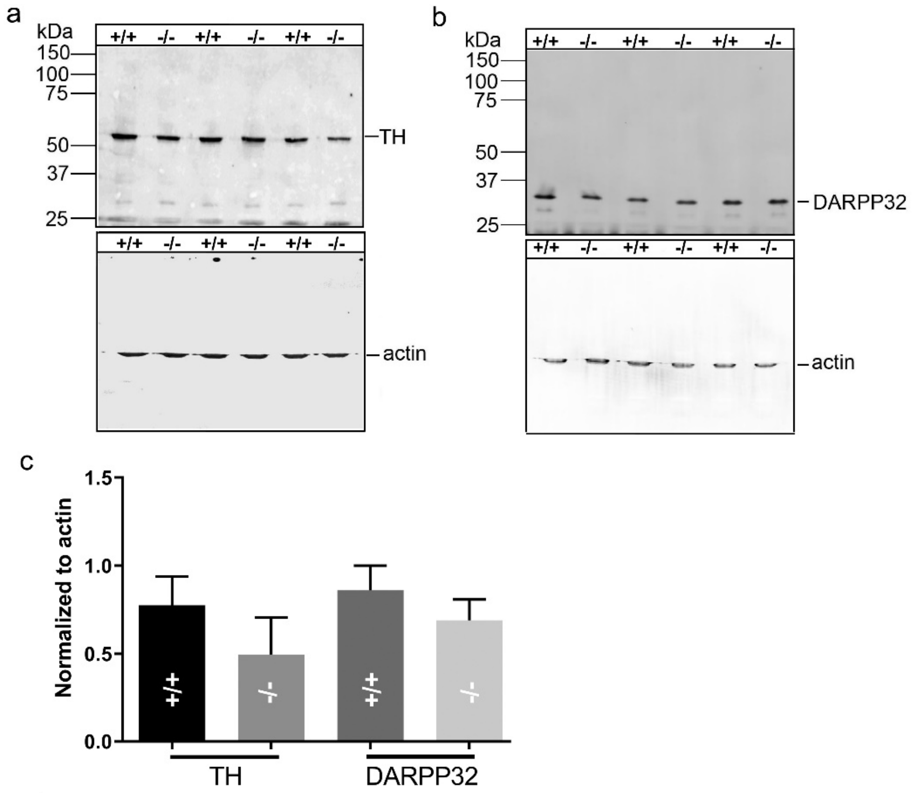

d

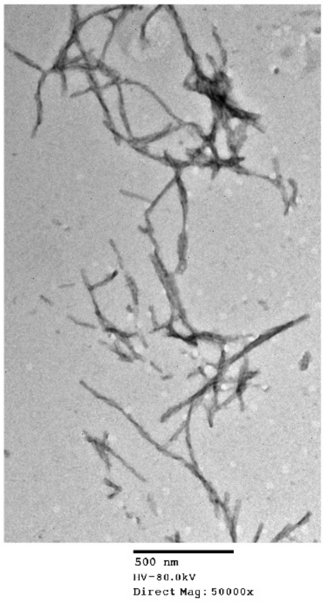

e

Endpoint Fluorescence

	Ex	Em	Cutoff
Lm1	380	550	530

Sample ID	1	2
K114 only	68.321	64.292
αSyn PFF+K114	1056.7	860.95
PBS	0.984	0.872

Supplementary Figure S1. Characterization of α-syn PFFs and assessment of TH and DARPP32 levels in PFF-seeded MhcII+/+ or MhcII-/- mice at end stage.

a-c. MhcII+/+ or MhcII-/- mice were seeded with α-syn PFFs at 2 months of age and were analyzed at end-stage using antibodies against tyrosine hydroxylase (TH) or DARPP32. Immunoblotting using soluble brain lysates (a-b) and protein levels standardized to housekeeping gene, actin (c) shown. n=3 mice/group. d. Transmission EM images of WT mouse αSyn PFF. Recombinant αSyn PFFs stained with uranyl acetate and visualized. Scale bar, 500 nm. e. Representative K114 fluorescence analysis of αSyn PFFs. Samples were analyzed in duplicate (indicated by columns 1, 2) and raw fluorescence units shown. Fluorescence parameters are indicated on top of the table.

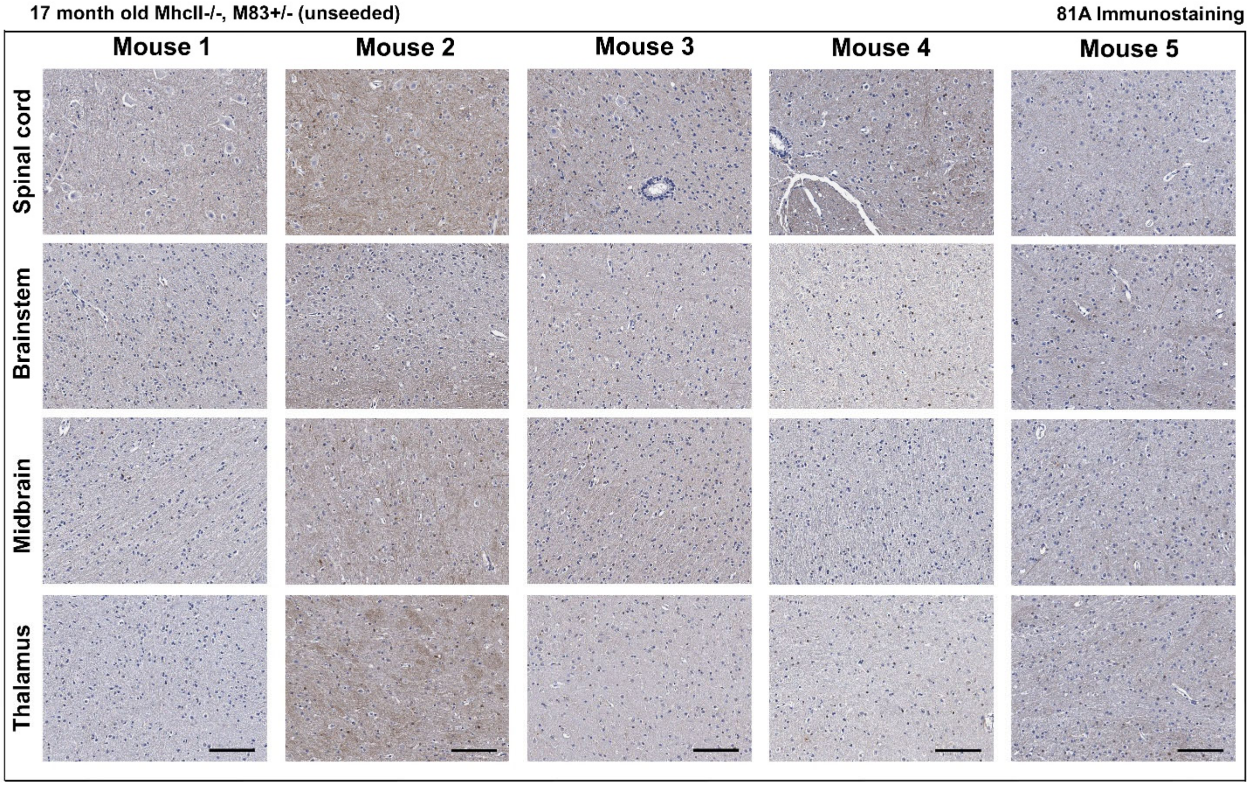

Supplementary Figure S2. Absence of intraneuronal 81A staining in non-seeded old *MhcII*-/- mice.

M83+/- mice lacking both *MhcII* alleles were aged to 17 months and immunostained with pSer129 α-syn antibody. Representative sections from five mice stained with 81A antibody is shown. Scale bar, 200μm.

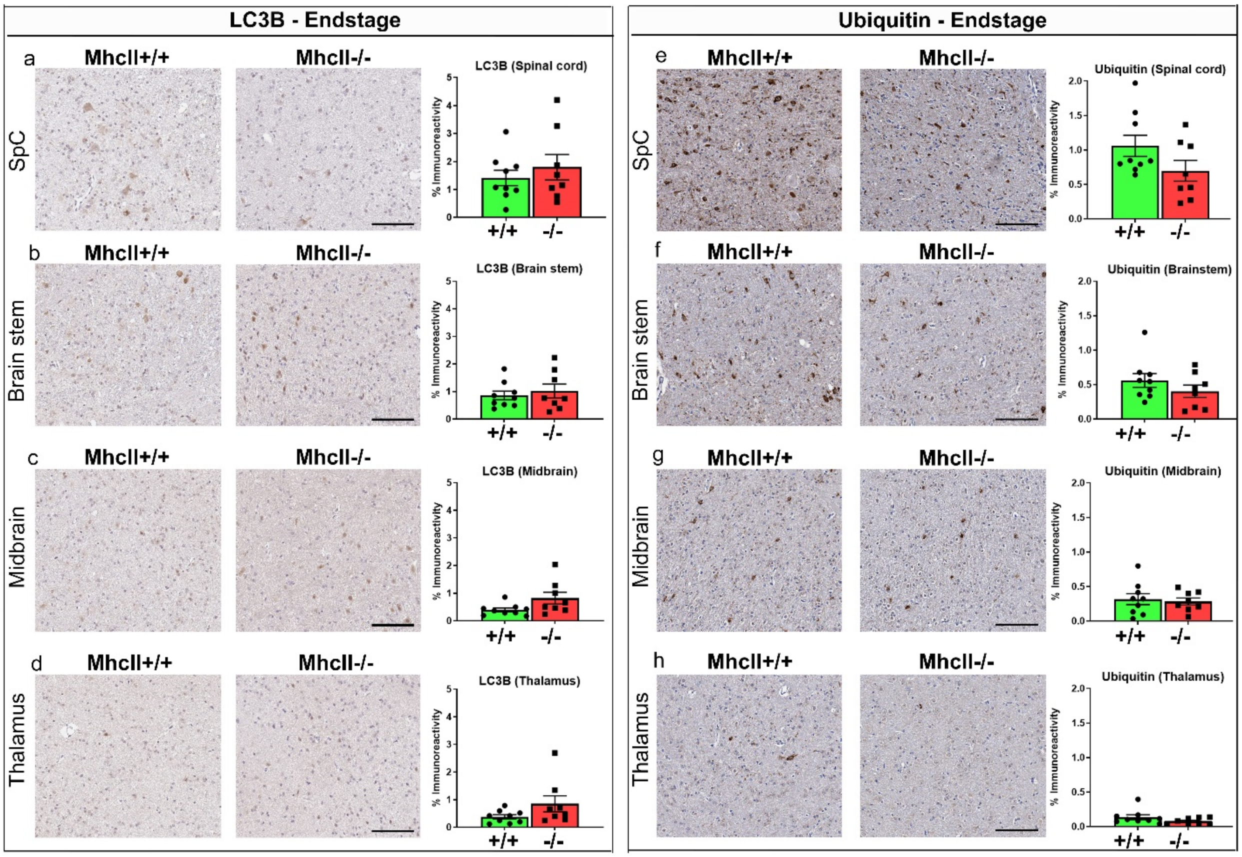

Supplementary Figure S3. LC3B and ubiquitin levels in PFF-seeded mice.

MhcII+/+ or *MhcII*-/- mice were seeded with α-syn PFFs at 2 months of age and were analyzed at end-stage (LC3B, a-e; Ubiquitin, e-h). Representative images of immunostained tissue sections from spinal cord (gray and white matter), brain stem, midbrain and thalamus shown. SpC, spinal cord. Scale bar, 100 µm; inset, 50 µm. n=7-8 mice/group.

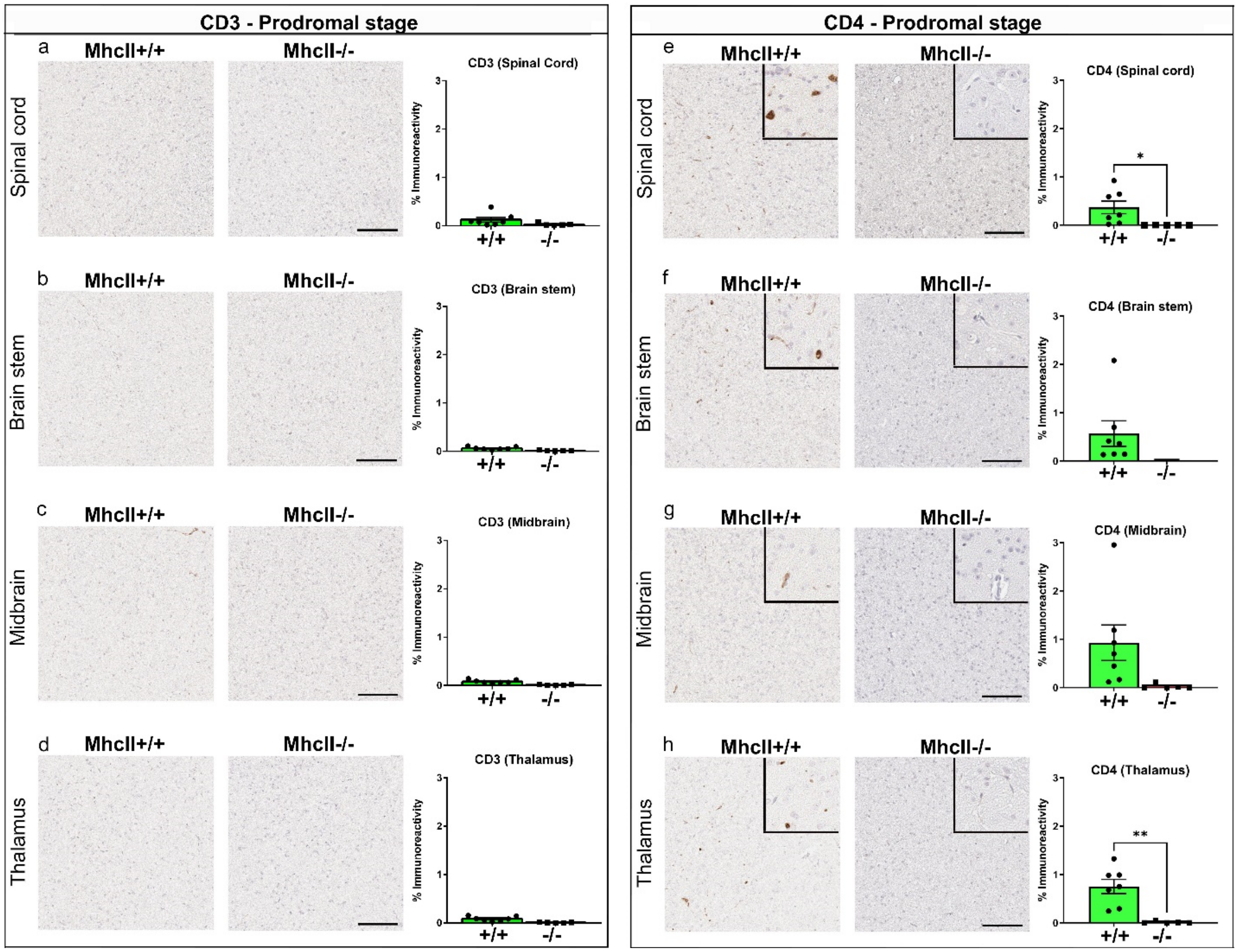

Supplementary Figure S4. Absence of CD3+ and CD4+ T cells in *MhcII*-/- mice.

MhcII+/+ or *MhcII*-/- mice were seeded with α-syn PFFs at 2 months of age and were analyzed at a prodromal stage 2 months post-PFF seeding. Representative images of anti CD3 (a-d) and anti CD4 (e-h) antibody-stained tissue sections from spinal cord, brain stem, midbrain and thalamus shown. *MhcII*-/- mice had no detectable CD3+ or CD4+ immunostaining. Scale bar, 100 μm; inset, 50 μm. n=5-6 mice/group. Unpaired 2 tailed t test. *p<0.05; **p<0.01; ***p<0.001.

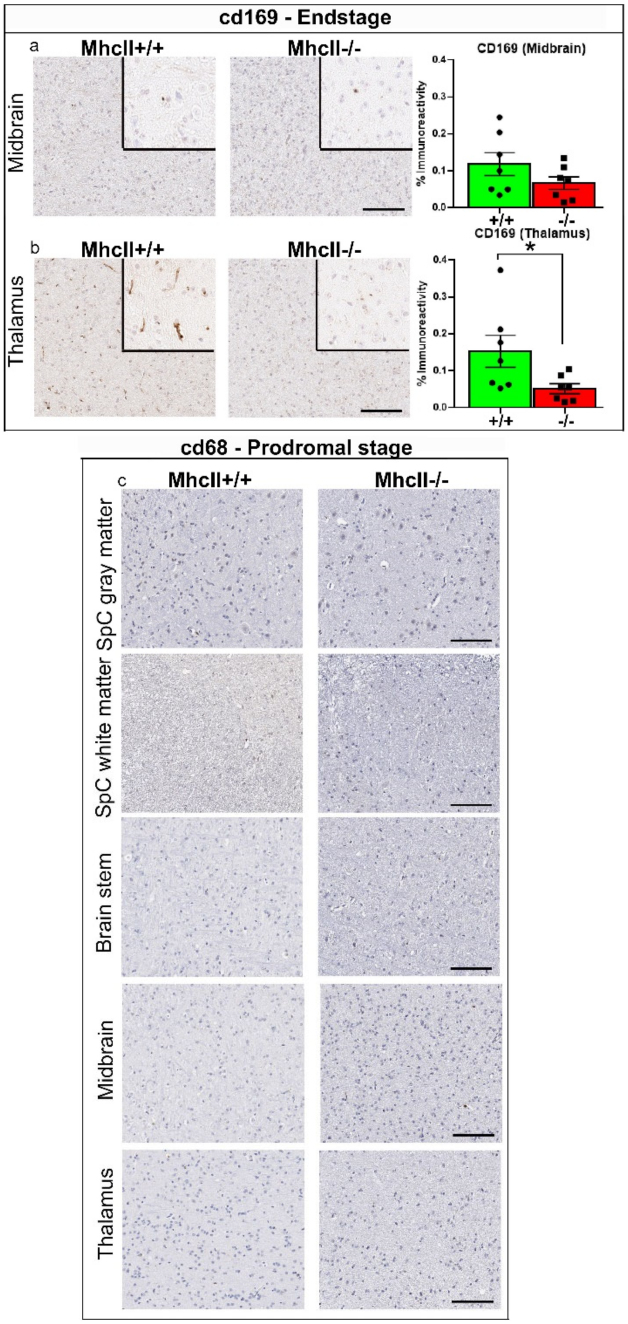

Supplementary Figure S5. Macrophage burden in PFF-seeded *MhcII*+/+ or *MhcII*-/- mice.

MhcII+/+ or *MhcII*-/- mice were seeded with α-syn PFFs at 2 months of age. CD169 stained representative panels and magnified detail (inset) are shown from end stage *MhcII*+/+ or *MhcII*-/- mice (a-b). CD68 stained representative panels are shown from prodromal stage *MhcII*+/+ or *MhcII*-/- mice (c). Since there was no immunoreactive cells present, burden analysis was not done. SpC, spinal cord. Scale bar, 100 µm; inset, 50 µm. n=7-8 mice/group (endstage group) and n=5-6 mice/group (prodromal group). Unpaired 2 tailed t test.

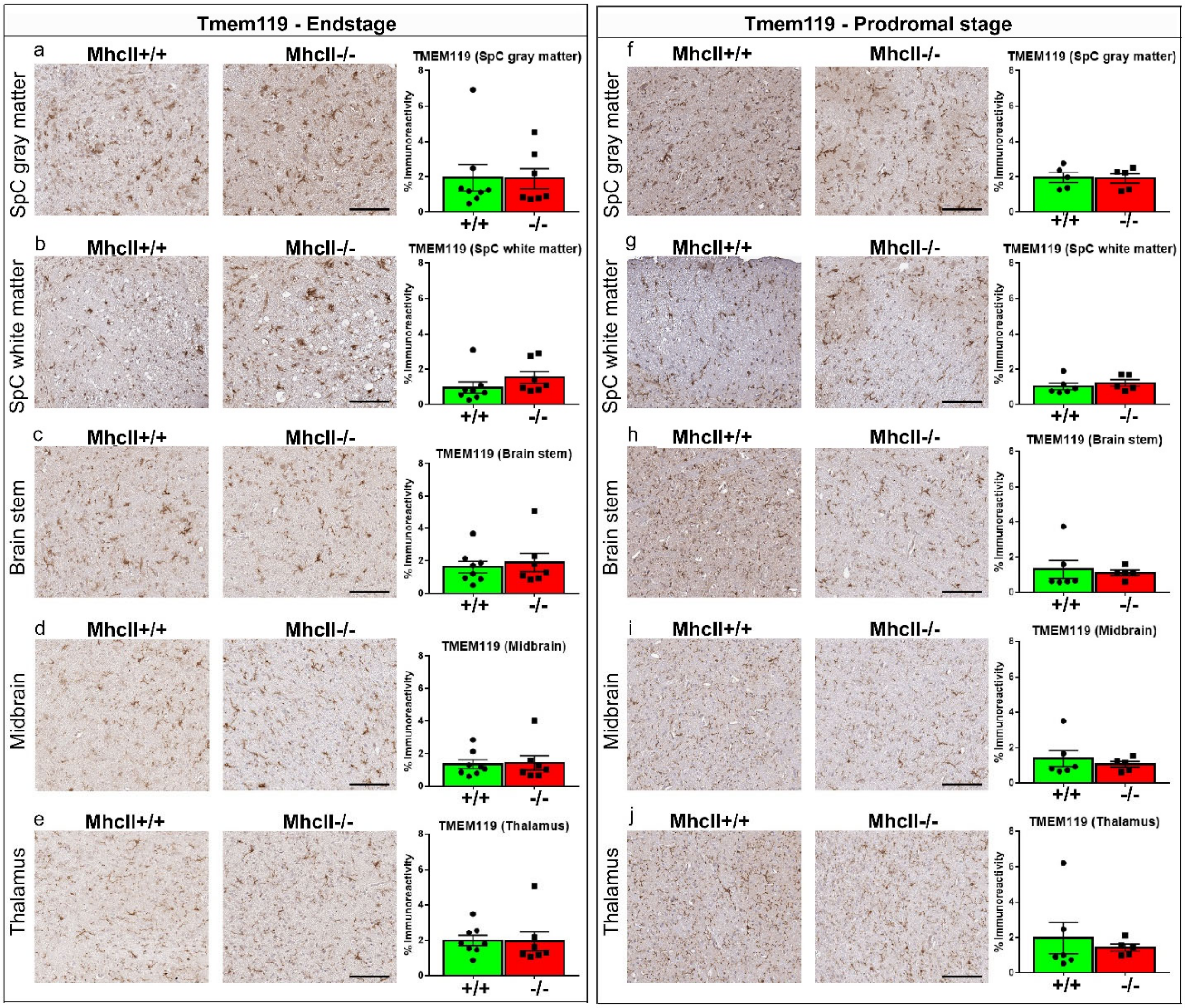

Supplementary Figure S6. Homeostatic microglia burden in PFF-seeded *MhcII*+/+ or *MhcII*-/- mice.

MhcII+/+ or *MhcII*-/- mice were seeded with α-syn PFFs at 2 months of age and were analyzed at end-stage (a-e) or at a prodromal stage 2 months post-PFF seeding (f-j). Representative images of anti Tmem119 antibody-stained tissue sections from spinal cord, brain stem, midbrain and thalamus shown. SpC, spinal cord. Scale bar, 100 µm; n=7-8 mice/group (a-e) and n=5-6 mice/group (f-j). Unpaired 2 tailed t test.
